# Synthesis and Antiviral Evaluation of Unexplored Dioxolane-Derived 7‑Deazapurine Nucleoside Analogues against Epstein–Barr Virus (EBV)

**DOI:** 10.1021/acsmedchemlett.5c00397

**Published:** 2025-08-12

**Authors:** Uma S. Singh, Ransom A. Jones, Yugandhar Kothapalli, Shuiyun Lan, Xing-Quan Zhang, Ryan L. Slack, Harischandra P. Thoomu, Robert T. Schooley, Stefan G. Sarafianos, Chung K. Chu

**Affiliations:** † Department of Pharmaceutical and Biomedical Sciences, College of Pharmacy, 1355 University of Georgia, Athens, Georgia 30602, United States; ‡ Center for ViroScience and Cure, Laboratory of Biochemical Pharmacology, Department of Pediatrics, 12239 Emory University School of Medicine, Atlanta, Georgia 303322, United States; § Department of Medicine, 8784 University of California, San Diego, La Jolla, California 92093, United States

**Keywords:** Dioxolane-derived nucleosides (DDA), Epstein−Barr virus (EBV), Multiple Sclerosis (MS), Antiviral, Nucleoside Analogues

## Abstract

Dioxolane-based nucleosides are characterized by their unique replacement of the sugar moiety by dioxolane-based cyclopentyl rings. A series of d-dioxolane-derived 7-deazapurine analogues (**12**–**19**) were synthesized and evaluated against Epstein–Barr virus (EBV) and human immunodeficiency virus (HIV). The 7-bromo-deazaadenosine analogue (**15**) demonstrated an EC_50_ of 0.17 μM and the 7-iodo-deazaadeosine analogue (**16**) displayed an EC_50_ of 0.47 μM, compared to ganciclovir (EC_50_ = 2.5 μM) against EBV. Compound **15** was 14 times more potent than ganciclovir, with a high selectivity index (SI = 294). Additionally, the deazaadenosine analogue (**12**) and 7-fluoro-deazaadenosine (**13**) have shown moderate antiviral potency against HIV. The reported analogues of this series expressed both potency and selectivity against EBV. The development of prodrugs for analogues **12**, **13**, **15**, and **16** may potentially enhance their antiviral activity against EBV and HIV, offering a promising avenue for identifying preclinical candidates effective against both DNA and RNA viruses.

## Graphical abstract

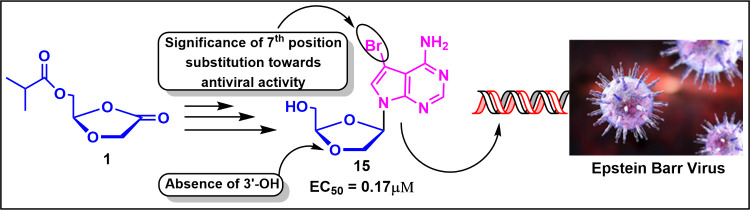

Herpesviruses pose a significant threat to humans, due to their ability to establish lifelong latent infections.^1^ These viruses may cause life-threatening diseases in the case of both primary infections as well as on reactivations.^2^ Epstein–Barr virus (EBV), a member of the *Herpesviridae* family, is a major cause of infectious mononucleosis and contributes significantly to morbidity in adolescents and young adults.^3^ It is speculated that most people become infected with EBV during their lifetime. EBV is a ubiquitous virus and was first discovered in the tumor cells of pediatric Burkitt Lymphoma.^4^ Later, researchers revealed that EBV stimulates a variety of lymphoproliferative disorders.^5^ Its latent infections are linked to the development of cancer and autoimmune diseases.^6^ Recent studies have also shown that EBV infection can logically and mechanistically enhance the progress of multiple sclerosis (MS). MS is a prevalent chronic inflammatory and neurodegenerative disease of the central nervous system (CNS),^7^ and it is triggered by the infection of the EBV.^8^ Globally, approximately 2.8 million people have MS,^9^ and recent findings of a longitudinal analysis on the U.S. troops (active adults on duty) have revealed a high prevalence of EBV associated with MS.^10^ Rather than infectious mononucleosis, the risk of MS increases 32-fold after EBV infection.^10^ Notably, worldwide, ∼90% of adults have been infected with EBV, and it is constantly detected in numerous cancers, including nasopharyngeal carcinoma, subtypes of Hodgkin and non-Hodgkin lymphomas,^11^ EBV-associated gastric carcinoma, leiomyosarcoma, and natural killer (NK)/T cell carcinomas.^12^ In EBV-driven cancers, MS, and fatal lymphoproliferative disorders, the reactivation of the virus plays a critical role. Additionally, organ transplant patients on immune suppressant therapy also face a severe life-threatening condition after latent EBV infection.^13^ In the case of organ transplant patients, EBV causes post-transplant lymphoproliferative disease (PTLD).^14^ PTLD conditions create a life-challenging situation for organ transplant patients and generate a critical concern for treatment and safe organ transplantations.^14^ There is no approved antiviral to specifically treat EBV or its associated MS conditions.^15^ Therefore, a significant population with underlying EBV infections, including immunocompromised and cancer treatment patients, faces a heightened risk from the virus. Broad-spectrum antivirals such as acyclovir (ACV), valacyclovir (VACV), penciclovir (PCV), famciclovir (FCV), and ganciclovir (GCV, Figure 1) are being used for the treatment of EBV infections.^16^ These FDA-approved antivirals have less potency against EBV, and often, large doses of prescribed drugs promote severe side effects and viral mutations.^16^ Furthermore, especially against MS conditions, approved antivirals have not demonstrated any clear benefits.^15^

**Figure 1:**
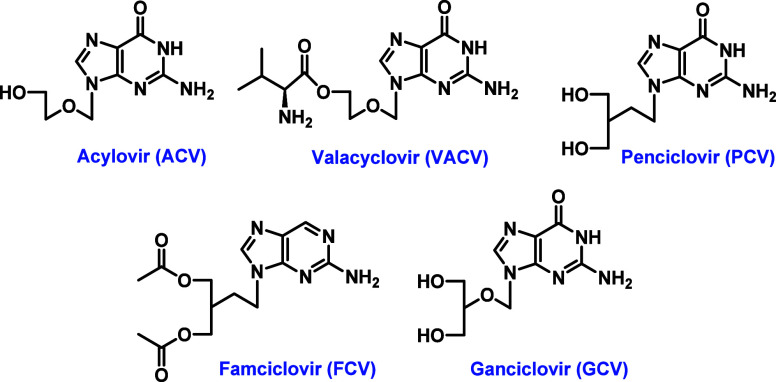
Structures of drugs currently used for the treatment of EBV infection.

Consequently, there is a void for potent antivirals, and a new direct-acting antiviral (DAA) agent is urgently needed to overcome EBV-associated complications. The discovery of new antivirals will also assist in the future preparedness to deal with this life-threatening DNA virus, which establishes a lifelong infection. Elaborating on our past two-decade efforts, in this communication, an extended structure–activity relationship (SAR) of unexplored d-dioxolane-7-deazapurine analogues (**12**–**19**) has been reported, which demonstrated strong inhibition of EBV and moderate activity against HIV. From these synthesized analogues, compounds **15** and **16** exhibited potent antiviral activity and will proceed for further preclinical development against EBV and other herpesvirus infections. The timely finding of this report may accelerate the space of altered dioxolane-derived nucleosides as preclinical candidates against EBV and other emerging viruses.

For the past two decades, our group has been continuously exploring dioxolane nucleos­(t)­ides that have shown potent antiviral and anticancer activities.^17–20^ We have previously reported numerous d- and l-dioxolane-derived nucleo­(t)­side analogues, which have shown promising antiviral activity.^21–24^ Recently, our group invented bromovinyl analogue, l-BHDU (Figure 2),^25–27^ and its phosphate ester prodrug POM-L-BHDU-MP,^17,18^ a promising preclinical candidate against varicella-zoster virus (VZV) and herpes simplex virus 1 (HSV 1) infections. Additionally, a vinyl analogue, l-HDVD, was discovered that has shown broad-antiviral potency against EBV, Kaposi sarcoma-associated herpesvirus (KSHV), and HSV 1.^24^

**Figure 2:**
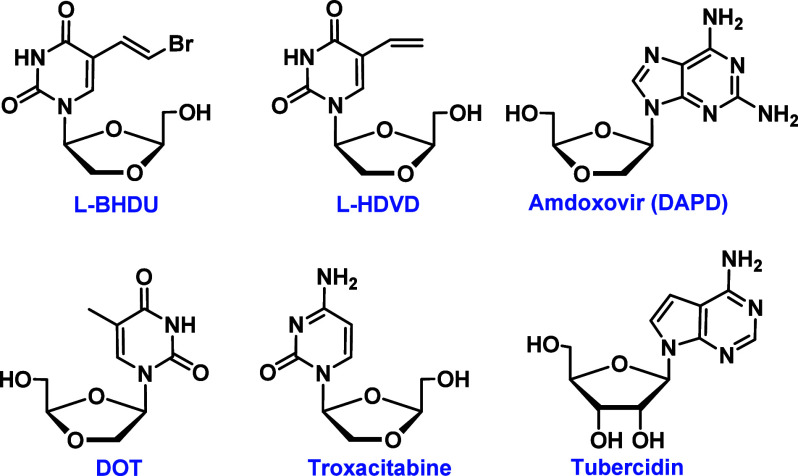
Structures of d- and l-dioxolane-derived potent antiviral nucleosides.

Two d-dioxolane-derived nucleosides—amdoxovir (DAPD, diaminopurine dioxolane nucleoside)^21^ and DOT (a dioxolane thymidine nucleoside, Figure 2)^22^—were synthesized, which demonstrated potent antiviral activity against HIV. Amdoxovir was advanced to a phase II clinical trial.^28^ Troxacitabine, an l-dioxolane pyrimidine analogue, has shown promising anticancer activity across numerous cancers and has exhibited anti-HIV and anti-HBV activity.^29,30^ Dioxolanes, heterocyclic acetal cyclopentyl rings, offer a potential replacement for sugar scaffolds, which lack the crucial 3′–OH necessary to propagate DNA/RNA elongation. Replacing a ribose with a dioxolane ring in nucleosides creates specificity and selectivity to viral DNA/RNA without causing toxicity to humans. The incorporation of a dioxolane-derived nucleotide into an elongating viral DNA chain may proceed with viral chain termination, which ultimately results in the inhibition of viral growth. Based on the same structures, oxathiolane, similar to dioxolane, although with a 3′ sulfur in lieu of oxygen, has expressed potent antiviral activity.^31^ Additionally, lamivudine and emtricitabine derived from oxathiolane-derived nucleosides have been approved for the treatment of HBV and HIV infections, respectively.^31^

Still, there is a need to generate and explore efficacious and selective dioxolane nucleosides for antiviral use, for which additional alterations on either the dioxolane ring or nucleobase are required. The increased complexity of nucleosides is associated with efforts to enhance viral specificity and selectivity. Nitrogen substitutions on the nucleobase, either in purines or pyrimidines, result in substantial effects on activity with the removal of a potential hydrogen bond acceptor or donor, which may significantly affect the therapeutic properties of the molecule. Based on similar substitution, 7-deazapurines (pyrrolo­[2,3-*d*]­pyrimidine) have gained much attention as antiviral and anticancer agents.^32^ It is a modified base analogue of adenine wherein the seventh position nitrogen is replaced by a carbon atom, which makes the five-membered ring more flexible and provides the possibility of generating additional substituents at the C-7 position.^32^ Also, the replacement of nitrogen by carbon at the seventh position of the purine ring modifies the electronic properties of the five-membered ring and results in a more-enriched electron ring system that may increase the chance of cation−π or π–π interactions at binding sites of receptors. Furthermore, substitution at the 7-position of the 7-deazapurine base dramatically changes the therapeutic properties of this class of molecules. Tubercidin, a 7-deazaadenosine analogue (Figure 2), and its derivatives have shown nanomolar potency against hepatitis C virus (HCV) and cancer.^32^ Taking the lead from the above-described molecule, several analogues of modified 7-deazapurines nucleoside with a d-dioxolane (**12**–**19**) have been synthesized, and their antiviral potency was evaluated against EBV and HIV.

To accomplish the synthesis of targeted 7-deazapurine dioxolane nucleoside analogues (**12**–**19**), dioxolane ketone **1** was synthesized according to the literature reported protocol by Sznaidman et al.^33^ Reduction of ketone **1** was performed with a 1 M solution of lithium tri-*tert*-butoxyaluminum hydride (LTBA) in THF to afford the racemic alcohol **2** (Scheme 1). Compound **1** was treated with LTBA at −15 °C to produce alcohol **2**. First, attempts were made to purify racemic alcohol **2**. However, during the purification of **2** via silica gel chromatography, a degradation of **2** was observed. Therefore, it was thought to move with an in-situ acetylation of **2**, providing stable acetylated compound **3**. Alcohol **2** was treated with acetic anhydride in the presence of 4-(dimethylamino) pyridine (DMAP) to give a diastereomeric mixture of α and β isomers (2:1 ratio) of **3** in 39% yield. The α/β diastereomers of **3** were inseparable via column chromatography and were utilized as a mixture for the next-step coupling reaction.

**Scheme 1:**
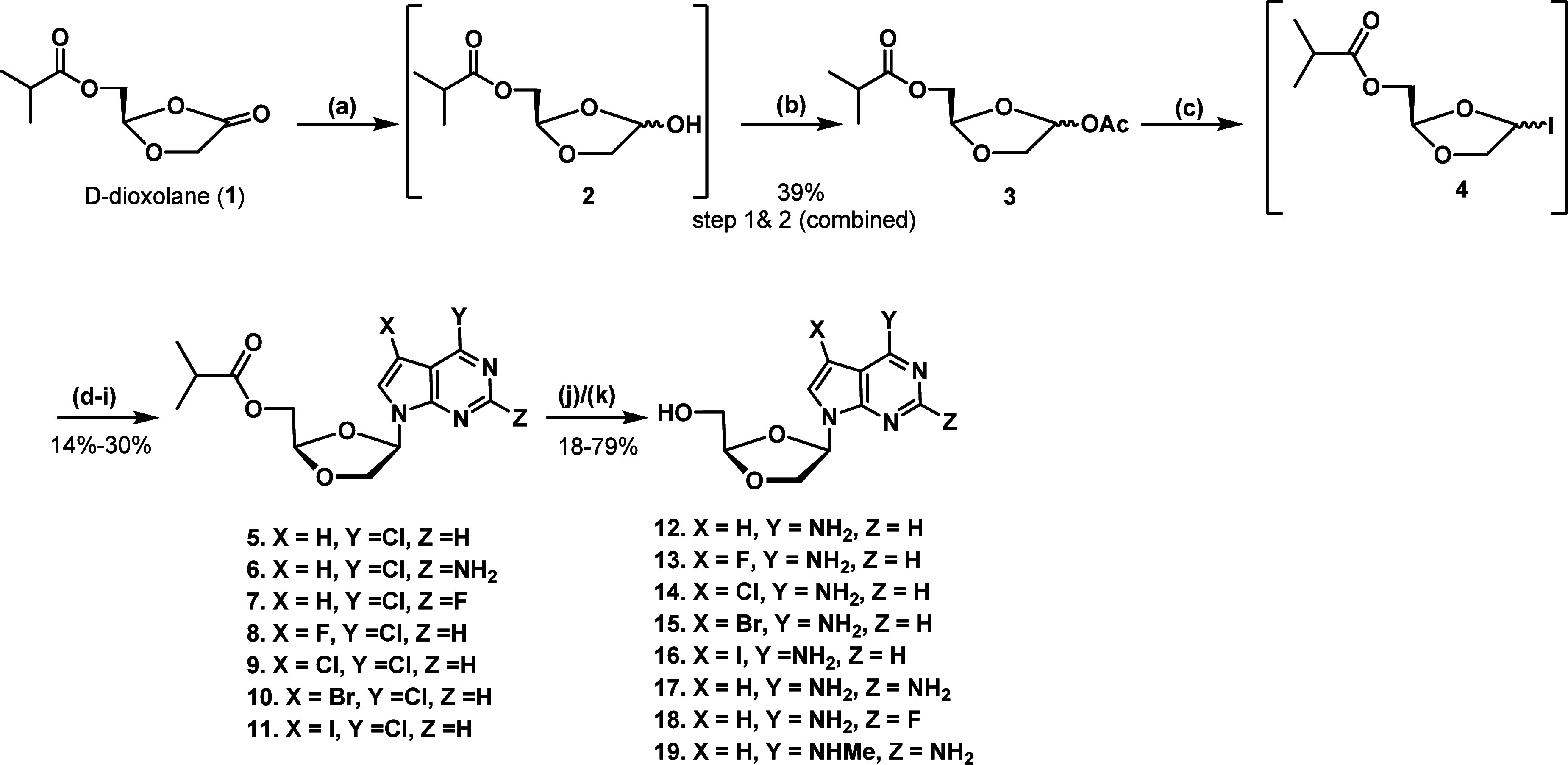
Synthesis of Targeted Compounds **12**–**19** via d-Dioxolane Ketone (**1**)­^a^

a Reagents and conditions: (a) 1 M LTBA, THF; (b) DMAP, Ac_2_O, THF; (c) TMSI, CHCl_3_; (d) Appropriate substituted 7-deazapurines, KOH, TDA-1, ACN, ; (j) NH_4_OH, 1,4-dioxane; (k) methylamine, EtOH.

Coupling dioxolane acetate **3** with the 7-deazapurines proved to be a tedious and extensively challenging process. In initial attempts, following our earlier reported protocol^21,30^ 6-chloro-7-deazapurine was refluxed in hexamethyldisilazane (HMDS) with catalytic ammonium sulfate to produce the silylated 7-deazapurine, which was used immediately in the next step. Compound **3** was treated with iodotrimethylsilane (TMSI) to produce intermediate **4**, followed by the immediate addition of the silylated 7-deazapurine.

However, multiple attempts had been made for the coupling of **4** with an appropriate silylated 6-chloro-7-deazapurine, but in each case, a complex mixture was obtained. Therefore, it was concluded that the seventh position nitrogen on the purine ring might be essential for silylation, and the absence of the seventh position nitrogen in the case of 7-deazapurine may inhibit the coupling reaction and result in multiple spot formation. Next, an anion-generated S_N_2 approach was adopted for the coupling of 6-chloro-7-deazapurine with intermediate **4**. In this attempt, 6-chloro-7-deazapurine was treated with NaH (60% in oil) in acetonitrile (ACN), and to this solution, freshly prepared **4** was added dropwise, and the mixture was subjected to stirring for 3 h to afford a coupled nucleoside intermediate **5** in a poor yield of 4%–5%. However, four major products were formed in this coupling condition rather than the expected two 1′ α/β-products (intermediate **5**). It was speculated that the additional products may be the result of epimerization at the 4′ position of **5**. Furthermore, potassium hydroxide (KOH) with phase-transfer catalyst conditions was tried to provide the major β-coupled nucleoside product.^34^ 6-Chloro-7-deazapurine was treated with KOH in the presence of the phase-transfer catalyst tris­[2-(2-methoxyethoxy)­ethyl]­amine (TDA-1) in acetonitrile (ACN), and to this, freshly prepared **4** was added to produce desired β-coupled intermediate **5** in 14% yield. This altered coupling method produced exclusively two diastereomeric major 1′ α and β coupled products, which were separated via flash silica gel column chromatography. The coupling method of **4** with an appropriate substitute 7-deazapurine analogue in the presence of KOH was found to be efficient and repeatable. This method was utilized to construct other coupled intermediates (**6**–**11**) with a yield range of 14%–30% (Scheme 1). To furnish the desired targeted final nucleoside **18**, 4-chloro-2-fluoro-7*H*-pyrrolo­[2,3-*d*]­pyrimidine was synthesized by fluorination via Sandmeyer reaction, as per the reported protocol by Babu et al.^35^

Furthermore, the β-conformation of the coupled nucleosides was determined via similarly reported NMR data of dioxolane-derived purine nucleosides.^30^ ^1^H NMR data showed significant differences in shifting for the position of 4′ proton based on either 1′ α or β conformation of the coupled nucleosides.^30^ Likewise, this relationship was also observed in the synthesized intermediates of 7-deazapurine dioxolane nucleosides (**5**–**11**). Interestingly, it was observed that the β conformation compounds were crystallized at room temperature, while the α coupled product remained as a viscous oil.

Deprotection and 6-position amination of compounds **5**–**11** were carried out to afford the desired targeted nucleosides. In a steel bomb under higher pressure, compounds **5**–**11** were treated with a 28% aqueous solution of NH_4_OH in dioxane to render the final targeted nucleosides in 18%–79% yield. During the amination step, ester hydrolysis was simultaneously performed to produce the final compounds (**12**–**18**). Notably, to synthesize compound **19**, intermediate **6** was treated with 33% of *N*-methyl amine in ethanol instead of an aqueous solution of NH_4_OH to give *N*-methyl prodrug of guanosine analogue **19** in 40% yield. Furthermore, the X-ray crystal structure of compound **12** (Figure 3) was determined to confirm the β confirmation of the synthesized final targeted compounds. The crystallographic analysis of **12** revealed the *cis* conformation of hydrogens attached to C-7 and C-9, which confirms the β conformation of synthesized analogs (**12**–**19**).

**Figure 3:**
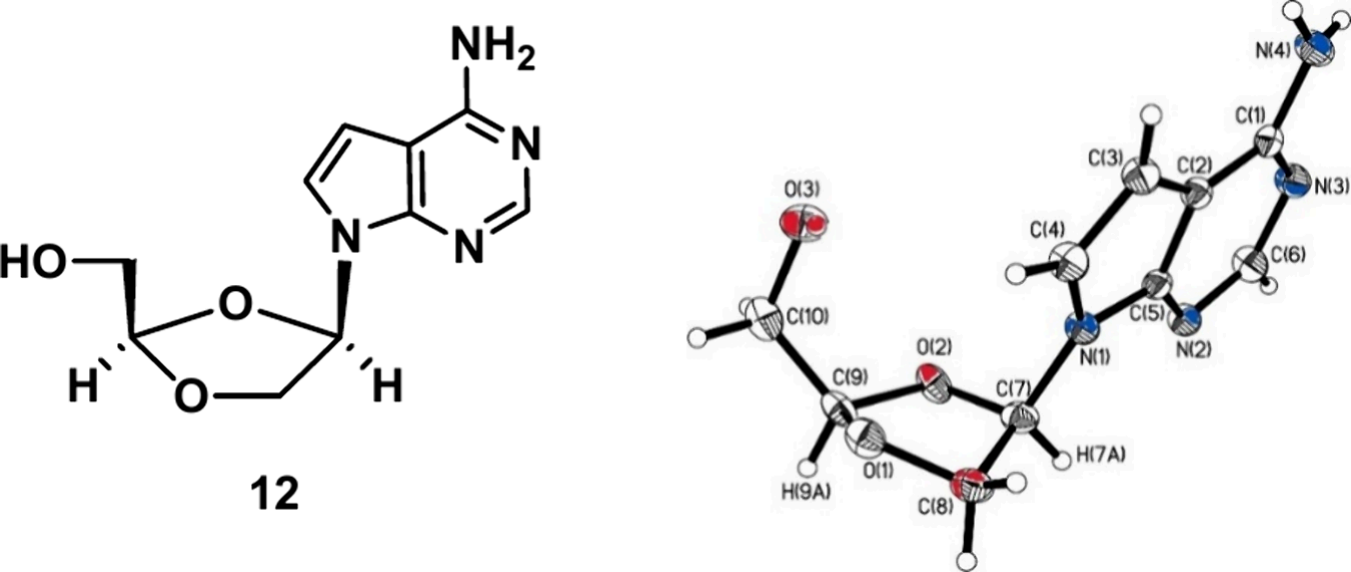
ORTEP structure of compound **12** (CCDC No. 2452696) demonstrates that the hydrogens at C(7) and C(9) are *cis* to each other, which confirms its relative β-stereo conformation.

The antiviral potency of all synthesized nucleosides was evaluated by in-vitro cell-based assays. P3HR-1 cells were seeded in 24-well plates at a density of 1 × 10^6^ cells/mL. EBV replication was induced by adding 25 ng/mL 12-*O*-tetradecanoylphorbol 13-acetate (TPA) (Sigma–Aldrich, Bornem, Belgium) to the growing cells. The next day, cells were washed and resuspended in fresh medium in the presence or absence of various concentrations of synthesized nucleosides (**12**–**19**). Ganciclovir (GCV) was used as antiviral control. On day 5 post-induction, total cellular DNA was extracted (QIAamp DNA kit; Qiagen Benelux B.V, Venlo, The Netherlands), and viral DNA was quantified by quantitative PCR (qPCR) using an ABI Prism 7500 Sequence Detection System (Applied Biosystems, Foster City, CA). Thermocycling conditions for all qPCRs were done following the manufacturer’s instructions. Forward and reverse primers, as well as TaqMan probe sequences allowing the detection of the target BNRF1 of EBV, have been described by Friedrichs et al.^36^ The concentrations required to effectively reduce EBV DNA synthesis in TPA-stimulated cells by 50% and 90% (EC_50_ and EC_90_) were extrapolated from the standard curve using linear regression analysis. The results were obtained as a means from at least three independent experiments. The selectivity index of the compound was determined by the ratio of CC_50_/EC_50_. For both efficacy and cytotoxicity assays, the 50% reduction in efficacy (EC_50_) and cell viability (CC_50_) was determined using GraphPad software (GraphPad, San Diego, CA, USA, www.graphpad.com). From the synthesized nucleoside analogues, **15** demonstrated an EC_50_ of 0.17 μM with a CC_50_ of 49.9 μM against EBV, compared to GCV (EC_50_ = 2.5 μM, Table 1). 7-Bromo-deazapurine-d-dioxolane nucleoside (**15**) expressed 14 times higher potency, with an SI of 294 than the FDA-approved drug GCV (SI > 40, Table 1). Furthermore, the extended SAR revealed that the substitution of fluoro at the 7-position also resulted in an improved antiviral profile for compound **13** (EC_50_ of 1.2 μM) compared to GCV. However, 7-chloro substitution at deazapurine (**14**) resulted in diminished antiviral activity (EC_50_ = 12.5 μM); on the other hand, 7-iodo substitution (**16**) retained good antiviral activity with an EC_50_ of 0.47 μM (SI = 45). Compounds **15** and **16** expressed a better antiviral profile against EBV (Figure 4).

**Table 1:** Antiviral Activity of 7-Deazanucleosides (**12**–**19**) against EBV in P3HR-1 Cell Lines and HIV-FL in TZM-GFP Cells.^a^

		Antiviral Activity against EBV (in P3HR-1 Cells)				Antiviral Activity against HIV-FL (in TZM-GFP Cells)		
sample	compound	EC_50_ ^a^ (μM)	EC_90_ ^b^ (μM)	CC_50_ ^c^ (μM)	SI^d^ (CC_50_/EC_50_)	EC_50_ ^e^ (μM)	CC_50_ ^c^ (μM)	SI (CC_50_/EC_50_)
1	**12**	>25	>25	91.3	NC	5.85 ± 1.01	>100	>17
2	**13**	1.2 ± 1.8	2.28 ± 1.29	71.3 ± 25.6	59	3.96 ± 0.39	>100	>25.2
3	**14**	12.5 ± 0.8	>25	>100	8	28.7 ± 0.45	88.02	–
4	**15**	0.17 ±. 09	0.54 ± 0.36	49.9	294	20.8 ± 0.25	41.92	–
5	**16**	0.47 ± 0.2	1.5 ± 0.09	21.2	45	10.35 ± 0.39	11.87	–
6	**17**	11.9 ± 0.3	NC^a^	>100	>8	ND^a^	–	–
7	**18**	15.5 ± 3.7	>23.9	91.7	6	ND	–	–
8	**19**	>25	NC	13.3	NC	ND	–	–
9	GCV	2.5 ± 0.22	16.24 ± 0.21	>100	>40	–	–	–
10	PF-74 ^37,38^	–	–	–	–	0.60 ± 0.05	>100	>166
11	l-HDVD^24^	0.12 ± 0.01	0.52 ± 0.04	>100	>833	ND	ND	ND

a NC = not calculated; ND = not determined.

b 50% inhibitory concentration of tested compound that requires inhibit 50% viral DNA synthesis, mean from at least three experiments.

c 90% inhibitory concentration of tested compound that requires inhibit 90% viral DNA synthesis, mean from at least three experiments.

d The drug concentration required to reduce the cellular viability by 50% after 48 h, as assayed by an XTT or MTT assay.

e Selectivity index = CC_50_/EC_50_.

f 50% inhibitory concentration 48 hpi determined by bioluminescence imaging, mean from at least three experiments.

**Figure 4:**
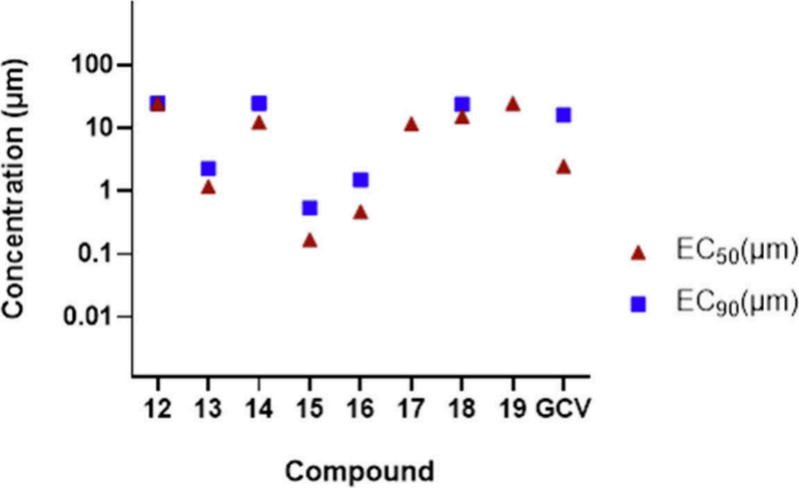
Antiviral activity of compounds against EBV. Compounds were evaluated against EBV in P3HR-1 cells. Triangle symbol (▲) represents the EC_50_ value; square symbol (■) represents the EC_90_ value. Each symbol represents the average of three independent experiments.

The 7-position unsubstituted analogue (**12**) was found inactive against EBV. The SAR leads to the conclusion that the substitutions at the 7-position of deazapurine significantly affect the antiviral activity of this series of molecules, and substitution of various functional groups or elements at the 7-position may provide a better antiviral candidate. Furthermore, the 2,6-diamino analogue (**17**) expressed less antiviral activity, with an EC_50_ of 11.9 μM and SI > 8. The cytotoxicity profile of the 2-amino-substituted compound **17** was improved (CC_50_ > 100 μM). Therefore, it is anticipated that the 7-position substitution in 2,6-diamino analogues may result in enhanced anti-EBV activity. To understand the effect of 2-position substitution on the antiviral activity of 7-deazapurine-d-dioxolane analogues, a 2-fluoro-6-amino analogue (**18**) was synthesized, which demonstrated a milder EC_50_ of 15.5 μM. However, in the same assay, our previously synthesized compound, l-HDVD^24^ (Figure 2), has expressed an EC_50_ of 0.12 μM with a high SI (>833).

The antiviral evaluation and SAR suggest that substitution at the 2- and 7-positions or exclusively at the 2- or 7-positions on 7-deazapurine of d-dioxolane nucleosides may be beneficial in identifying a preclinical candidate against EBV and accelerating the effort of antiviral drug discovery. Additionally, the 6-*N*-methyl prodrug of the guanosine analog was synthesized (**19**) to better understand the antiviral profile of guanosine-derived analogues; however, compound **19** was found to be inactive. Given the encouraging antiviral activity of 7-deazapurine-d-dioxolane nucleosides, an extended evaluation of compounds **12**–**16** was examined against HIV in the TZM-GFP reporter cell line. First, TZM-GFP cells were seeded in the 96-well plate (10 000/well), and after 24 h, selected compounds and HIV FL virus (MOI = 0.1) were added to the plate. Reference compound PF-74 was used as a positive control. At 48 h post-infection (hpi), GFP production due to infection was measured by Cytation 5, and a 50% reduction in efficacy (EC_50_) and cell viability (CC_50_) were determined using GraphPad software (San Diego, CA, USA, www.graphpad.com).

Compounds **12** and **13** demonstrated good activity against HIV with an EC_50_ of 5.85 and 3.96 μM, compared to the positive control PF-74 (EC_50_ = 0.60 μM) without cytotoxicity up to >100 μM. The initial phosphorylation is often the rate-limiting step in activating both natural and synthetic nucleosides, posing a significant challenge to their biological activity.^39^ To bypass this step, phosphoramidate prodrugs have been utilized.^40^ Studies on the synthesis of phosphoramidate prodrugs of **12**, **13**, **15**, and **16** are underway. It is established that phosphoramidate prodrug strategies that cap the polarity of the 5′-hydroxy group of nucleosides, may also increase their pharmacokinetic profile, cellular uptake, and bioavailability, and this may be the case for 7-deazapurine-d-dioxolane analogues in this report.

To gain insight into the interactions that may explain the antiviral effect of compound **15** against EBV, a homology model of the EBV DNA polymerase BALF5 was generated. The domain in BALF5 responsible for 5′-3′ DNA polymerase activity shows approximately 45% sequence identity with the HSV-1 DNA polymerase, UL30 (see the Supporting Information (SI) in the section labeled “Method: Molecular Modeling and Compound Docking”). Using a cryo-EM structure of UL30 bound to dsDNA in a paused elongation state (PDB: 8OJ7)^41^ as a structural template, we constructed a ternary complex of BALF5/dsDNA/Mg^2+^ for docking compound **15** into the dNTP binding site. Several notable features emerge from the docking results when compared to those of the UL30/dsDNA/Mg^2+^/dATP structure (Figure 5). Active site protein residues that participate in hydrogen bonding with the incoming dATP in the PDB 8OJY structure (L721 acts as a partial donor to an oxygen atom in the dATP β phosphate group, Y722 acts as a partial donor to dATP O3′, R785 acts as a donor to oxygen atoms in the dATP γ phosphate group, K881 acts as a donor to oxygen atoms of the dATP α and γ phosphates, and N815 acts as an acceptor to dATP O3′) are highly conserved with those in the docked structure of **15** in the EBV BALF5 ternary complex. S587 acts as a donor to an oxygen atom in the γ phosphate group of **15**, L588 acts as a donor to an oxygen atom in the β phosphate group of **15**, R654 acts as a donor to oxygen atoms in the γ phosphate group of **15**, K681 acts as a donor to oxygen atoms of the α and γ phosphate groups of **15**, and N685 acts as a donor to an oxygen atom in the β phosphate group of **15**. In the BALF5/dsDNA/Mg^2+^/**15** model, oxygen atoms in the d-dioxolane ring interact with Y589 and T756, at a distance of approximately 3.6 Å. Both dATP and compound **15** form canonical base-pairing interactions with the deoxythymidine base in the template strand. A conserved DFD motif participates in Mg^2+^ coordination along with oxygen atoms in the α, β, and γ phosphate groups of both the dATP and the docked compound **15** model, positioning the α phosphate group in proximity to the 3′–OH of the primer strand in both cases.

**Figure 5:**
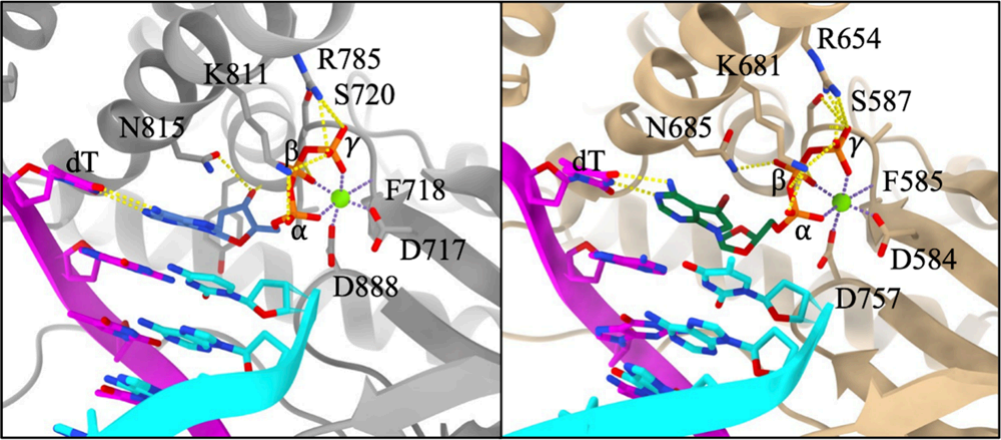
(Left) HSV-1 UL30 in a paused elongation state, depicting the incoming dATP (blue), UL30 (gray). (Right) Homology Model of the polymerase domain of EBV BALF5 (beige) with compound **15** docked in the dNTP binding site. Both panels show the template DNA strand (magenta), the primer DNA strand (cyan), and Mg^2+^ (green). Hydrogen bonding interactions are indicated by yellow dashed lines. Metal coordination interactions are indicated by purple dashed lines. Residue numbering corresponds to that of the respective proteins, and the α, β, and γ phosphate groups are labeled for the sake of clarity.

In conclusion, we described the SAR and antiviral activity of unexplored d-dioxolane-derived 7-deazapurine nucleosides (**12**–**19**) against EBV and HIV. The targeted nucleoside synthesis has been carried out via key ketone (**1**) to furnish critical acylated intermediate **3**. Iodination of **3** followed by S_N_2 coupling was performed to afford the coupled β-nucleosides intermediates, **5**–**11**, which, upon amination and deprotection, yielded target nucleoside analogues **12**–**19**. In-vitro 7-bromo-deazaadenine (**15**) and 7-iodo-deazaadenine (**16**) derivatives exhibited potent anti-EBV activity. Compound **15** displayed a high SI and was 14-fold more potent than the FDA-approved drug GCV. Furthermore, compounds **12** and **13** demonstrated moderate antiviral activity against HIV. This communication suggests that phosphoramidate, phosphate, and phosphonate ester prodrugs of **12**, **13**, **15**, and **16** are warranted for potentially enhancing the antiviral profile of reported nucleosides. Extended SAR of d-dioxolane derived-7-deazapurine analogues may pave a path to new antivirals against EBV and HIV.

## Supplementary Material



## Notes

Experimental procedures for synthesis of intermediates and final compounds, antiviral assay protocols, ^1^H, and ^13^C NMR, spectra of all intermediates and final compounds. HPLC and LCMS methods and trace for compounds **15** and **19** (PDF)

## ABBREVIATIONS

Ac_2_O: acetic anhydride
ACV: acyclovir
ACN: acetonitrile
NH_4_OH: ammonium hydroxide
DMAP: dimethylaminopyridine
DNA: deoxyribonucleic acid
DAA: direct acting antivirals
EBV: Epstein–Barr virus
FCV: famciclovir
GCV: ganciclovir
HIV: human immunodeficiency virus
HSV 1: herpes simplex virus 1
HCV: hepatitis C virus
HMDS: hexamethyldisilazane
KSHV: Kaposi sarcoma-associated herpesvirus
LTBA: lithium tri*tert*-butoxyaluminum hydride
MS: multiple sclerosis
TPA: 12-*O*-tetradecanoylphorbol 13-acetate
PTLD: post-transplant lymphoproliferative disease
PCV: penciclovir
KOH: potassium hydroxide
RNA: ribonucleic acid
SI: selectivity index
NaH: sodium hydride
TMSI: trimethylsilyl iodide
TDA-1: tris­[2-(2-methoxyethoxy)­ethyl]­amine
FDA: The U.S. Food and Drug Administration
VACV: valacyclovir
VZV: varicella-zoster virus
