# Supplementary material for: Synthesis and Antiviral Evaluation of Unexplored Dioxolane-Derived 7‑Deazapurine Nucleoside Analogues against Epstein–Barr Virus (EBV)
Source: ACS Med Chem Lett. 2025 Aug 12;16(9):1844–51. doi: 10.1021/acsmedchemlett.5c00397 (PMC12434534; doi:10.1021/acsmedchemlett.5c00397)
Supplement: Supplementary file 1 [file ml5c00397_si_001.pdf]

Synthesis and Antiviral Evaluation of Unexplored Dioxolane-Derived 7-Deazapurine Nucleoside Analogues against Epstein-Barr Virus (EBV)

Uma S. Singh,[a*] Ransom A. Jones,[a] Yugandhar Kothapalli,[a] Shuiyun Lan,[b] Xing-Quan Zhang,[c] Ryan L. Slack,[b] Harischandra P. Thoomu,[a] Robert T. Schooley,[c] Stefan G. Sarafianos,[b] and Chung K. Chu[a]

[a]Department of Pharmaceutical and Biomedical Sciences, College of Pharmacy, University of Georgia, Athens, Georgia 30602, United States

[b]Center for ViroScience and Cure, Laboratory of Biochemical Pharmacology, Department of Pediatrics, Emory University School of Medicine, Atlanta, Georgia 303322, United States

[c]Department of Medicine, University of California, San Diego, La Jolla, California 92093, United States

Corresponding Author: Uma S. Singh, ussingh@uga.edu

Table of Contents

S. No.	Description of Data	Page number
1.	General Information	S2-S3
2.	Experimental Procedure	S3-S13
3.	Antiviral and cytotoxicity assay protocols	S13-S15
4.	Molecular modeling and compound 15 docking method	S15
5.	^{1}H, ^{13}C NMR, HPLC & LC-MS analysis of compounds **3-19**	S16-S40
6.	X-ray crystallography data of compound 12	S41-S51

1. General Information

Reagents and anhydrous solvents were purchased from commercial sources and used without further purification. Moisture-sensitive reactions were performed using oven-dried glassware under a nitrogen or argon atmosphere. Reactions were monitored by thin-layer chromatography plates (TLC silica gel GF 250 microns) that were visualized using a Spectroline UV lamp (254 nm) and developed with 15% solution of sulfuric acid in methanol. Column chromatography was performed on silica gel 60 Å, 40-63μM (230 X 400 mesh, Sorbent Technologies). Preparative normal phase chromatography was performed on a CombiFlash Rf 150 (Teledyne Isco) with pre-packed RediSep Rf silica gel cartridges or on RediSep® gold C18 reverse phase columns. Melting points were recorded on a Mel-temp II laboratory device and are uncorrected. Nuclear magnetic spectra were recorded on a Varian Inova 500 spectrometer at 500 MHz for ^{1}H NMR, 202 MHz for ^{31}P NMR, 125 MHz for ^{13}C NMR, and 470 MHz for ^{19}F NMR with tetramethylsilane as an internal standard. $CFCl_3$ (trichloro-fluoro methane) was an internal standard reference for ^{19}F-NMR. Chemical shifts (δ) are quoted as s (singlet), bs (broad singlet), d (doublet), t (triplet), q (quartet), m (multiplet), dd (double doublet) and dt (double triplet). Optical rotations were measured on a JASCO DIP-370 digital polarimeter. High-resolution mass spectroscopy (HRMS) spectra were measured on Bruker Ultra-high resolution QTOF MS Impact II spectrometer. Samples were infused at 3 μL/min, and spectra were obtained in the positive or negative ionization mode with a typical resolution of 20,000 or greater. The purity of final compounds was determined by HPLC/UV. All tested compounds are >95% pure by HPLC. HPLC/UV was determined with a Waters HPLC coupled with a photodiode array. 5 μL of sample 0.5 mg/mL in methanol, or in acetonitrile or in a mixture of $MeOH/H_2O$ (0.5:10) were injected, using an XBrigde C18, 3.5 μm, (4.6 X 150) mm column at 25 °C with a flow rate 0.8 mL/min or

with UPLC BEH C18, 1.7 μm (100 X 2.1) mm at 50 °C with a flow rate 0.55 mL/min . The mobile phases were a mixture of A = 10 mM ammonium bicarbonate in water and B = acetonitrile (ACN). Purity is given as % of absorbance at Max plot.

2. Experimental Procedure

Synthetic Protocol and Analytical Data:

((2*R*)-4-acetoxy-1,3-dioxolan-2-yl)methyl isobutyrate (3).

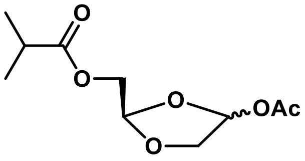

To a stirred solution of **1** (11.0 g, 58.45 mmol) in THF (50 mL) under N_2 cooled to -78 °C, Lithium tri-*tert*-butoxyaluminum hydride (LTBA) (72 mL, 1M in THF) was added dropwise over 1 hour at -10 °C. After that, the mixture was stirred for an additional 2 hours at -10 °C to afford intermediate **2**. DMAP (9.0 g, 73.07 mmol) was added, and the mixture was stirred for 30 minutes; after that, acetic anhydride (28 mL, 292.27 mmol) was added dropwise. The mixture was stirred for 16 h at room temperature and quenched with saturated NH_4Cl (20 mL). A murky suspension was obtained and filtered through a celite bed to yield a clear biphasic mixture. The organic layer was separated and concentrated under reduced pressure to give a brown residue, which was dissolved in EtOAc (500 mL). The organic layer was washed with water (30 mL x 2), brine (30 mL x 2), and finally with a saturated aqueous solution of $NaHCO_3$ (aq. 30 mL) and then concentrated under reduced pressure. The crude was purified via column chromatography (6% EtOAc/Hexanes) gave inseparable diastereomeric mixture **3** as a yellow oil. Yield: (5.2 g, 39%); ^{1}H NMR (500 MHz, $CDCl_3$) (mix of diastereomers) δ 6.34-6.28 (m, 1H), 5.35, 5.25 (t, J = 4.0 Hz, 1H), 4.33-3.90 (m, 4H), 2.58-2.51 (m, 1H), 2.04-2.02 (m, 3H), 1.13-1.11 (m, 6H); ^{13}C NMR (125

MHz, $CDCl_3$) δ 176.5, 176.5, 170.2, 170.1, 103.6, 102.6, 102.3, 94.5, 94.0, 71.3, 70.8, 64.1, 63.5, 63.4, 63.2, 33.8, 21.1, 21.0, 18.9, 18.8; HRMS (ESI-TOF) m/z: $[M + Na]^+$ Calcd for $[C_{10}H_{16}O_6Na]^+$ 255.0839; found 255.0836.

Synthesis of the target compounds 5 – 11:

((2*R*,4*R*)-4-(4-chloro-7*H*-pyrrolo[2,3-*d*]pyrimidin-7-yl)-1,3-dioxolan-2-yl)methylisobutyrate (5).

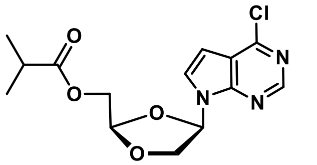

To a stirred solution of **3** (1.0 g, 4.31 mmol) in anhydrous $CHCl_3$ (36 mL) under N_2 cooled to -20 °C, iodotrimethylsilane (TMSI, 0.75 mL, 5.17 mmol) was added. The mixture was stirred at -20 °C for 3 hours to afford intermediate **4**. In a separate round bottom flask, KOH powder (725 mg, 12.93 mmol) was taken under N_2 in anhydrous ACN (25 mL) and stirred for 30 minutes. After that, 0.1 mL of tris[2-(2-methoxyethoxy)ethyl]amine (TDA-1) was added, and the mixture was stirred vigorously for 30 minutes, until the clumps of KOH disappeared. To this mixture, 6-chloro-7-deazapurine (725 mg, 4.31 mmol) was added, and the reaction was vigorously stirred for an additional 20 minutes. The mixture was cooled to 0 °C, and the crude **4** was added slowly over 5 minutes and stirred for 1 hour. The murky suspension was passed through a medium frit, quenched with aq. $Na_2S_2O_3$ (25 mL), was washed with water (2 x 50 mL), dried over Na_2SO_4, and concentrated under reduced pressure. The crude residue was purified *via* column chromatography (11% EtOAc/Hexanes) to give **5** as a white crystalline powder. Yield: (200 mg, 14%); m. p. 42-48 °C; ^1H NMR (500 MHz, $CDCl_3$) δ 8.63 (s, 1H), 7.58 (d, *J* = 3.5Hz, 1H), 6.80 (d, *J* = 5.5 Hz, 1H), 6.67 (d, *J* = 4.0 Hz, 1H), 5.29 (t, *J* = 3.5 Hz, 1H), 4.45-4.28 (m, 4H), 2.62-2.54 (m, 1H), 1.16 (dd,

$J = 7.0$ & 19.5 Hz, 6H); ^{13}C NMR (125 MHz, $CDCl_3$) δ 176.6, 152.4, 151.4, 151.2, 125.9, 117.9, 103.1, 101.5, 79.6, 71.3, 63.0, 33.9, 19.0, 19.0; HRMS (ESI-TOF) m/z: $[M + Na]^+$ Calcd for $[C_{14}H_{16}ClN_3O_4Na]^+$ 348.0722; found 348.0722.

((2*R*,4*R*)-4-(2-amino-4-chloro-7*H*-pyrrolo[2,3-*d*]pyrimidin-7-yl)-1,3-dioxolan-2-yl)methylisobutyrate (6).

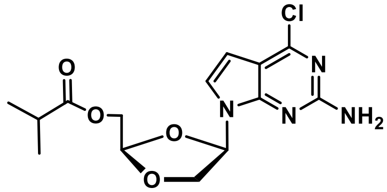

Compound **6** was synthesized according to the above procedure described for the synthesis of **5**. Yield: (200 mg, white solid, 14%); m. p. 56-58 °C; ^{1}H NMR (500 MHz, $CDCl_3$) δ 7.12 (d, $J = 4.0$ Hz, 1H), 6.54-6.53 (m, 1H), 6.40 (d, $J = 3.5$ Hz, 1H), 5.30 (bs, 2H), 5.22 (t, $J = 4.0$ Hz, 1H), 4.37-4.28 (m, 2H), 4.25-4.17 (m, 2H), 2.58-2.52 (m, 1H), 1.13 (dd, $J = 7.0$ & 18.0 Hz, 6H); ^{13}C NMR (125 MHz, $CDCl_3$) δ 207.0, 176.6, 159.0, 154.0, 152.8, 122.1, 110.6, 102.8, 79.2, 70.7, 63.2, 33.8, 19.0, 18.9; HRMS (ESI-TOF) m/z: $[M + Na]^+$ Calcd for $[C_{14}H_{17}ClN_4O_4Na]^+$ 363.0831; found 363.0826.

((2*R*,4*R*)-4-(4-chloro-2-fluoro-7*H*-pyrrolo[2,3-*d*]pyrimidin-7-yl)-1,3-dioxolan-2-yl)methylisobutyrate (7).

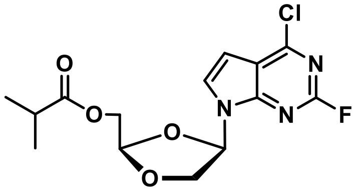

Compound **7** was synthesized according to the above procedure described for the synthesis of **5**. Yield: (450 mg, white powder, 30%); m. p. 37-40 °C; ^{1}H NMR (500 MHz, $CDCl_3$) δ 8.60 (s, 1H), 7.35 (d, $J = 3.0$ Hz, 1H), 6.84 (d, $J = 6.5$ Hz, 1H), 5.24 (t, $J = 3.5$ Hz, 1H), 4.38 (dd, $J = 1.5$ Hz & 10.0 Hz, 1H), 4.35-4.28 (m, 2H), 4.26 (dd, $J = 4.5$ & 10.0 Hz, 1H), 2.62-2.54 (m, 1H), 1.16 (dd, $J = 7.0$ & 21.5 Hz, 6H); ^{13}C NMR (125 MHz, $CDCl_3$) δ 176.6, 158.6, 156.8, 126.3, 116.3, 103.2,

102.0, 101.2, 79.7, 71.3, 63.0, 33.9, 19.0; ^{19}F NMR (470 MHz, $CDCl_3$) δ -51.64 (s, 1F); HRMS (ESI-TOF) m/z: [M + Na]+ Calcd for $[C_{14}H_{15}ClFN_3O_4Na]^+$ 366.0627; found 366.0617.

((2*R*,4*R*)-4-(4-chloro-5-fluoro-7*H*-pyrrolo[2,3-*d*]pyrimidin-7-yl)-1,3-dioxolan-2-yl)methylisobutyrate (8).

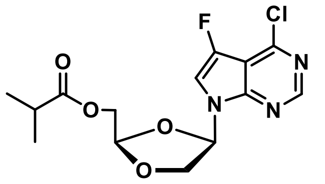

Compound **8** was synthesized according to the above procedure described for the synthesis of **5**. Yield: (210 mg, pale yellow solid, 14%); m. p.74-76 °C; ^{1}H NMR (500 MHz, $CDCl_3$) δ 8.60 (s, 1H), 7.35 (d, *J* = 2.5 Hz, 1H), 6.84 (d, *J* = 5.5 Hz, 1H), 5.24 (t, *J* = 3.3 Hz, 1H), 4.38 (dd, *J* = 10.0 & 1.3 Hz, 1H), 4.31 (dd, *J* = 3.2 & 2.3 Hz, 2H), 4.26 (dd, *J* = 10.0 & 5.5 Hz, 1H), 2.57 (h, *J* = 7.0 Hz, 1H), 1.18 (d, *J* = 7.0 Hz, 3H), 1.14 (d, *J* = 7.0 Hz, 3H); ^{13}C NMR (125 MHz, $CDCl_3$) δ 176.6, 151.9, 150.9, 147.2, 143.1, 141.1, 108.7, 107.4, 103.1, 79.2, 71.2, 62.7, 33.9, 18.9; ^{19}F NMR (470 MHz, $CDCl_3$) δ -166.17 (t, *J* = 2.35Hz, 1F); HRMS (ESI-TOF) m/z: $[M + H]^+$ Calcd for $[C_{14}H_{16}ClFN_3O_4]^+$ 344.0808; found 344.0805.

((2*R*,4*R*)-4-(4,5-dichloro-7*H*-pyrrolo[2,3-*d*]pyrimidin-7-yl)-1,3-dioxolan-2-yl)methyl isobutyrate (9)

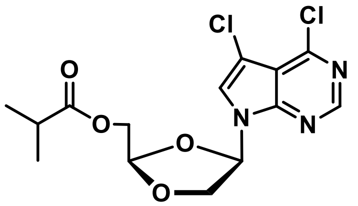

Compound **9** was synthesized according to the above procedure described for the synthesis of **5**. Yield: (270 mg, off white powder, 17%); m. p. 102-105 °C; ^{1}H NMR (500 MHz, CD_3OD) δ 8.58 (s, 1H), 7.81 (s, 1H), 6.78 (d, *J* = 4.6 Hz, 1H), 5.27 (t, *J* = 2.6 Hz, 1H), 4.52 (d, *J* = 10.2 Hz, 1H),

4.35-4.27 (m, 3H), 2.55 (p, *J* = 7.0 Hz, 1H), 1.14 (d, *J* = 7.0 Hz, 3H), 1.04 (d, *J* = 7.0 Hz, 3H); ^{13}C NMR (125 MHz, CD_3OD) δ 176.7, 151.2, 150.8, 145.8, 124.7, 113.6, 104.8, 103.1, 79.8, 70.8, 61.9, 33.7, 18.0, 17.9; HRMS-ESI (*m/z*): [M + H]$^+$ calculated for $[C_{14}H_{16}Cl_2N_3O_4]^+$ 360.0512; found 360.0513.

((2*R*,4*R*)-4-(5-bromo-4-chloro-7*H*-pyrrolo[2,3-*d*]pyrimidin-7-yl)-1,3-dioxolan-2-yl)methyl isobutyrate (10).

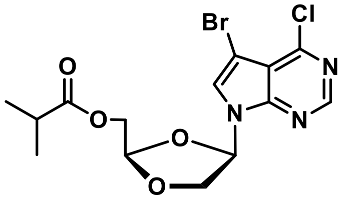

Compound **10** was synthesized according to the above procedure described for the synthesis of **5**. Yield: (232 mg, off white powder, 13%); m. p. 108-112 °C; ^{1}H NMR (500 MHz, $CDCl_3$) δ 8.61 (s, 1H), 7.69 (s, 1H), 6.79 (d, *J* = 4.8 Hz, 1H), 5.26 (s, 1H), 4.42-4.25 (m, 4H), 2.62 (p, *J* = 6.8 Hz, 1H), 1.20 (d, *J* = 6.9 Hz, 3H), 1.14 (d, *J* = 6.9 Hz, 3H); ^{13}C NMR (125 MHz, $CDCl_3$) δ 176.7, 152.6, 151.6, 150.6, 126.1, 115.2, 103.3, 89.9, 79.5, 71.6, 62.3, 33.9, 19.2, 19.1; HRMS (ESI-TOF) m/z: [M + Na]$^+$ Calcd for $[C_{14}H_{15}BrClN_3O_4Na]^+$ 425.9827; found 425.9814.

((2*R*,4*R*)-4-(4-chloro-5-iodo-7*H*-pyrrolo[2,3-*d*]pyrimidin-7-yl)-1,3-dioxolan-2-yl)methyl isobutyrate (11).

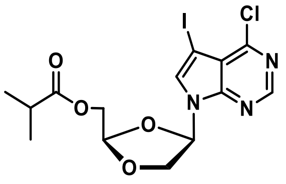

Compound **11** was synthesized according to the above procedure described for the synthesis of **5**. Yield: (160 mg, white powder, 8%); m. p. 142-146 °C; ^{1}H NMR (500 MHz, $CDCl_3$) δ 8.57 (s, 1H), 7.78 (s, 1H), 6.75 (s, 1H), 5.25 (s, 1H), 4.43-4.21 (m, 4H), 2.63 (dq, *J* = 14.0, 7.0 Hz, 1H), 1.28

(d, J = 6.9 Hz, 3H), 1.12 (t d, J = 6.9 Hz, 3H); ^{13}C NMR (125 MHz, $CDCl_3$) δ 176.8, 152.9, 151.1, 150.8, 131.7, 117.1, 103.2, 79.60, 71.6, 62.2, 53.4, 33.9, 19.2, 19.1; HRMS (ESI-TOF) m/z: [M + Na]$^+$ Calcd for $[C_{14}H_{15}ClIN_3O_4Na]^+$ 473.9688; found 473.9669.

Synthesis of the target compounds 12 – 19:

((2*R*,4*R*)-4-(4-amino-7*H*-pyrrolo[2,3-*d*]pyrimidin-7-yl)-1,3-dioxolan-2-yl)methanol (12).

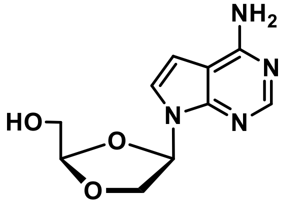

In a sealed tube **5** (180 mg, 0.55 mmol) was taken in dioxane (2 mL) and NH_4OH (4 mL) were added. The solution was allowed to stir under higher pressure for 24 hours at 100 °C. After that, the solution was allowed to cool to room temperature, and the solvent was evaporated *in vacuo*. The residue was purified *via* column chromatography (5% MeOH/DCM) and trituration with ether/hexane to give **12** as an off-white fluffy solid. Yield: (50 mg, 38%); m. p.: 138-148 °C; $[\alpha]^{26}_D$ = -47.03 (c 0.5, MeOH); ^{1}H NMR (500 MHz, CD_3OD) δ 8.06 (s, 1H), 7.38 (d, J = 3.5Hz, 1H), 6.59-6.57 (m, 2H), 5.08 (t, J = 3.0Hz, 1H), 4.36-4.33 (m, 1H), 4.25-4.22 (m, 1H), 3.72 (d, J = 6.0Hz, 2H); ^{13}C NMR (125 MHz, CD_3OD) δ 157.6, 151.0, 149.6, 121.5, 105.2, 103.0, 100.2, 79.7, 70.8, 61.8; HRMS (ESI-TOF) m/z: [M + Na]$^+$ Calcd for $[C_{10}H_{12}N_4O_3Na]^+$ 259.0802; found 259.0801.

((2*R*,4*R*)-4-(4-amino-5-fluoro-7*H*-pyrrolo[2,3-*d*]pyrimidin-7-yl)-1,3-dioxolan-2-yl)methanol (13).

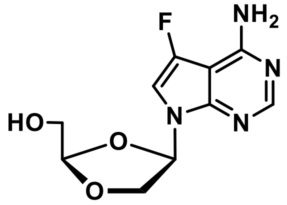

In a sealed tube **8** (200 mg, 0.58 mmol) was taken in dioxane (2 mL) and NH_4OH (4 mL) were added. The solution was allowed to stir under higher pressure at 100 °C for 24h. After that, the solution was allowed to cool to room temperature, and the solvent was evaporated *in vacuo* to give a crude yellow solid. The solid was dissolved in 80 °C water (3 mL) and stirred until full dissolution was observed. Purification via cooling recrystallization afforded **13** as an off-white beige powder. Yield: (116 mg, 79%); m. p. 178-180 °C; $[\alpha]^{25}_{D}$ = -77.52 (c 0.5 in MeOH); ^{1}H NMR (500 MHz, CD_3OD) δ 8.06 (s, 1H), 7.21 (d, *J* = 1.5 Hz, 1H), 6.63 (d, *J* = 5.5 Hz, 1H), 5.04 (t, *J* = 2.5 Hz, 1H), 4.31 (d, *J* = 5.5 Hz, 1H), 4.22-4.19 (m, 1H), 3.72 (d, *J* = 2.5 Hz, 2H); ^{13}C NMR (125 MHz, CD_3OD) δ 152.4, 145.8, 144.9, 142.9, 105.2, 103.9, 103.7, 79.3, 70.7, 61.5; ^{19}F NMR (470 MHz, CD_3OD) δ -169.37 (s, 1F); HRMS (ESI-TOF) m/z: $[M + H]^+$ Calcd for $[C_{10}H_{12}FN_4O_3]^+$ 255.0888; found 255.0883.

((2*R*,4*R*)-4-(4-amino-5-chloro-7*H*-pyrrolo[2,3-*d*]pyrimidin-7-yl)-1,3-dioxolan-2-yl)methanol (14).

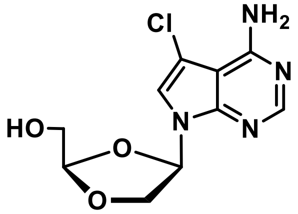

In a sealed tube, **9** (200 mg, 0.58 mmol) was taken in dioxane (2 mL), and NH_4OH (4 mL) was added. The solution was allowed to stir under higher pressure at 100 °C for 24h. After that, the solution was allowed to cool to room temperature, and the solvent was evaporated *in vacuo* to give a crude yellow solid. The solid was dissolved in 80 °C water (3 mL) and stirred until full dissolution was observed. Purification via cooling recrystallization afforded **14** as an off-white beige powder. Yield: (109 mg, 72%); m. p. 179-182 °C; ^{1}H NMR (500 MHz, CD_3OD) δ 8.08 (s, 1H), 7.47 (s, 1H), 6.60 (d, *J* = 4.9 Hz, 1H), 5.06 (s, 1H), 4.33 (d, *J* = 9.5 Hz, 1H), 4.22 (dd, *J* =

10.4, 5.7 Hz, 1H), 3.73 (s, 2H); ^{13}C NMR (125 MHz, CD_3OD) δ 157.2, 152.3, 152.2, 148.8, 118.8, 105.3, 104.5, 79.6, 70.9, 61.4; HRMS-ESI (*m/z*): [M + Na]$^+$ calculated for $[C_{10}H_{11}ClN_4O_3Na]^+$ 293.0412; found 293.0399.

((2*R*,4*R*)-4-(4-amino-5-bromo-7*H*-pyrrolo[2,3-*d*]pyrimidin-7-yl)-1,3-dioxolan-2-yl)methyl isobutyrate (15).

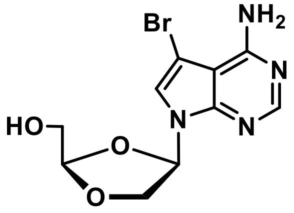

In a sealed tube **10** (110 mg, 0.27 mmol) was taken in dioxane (1 mL) and NH_4OH (2 mL) were added. The solution was allowed to stir under higher pressure at 100 °C for 24h. After that, the solution was allowed to cool to room temperature, and the solvent was evaporated *in vacuo* to give a crude yellow solid. The solid was dissolved in 80 °C water (3 mL) and stirred until complete dissolution was observed. Purification via cooling recrystallization afforded **15** as a light-yellow powder. Yield: (35 mg, 33%); m. p. 184-186 °C; $[\alpha]^{25}_D$ = -11.64 (c 0.25 in MeOH); ^{1}H NMR (500 MHz, DMSO-*d$_6$*): δ 8.08 (s, 1H), 7.48 (s, 1H), 6.76 (brs, 2H) 6.54 (s, 1H), 5.09 (s, 1H), 4.96 (s, 1H), 4.30 (d, *J* = 8.0 Hz, 1H), 4.15 (s, 1H), 3.56 (s, 2H); ^{13}C NMR (125 MHz, DMSO-*d$_6$*) δ 157.5, 153.2, 149.9, 121.4, 105.7, 101.1, 87.8, 79.3, 71.1, 61.8; HRMS (ESI-TOF) m/z: [M + H]$^+$ Calcd for $[C_{10}H_{12}N_4O_3Br]^+$ 315.0087; found 315.0072.

((2*R*,4*R*)-4-(4-amino-5-iodo-7*H*-pyrrolo[2,3-*d*]pyrimidin-7-yl)-1,3-dioxolan-2-yl)methyl isobutyrate (16).

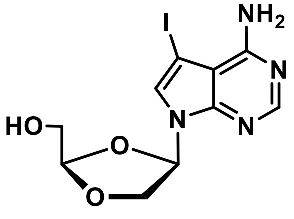

In a sealed tube **11** (98 mg, 0.21 mmol) was taken in dioxane (1 mL) and NH_4OH (2 mL) were added. The solution was allowed to stir under higher pressure at 100 °C for 24h. After that, the solution was allowed to cool to room temperature, and the solvent was evaporated *in vacuo* to give a crude yellow solid. The solid was dissolved in 80 °C water (3 mL) and stirred until full dissolution was observed. The mixture was stored in the freezer for 16 h, the obtained crystals were filtered and dried to afford **16** as a white powder. Yield: (17 mg, 18%); m. p. 222-223 °C; ^{1}H NMR (500 MHz, DMSO-d_6): δ 8.08 (s, 1H), 7.52 (s, 1H), 6.65 (brs, 2H), 6.52 (s, 1H), 5.09 (s, 1H), 4.96 (s, 1H), 4.29 (d, J = 8.6 Hz, 1H), 4.14 (s, 1H), 3.55 (s, 2H); ^{13}C NMR (125 MHz, DMSO-d_6): δ 157.7, 152.7, 150.4, 126.7, 105.7, 103.3, 79.3, 71.0, 61.8, 53.0; HRMS (ESI-TOF) m/z: $[M + Na]^+$ Calcd for $[C_{10}H_{11}IN_4O_3Na]^+$:384.9768; found 384.9769.

((2*R*,4*R*)-4-(2,4-diamino-7*H*-pyrrolo[2,3-*d*]pyrimidin-7-yl)-1,3-dioxolan-2-yl)methanol (17).

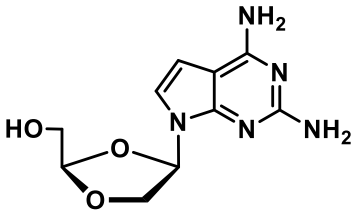

In a sealed tube **6** (200 mg, 0.59 mmol) was taken in dioxane (2 mL) and NH_4OH (4 mL) were added. The solution was allowed to stir under higher pressure for 72 hours at 135 °C. After that, the solution was allowed to cool to room temperature, and the solvent was evaporated *in vacuo*. Purification with a preparatory TLC (10% MeOH/DCM) gave **17** as a yellow powder. Yield: (27 mg, 18%); m. p. 140-145 °C; $[\alpha]^{25}_{D}$ = -73.61 (c 0.5 in MeOH);); ^{1}H NMR (500 MHz, CD_3OD) δ 6.98 (d, J = 4.0 Hz, 1H), 6.40-6.39 (m, 2H), 5.05 (t, J = 3.5 Hz, 1H), 4.29 (d, J = 2.0 Hz & 9.5 Hz, 1H), 4.19-4.16 (m, 1H), 3.70 (d, J = 3.5 Hz, 2H); ^{13}C NMR (125 MHz, CD_3OD) δ 159.5, 158.1, 152.2, 118.1, 104.9, 100.5, 96.5, 79.6, 70.4, 62.1; HRMS (ESI-TOF) m/z: $[M + H]^+$ Calcd for $[C_{10}H_{14}N_5O_3]^+$ 252.1091; found 252.1091.

((2*R*,4*R*)-4-(4-amino-2-fluoro-7*H*-pyrrolo[2,3-*d*]pyrimidin-7-yl)-1,3-dioxolan-2-yl)methanol (18).

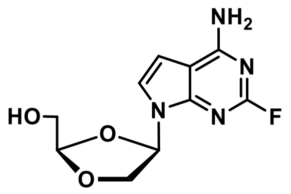

In a sealed tube **7** (100 mg, 0.29 mmol) was taken in dioxane (2 mL) and NH_4OH (4 mL) were added. The solution was stirred under higher pressure for 24 hours at 100 °C. After that, the solution was allowed to cool to room temperature, and the solvent was evaporated *in vacuo* to give a crude yellow solid. The solid was dissolved in 80 °C isopropanol (3 mL) and stirred until complete dissolution was observed. Purification via antisolvent recrystallization with hexane afforded **18** as a white powder. Yield: (35 mg, 47%); m. p.160-165 °C; $[\alpha]^{27}_D$ = -63.10 (c 0.5 in MeOH); ^{1}H NMR (500 MHz, CD_3OD) δ 7.14 (d, *J* = 3.5 Hz, 1H), 6.52 (d, *J* = 3.5 Hz, 1H), 6.42 (d, *J* = 5.5 Hz, 1H), 5.05 (s, 1H), 4.32-4.18 (m, 2H), 3.72 (d, *J* = 3.0 Hz, 2H); ^{13}C NMR (125 MHz, CD_3OD) δ 183.2, 153.8, 120.3, 105.2, 101.2, 100.0, 95.2, 79.8, 70.6, 61.6; ^{19}F NMR (470 MHz, CD_3OD) δ -129.05 (s, 1F); HRMS (ESI-TOF) m/z: [M - H]$^-$ Calcd for $[C_{10}H_{10}FN_4O_3]^-$ 253.0742; found 253.0746.

((2*R*,4*R*)-4-(2-amino-4-(methylamino)-7*H*-pyrrolo[2,3-*d*]pyrimidin-7-yl)-1,3-dioxolan-2-yl)methanol (19).

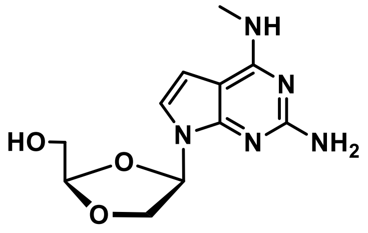

In a sealed tube **6** (100 mg, 0.29 mmol) was taken in methylamine in ethanol (33%/wt, 8 mL). The solution was allowed to stir under higher pressure for 24 hours at 160 °C. After that, the solution was allowed to cool to room temperature, and the solvent was evaporated *in vacuo*. The crude residue was purified via preparatory TLC (7.5% MeOH/DCM) to afford **19** as an off-white powder. Yield: (32 mg, 40%); m. p.145-155 °C; $[\alpha]^{25}_{D}$ = -91.64 (c 0.5 in MeOH); ^{1}H NMR (500 MHz, CD_3OD) δ 6.93 (d, *J* = 4.0 Hz, 1H), 6.39-6.37 (m, 1H), 6.35 (d, *J* = 3.5 Hz, 1H), 5.05 (t, *J* = 3.5 Hz, 1H), 4.28 (dd, *J* = 2.0 & 9.5 Hz, 1H), 4.18-4.15 (m, 1H), 3.69 (d, *J* = 3.0 Hz, 2H), 2.96 (s, 3H); ^{13}C NMR (125 MHz, CD_3OD) δ 160.1, 158.1, 117.3, 104.9, 100.1, 100.0, 96.9, 79.6, 70.4, 62.2, 26.5; HRMS (ESI-TOF) m/z: $[M + H]^+$ Calcd for $[C_{11}H_{16}N_5O_3]^+$ 266.1248; found 266.1243.

3. Antiviral and cytotoxicity assay protocols

Compounds and formulations: All tested compounds were prepared as 10 mM stocks in DMSO and stored at -80 °C. The control compounds, ganciclovir (Sigma-Aldrich) and **PF-74 (Millipore Sigma, Burlington, MA)**, are commercially available. Stock compounds were diluted in DMSO and/or complete tissue culture media prior to being added to cells.

Cells lines: P3HR-1 cells (ATCC HTB-62) were purchased from The American Types Culture Collection (ATCC) and grown in RPMI 1640 with 10% heat-inactivated fetal bovine serum (FBS) and 100 IU/mL penicillin and 100 µL/ mL streptomycin. TZM-GFP cells were purchased from the ATCC and grown in RPMI 1640 with 10% heat-inactivated fetal bovine serum (FBS) and 100 IU/mL penicillin and 100 µL/mL streptomycin

***In vitro* antiviral susceptibility assay:** a) For EBV assay, 24- well plated seeded with $1x10^6$ cells per well in 1 mL of medium. Cells were induced with 25 ng/mL 12-*O*-tetradecanoylphorbol 13-acetate (TPA, Sigma-Aldrich), and incubated with various concentrations of compounds (25, 5, 1,

0.2, 0.04, 0 μM). TPA was removed after 24 h, and the same concentration of compounds was replaced. Cells were harvested on day 5 by removing a 200 μL aliquot of resuspended cells and medium for extraction. b) For HIV assay, TZM-GFP cells were seeded in the 96-well plate (10,000/well), and after 24 h, selected compounds and HIV FL (MOI=0.1) virus were added to the plate. Reference compound PF-74 was used as a positive control. At 48 h post-infection (hpi), the GFP cells were measured by Cytation 5, and a 50% reduction in efficacy (EC_{50}) and cell viability (CC_{50}) was determined using GraphPad software (San Diego, California, www.graphpad.com).

Real-time PCR detection of viral DNA: EBV DNA was extracted with a QIAGEN Dneasy Blood and Tissue Kit. The DNA volume was 30 μL. DNA concentration (ng/μL) was determined with a Nanophotometer (IMPLEN). The Real-time PCR was performed using QuantiTect Sybr Green PCR Kit. The amplicon was a 71 bp portion of the EBNA1 gene. The primers were purchased from IDT. The forward primer was 5‘-GAC TGT GTG CAG CTT TGA CGA T-3’; the reverse primer was 5’ -CGG CAG CCC CTT CCA-3’. The 20 μL PCR sample contained 100 ng DNA, 10 μL Universal PCR Master Mix, primer (0.5 uM final concentration), 1 μL BamHI-HF (Restriction Enzyme, BioLabs) and RNase-free water. The PCR program consisted of 1 cycle at 50 °C, 2 minutes of UNG pretreatment, followed by 1 cycle at 95 °C for 15 minutes. Then 40 cycles of 94 °C for 15 seconds, 55 °C for 30 seconds, and 72 °C for 30 seconds were carried out.

Evaluation of Cytotoxicity: The cytotoxic effects of test compounds for P3HR1 and TZN-GFP cells were determined by CellTiter 96 Non-Radioactive Cell Proliferation assay system (Promega) using either 2,3-Bis-(2-Methoxy-4-nitro-5-sulfophenyl)-2H-tetrazolium-5-carboxanilide, disodium salt (XTT) or 3- (4,5-dimethylthiazol-2-yl)-2,5-diphenyltetrazolium bromide (MTT) methods under the same conditions as the antiviral assay but in the absence virus. Cells were seeded at a density of 1 x 10^5 (P3HR-1) or 2 x 10^4 (TZM-GFP) cells per well in 96-well plates and

allowed to incubate overnight. On the second day, different concentrations of test compounds were added to respective cells. Following 4 days of incubation at 37 °C in a CO_2 incubator, the MTT or XTT reagent was added, and the cells were incubated for 3 hours at 37 °C. Subsequently, the absorbance of the samples was measured using a microplate reader (Biotek). The cytotoxic concentration (CC_{50}) was determined based on the viability of mock-infected cells.

4. Molecular Modeling and Compound 15 Docking Method:

An experimental structure of the EBV DNA polymerase encoded by the BALF5 gene has not been reported. Therefore, we constructed a homology model to examine interactions that may contribute to the antiviral activity of these compounds. The sequence for the DNA-directed DNA polymerase domain (residues 523-994, UniProtKB Accession ‘A0A0C7T056’)[1] of BALF5 served as the reference amino acid sequence for the homology model construction. Furthermore, multiple sequence alignment using Schrödinger Maestro (Schrödinger, NY, 2025) was performed. Then it was determined that the HSV-1 DNA polymerase-processivity factor complex (PDB: 8OJ6, chain A)[2] was the closest homolog (45% sequence identity) with a high-quality experimental structure, which we used as the structural template for modeling. Homology modeling utilized the knowledge-based model-building method in Prime within the Schrödinger suite 2025-2 (Schrödinger, NY, United States). Portions of the double-stranded DNA and a magnesium ion from the template structure were included during the model-building process, and an incoming dATP was manually added to the final model before energy minimization in Schrödinger Maestro. Docking of compound **15** at the incoming dATP site was conducted using the Glide feature in the Schrödinger suite 2025-2.[3]

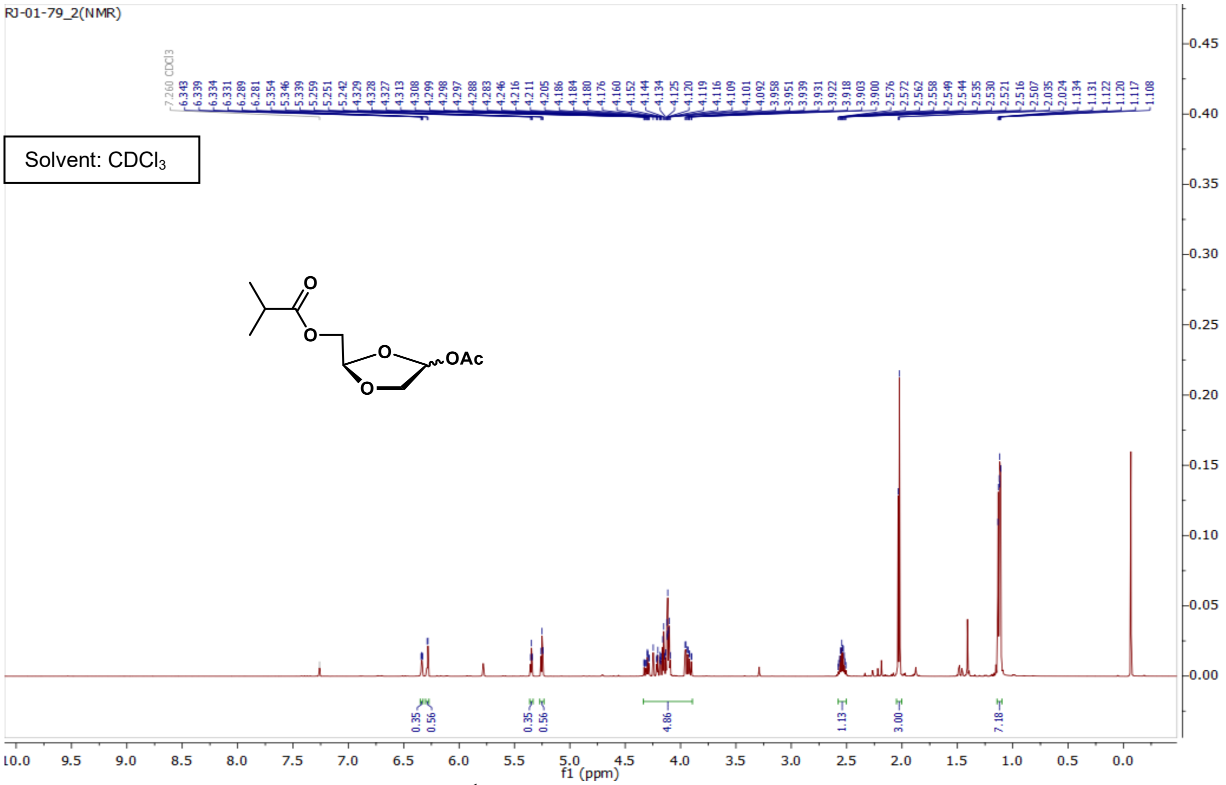

^{1}H NMR of compound **3**

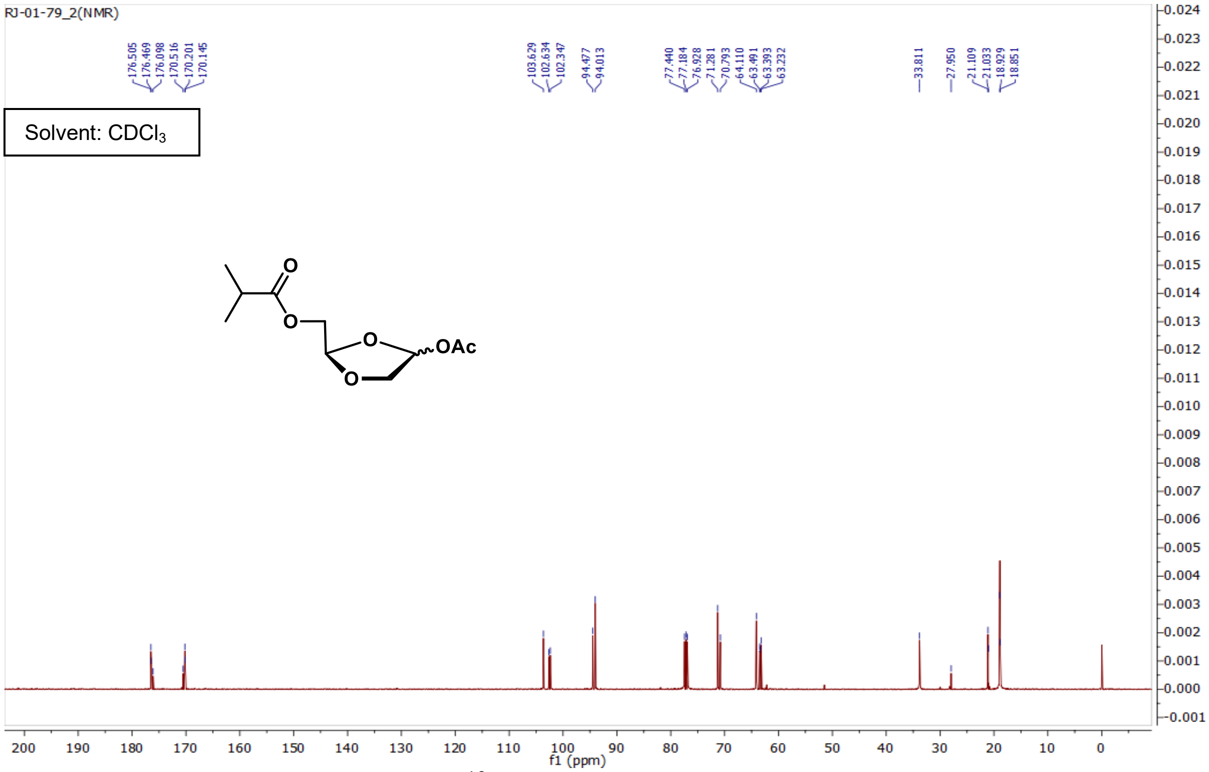

^{13}C NMR of compound **3**

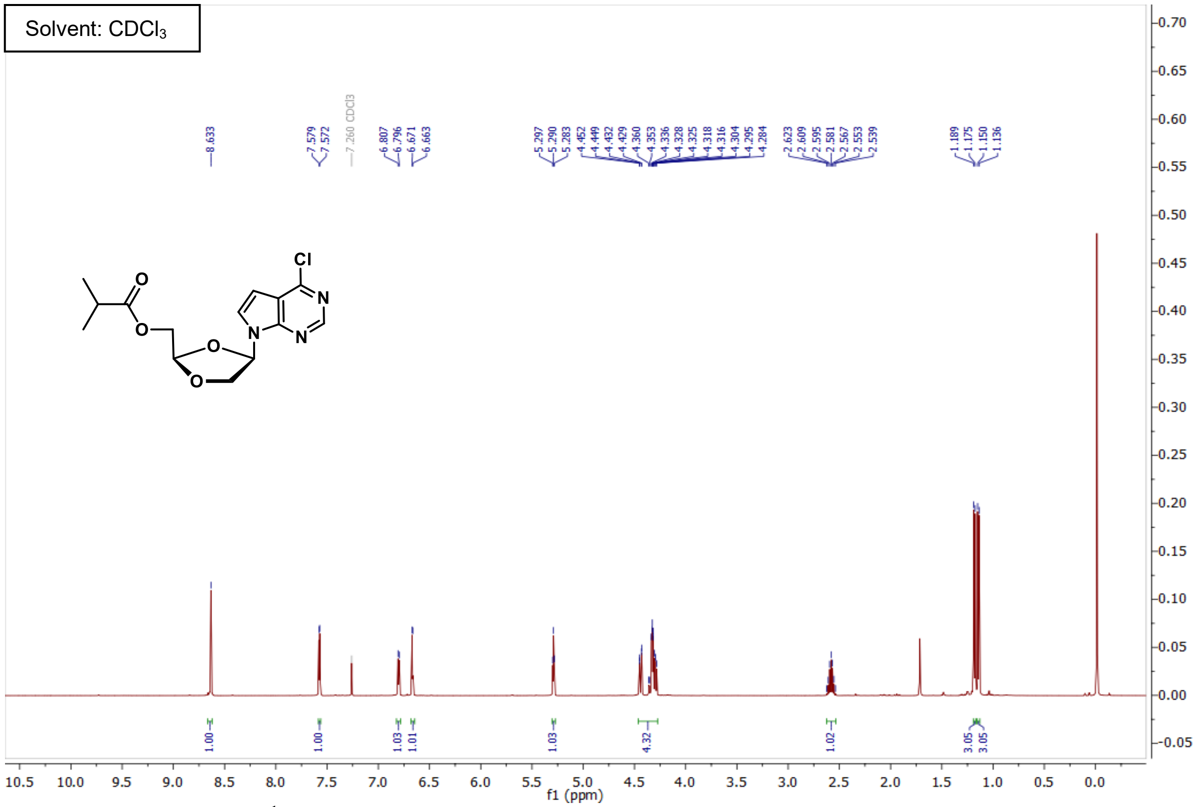

^{1}H NMR of compound **5** (desired *β*-deazapurine analog)

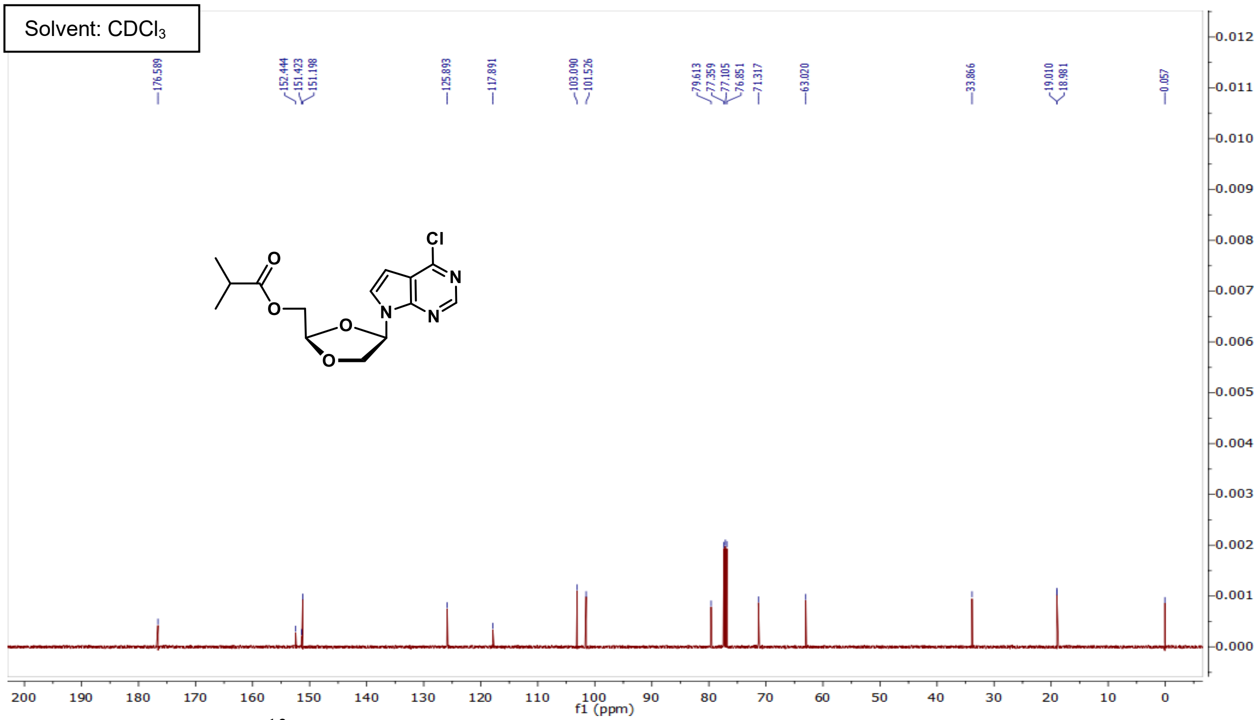

^{13}C NMR of compound **5** (desired *β*-deazapurine analog)

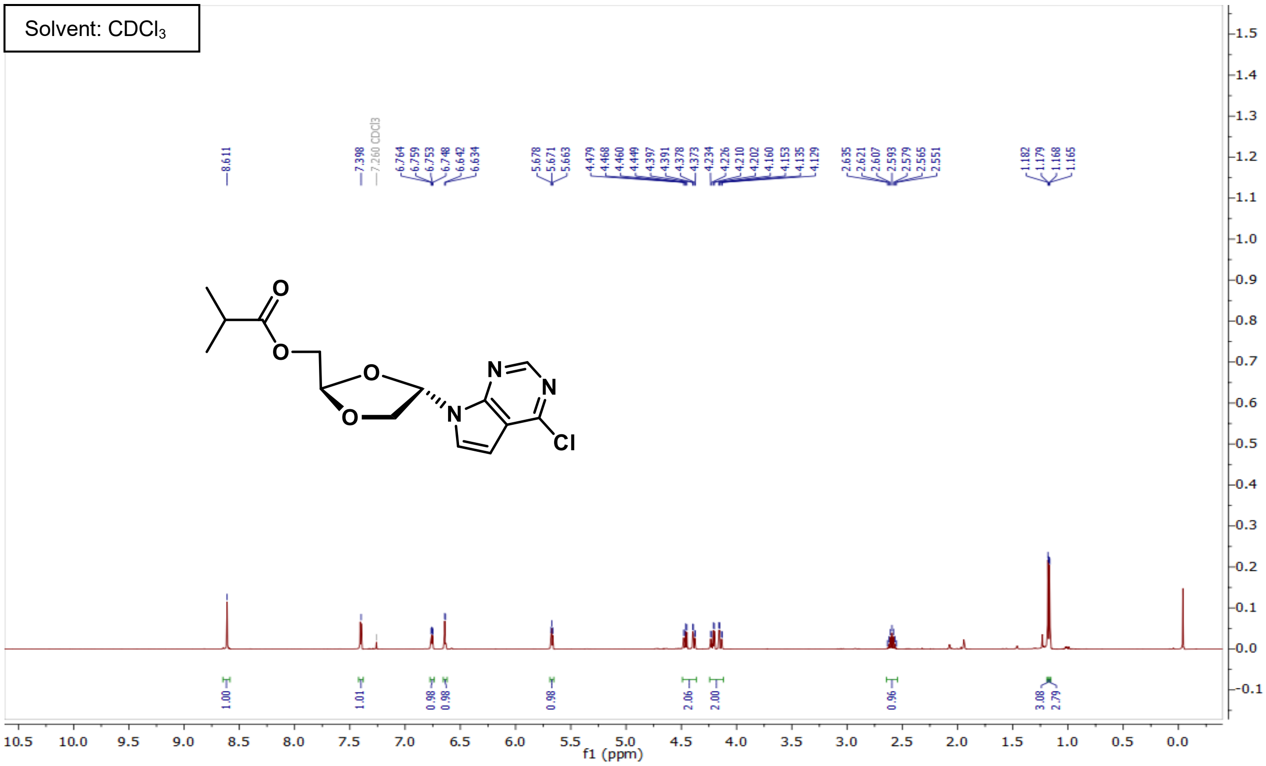

^{1}H NMR of compound **5a** (undesired *α*-deazapurine analog)

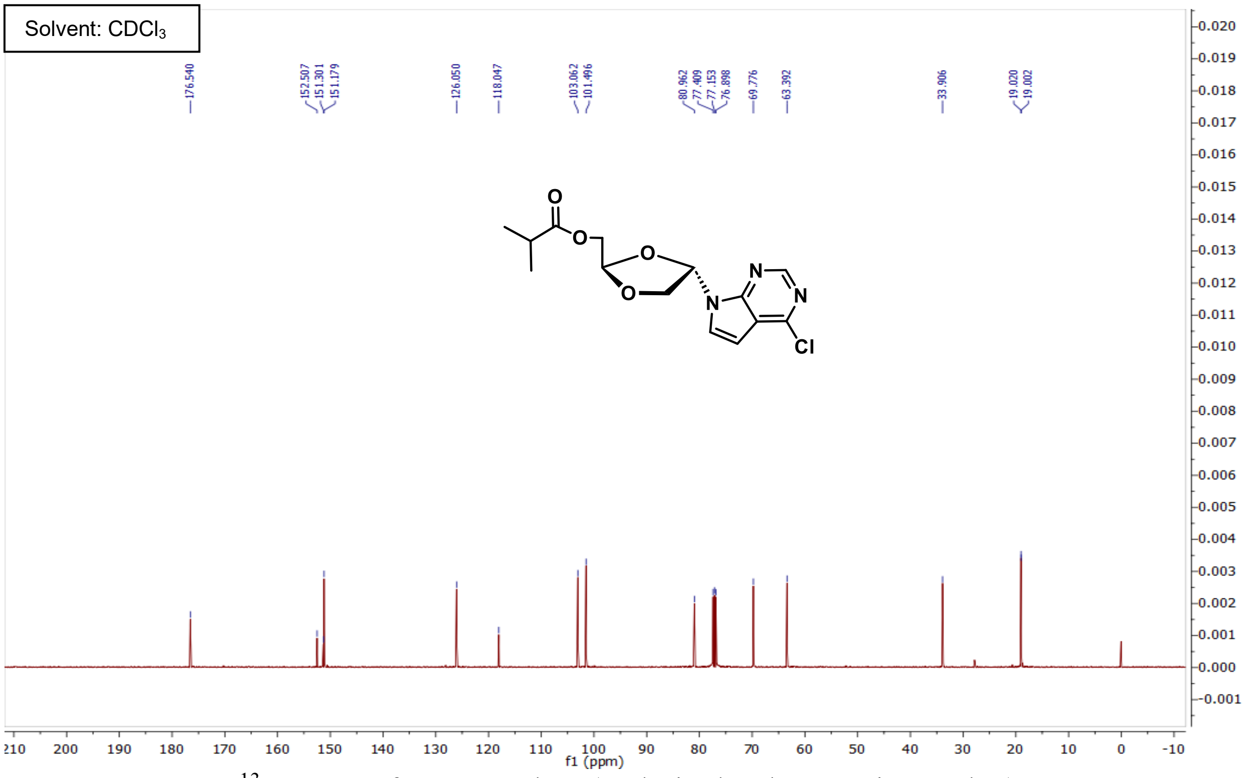

^{13}C NMR of compound **5a** (undesired *α*-deazapurine analog)

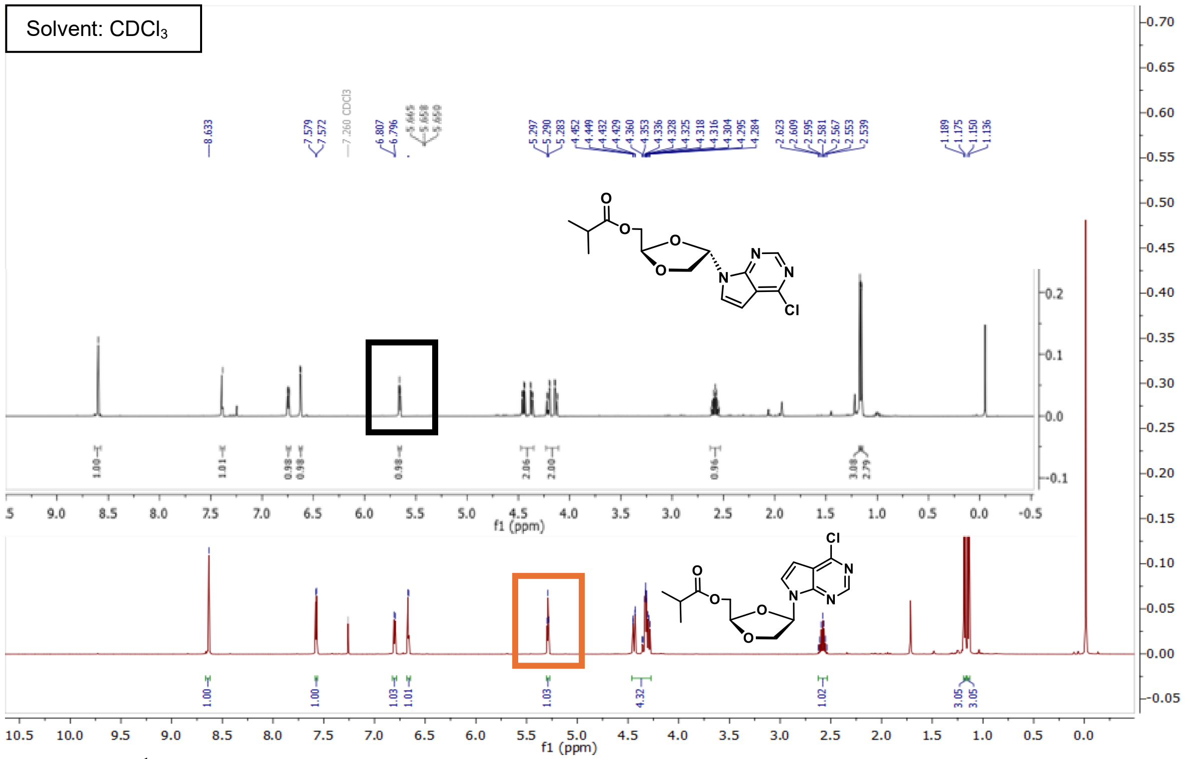

Overlayed ^{1}H NMRs of compound **5*β* and 5a *α***. The H4′ proton δ value changes significantly in correspondence with the configuration (either α or β) of the respective purine base.

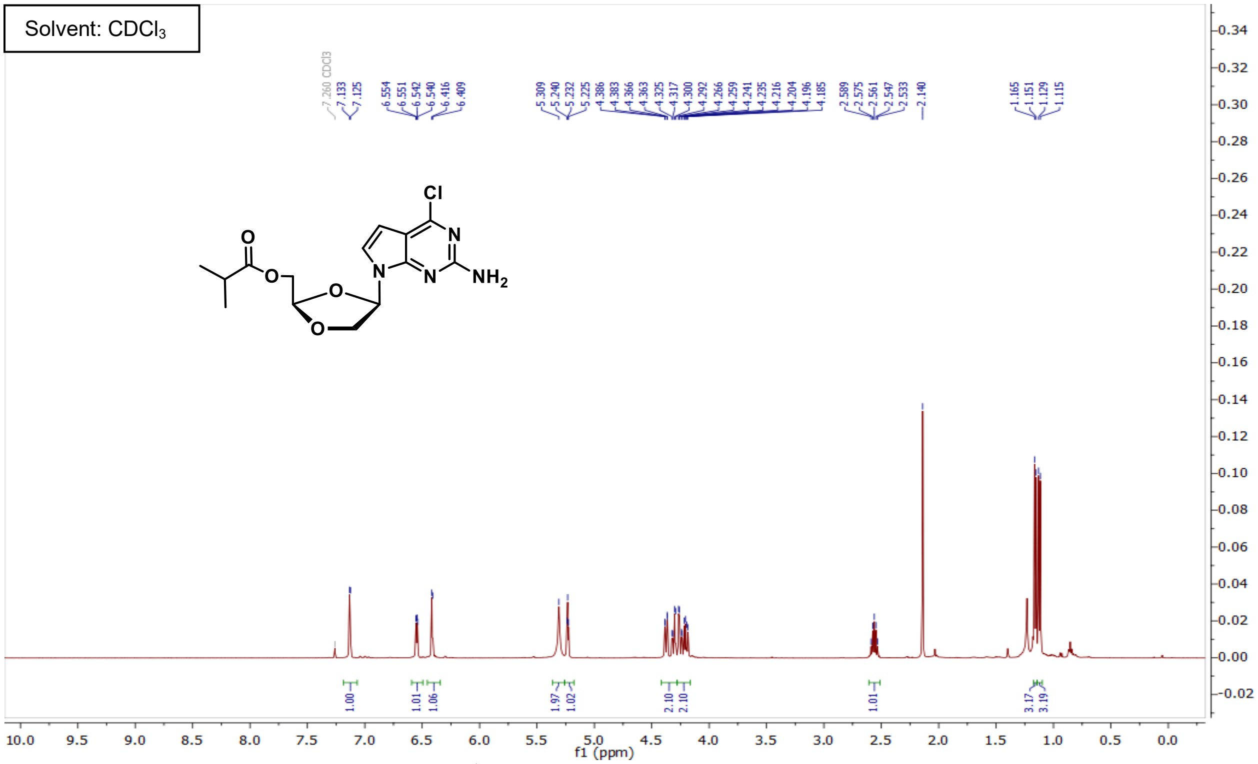

^{1}H NMR of compound **6**

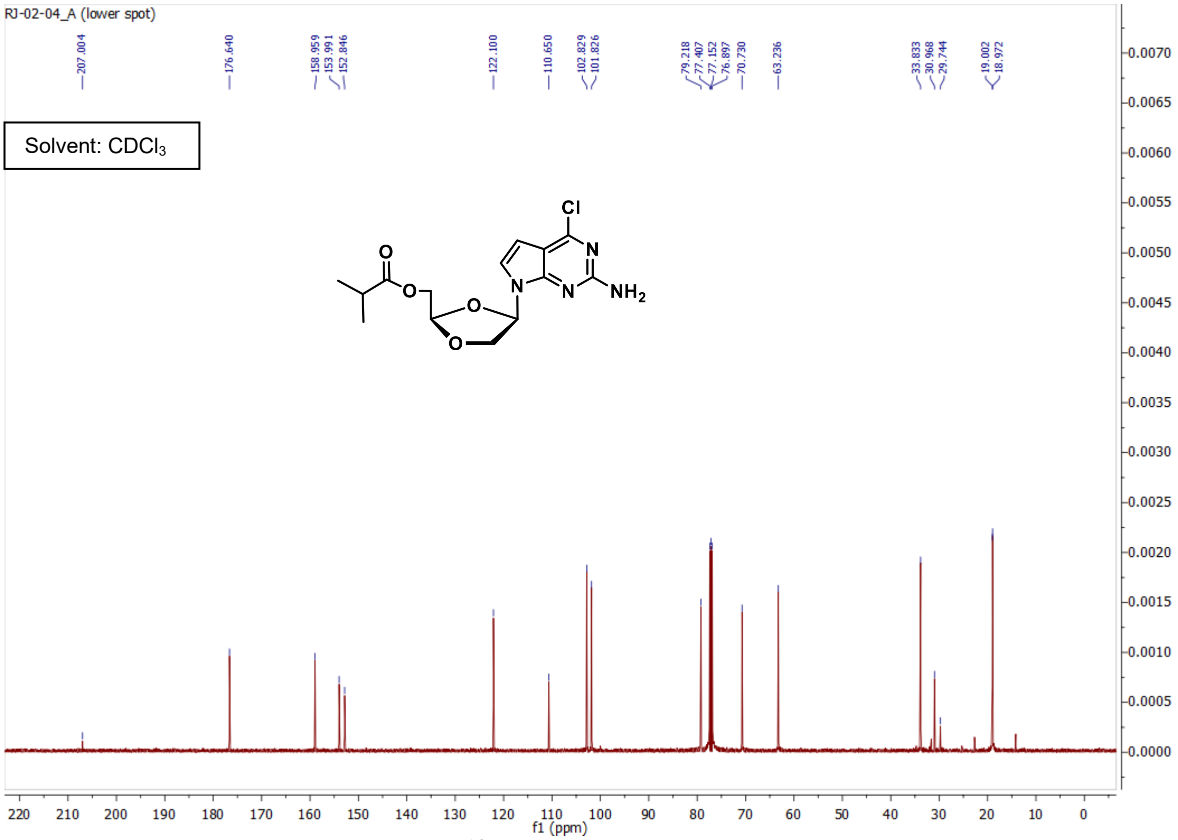

^{13}C NMR of compound **6**

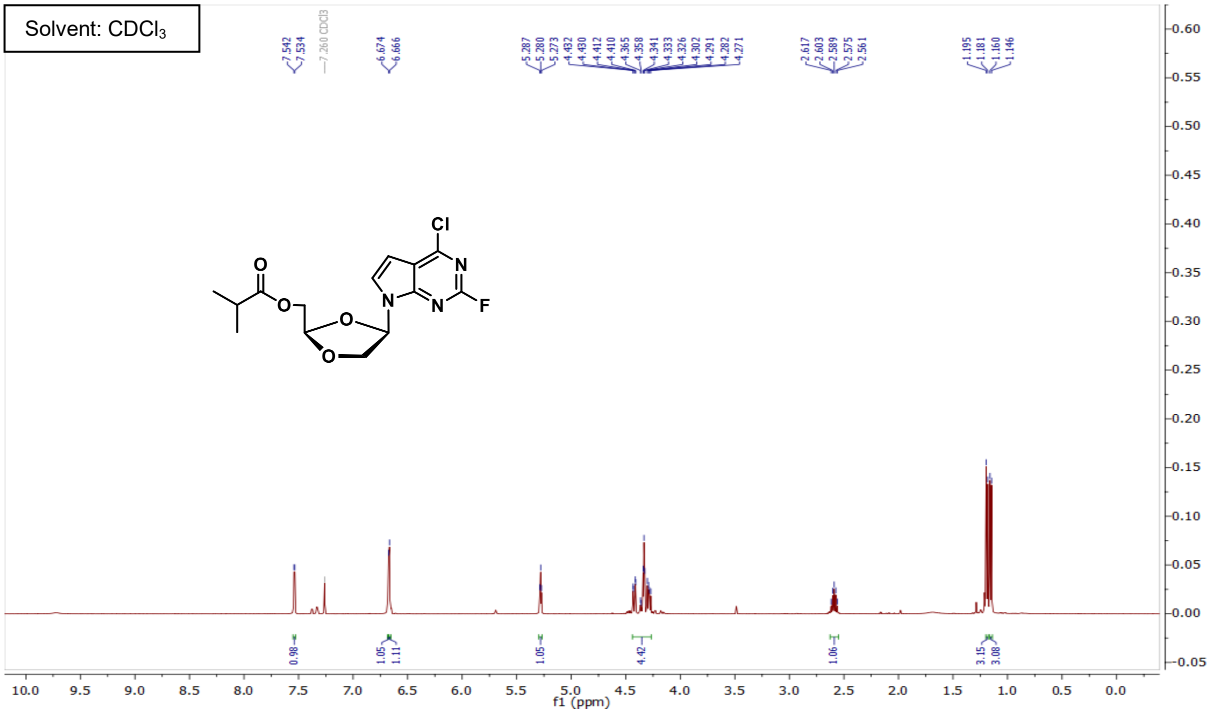

^{1}H NMR of compound **7**

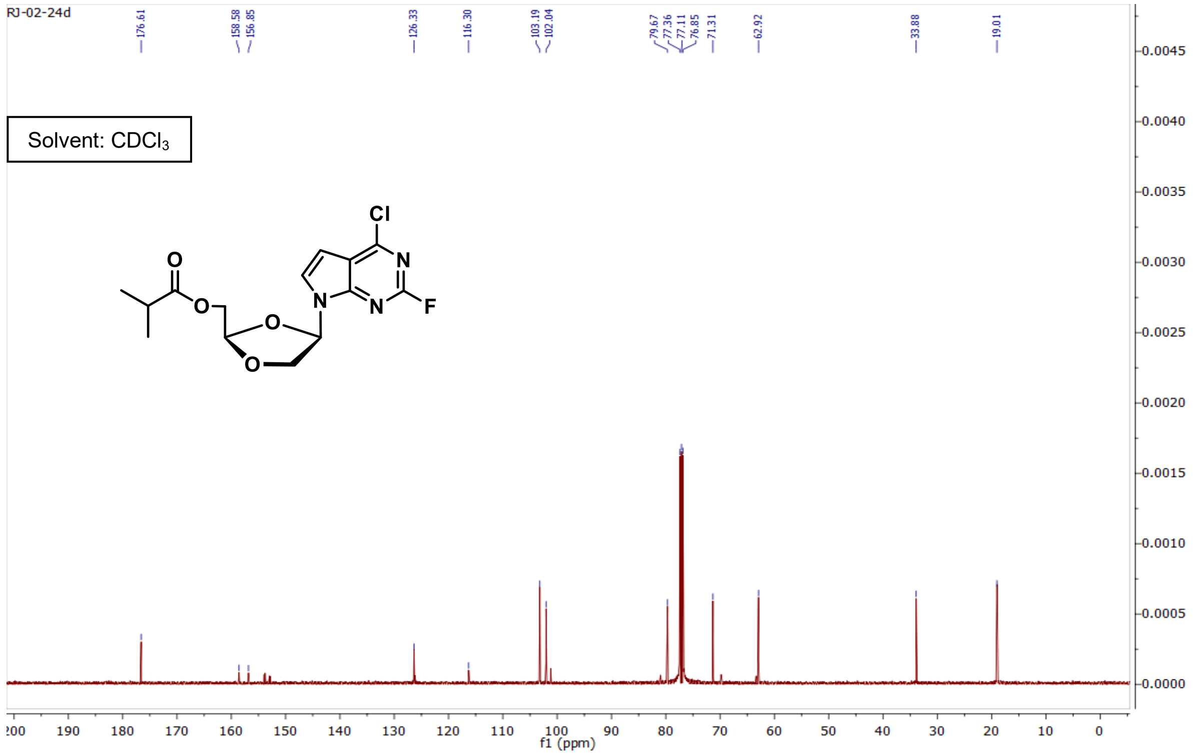

^{13}C NMR of compound **7**

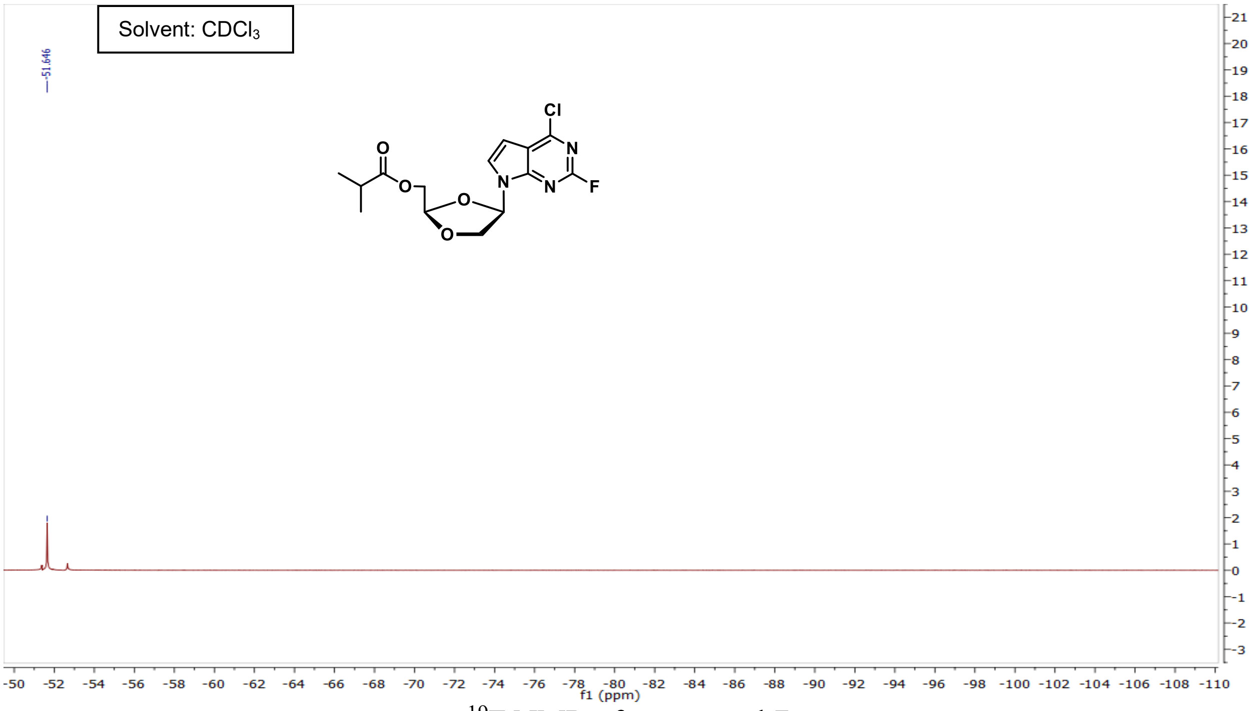

^{19}F NMR of compound **7**

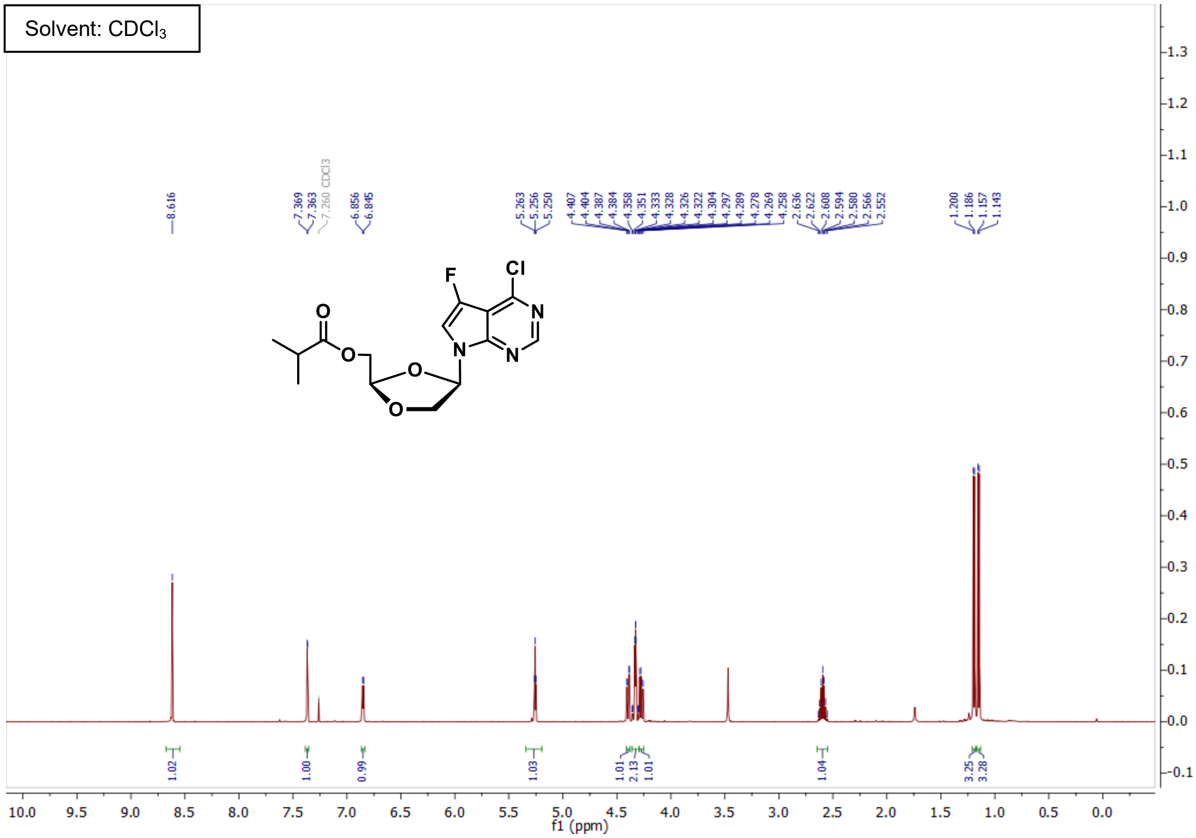

^{1}H NMR of compound **8**

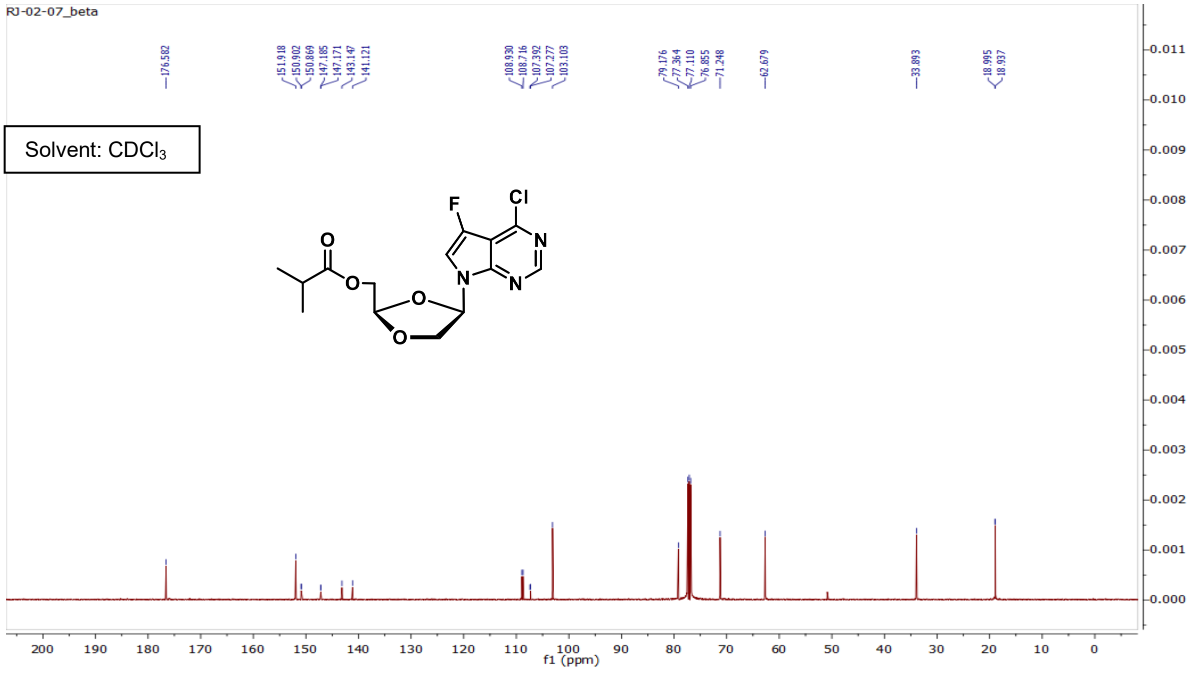

^{13}C NMR of compound **8**

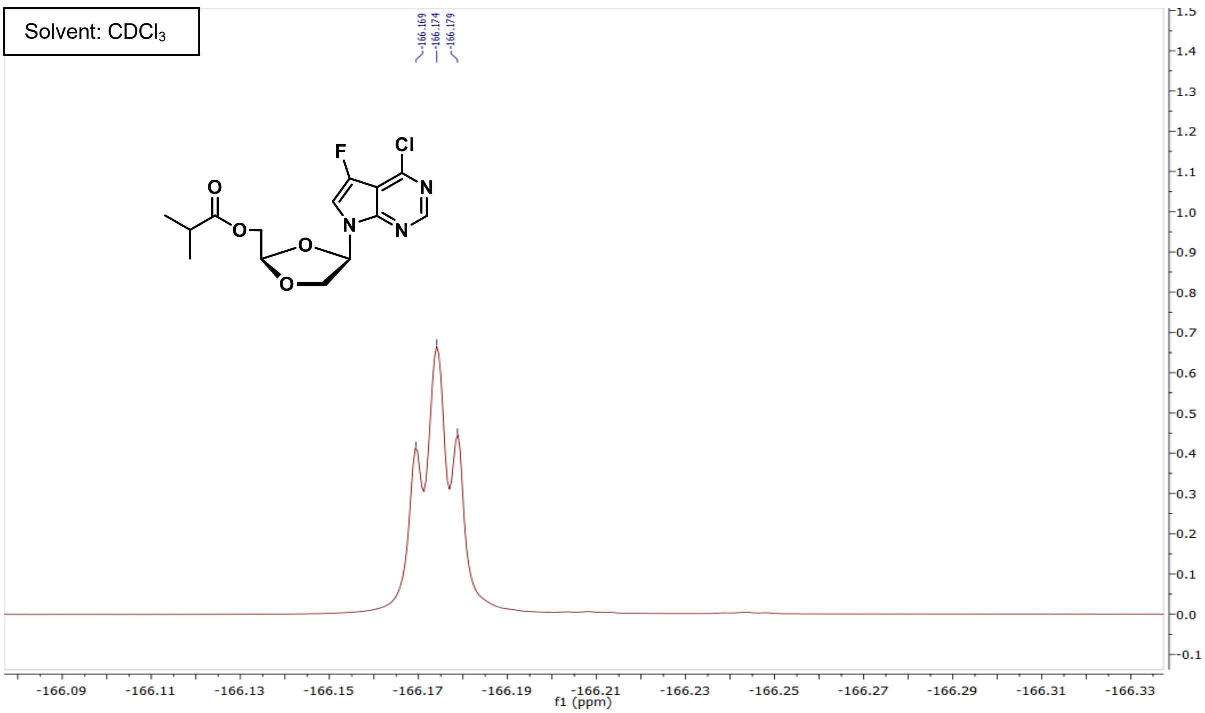

^{19}F NMR of compound **8**

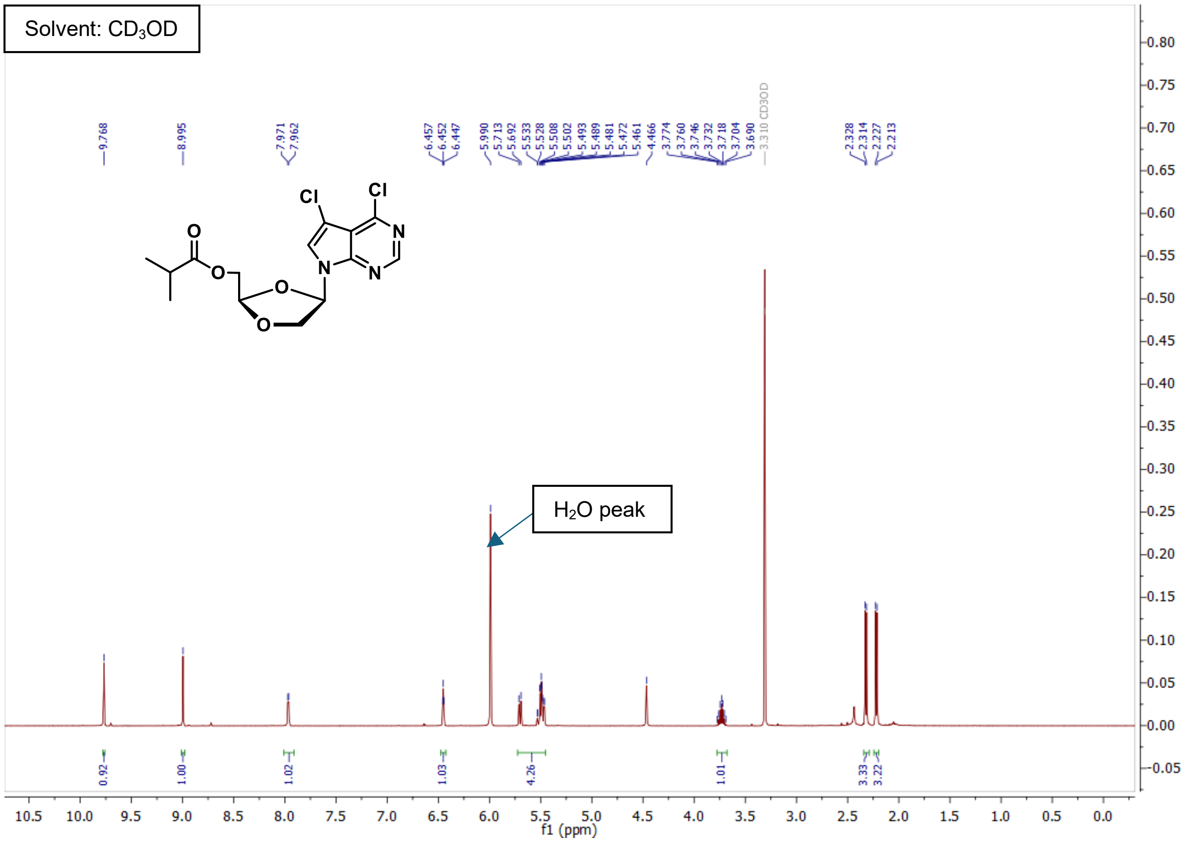

^{1}H NMR of compound **9**

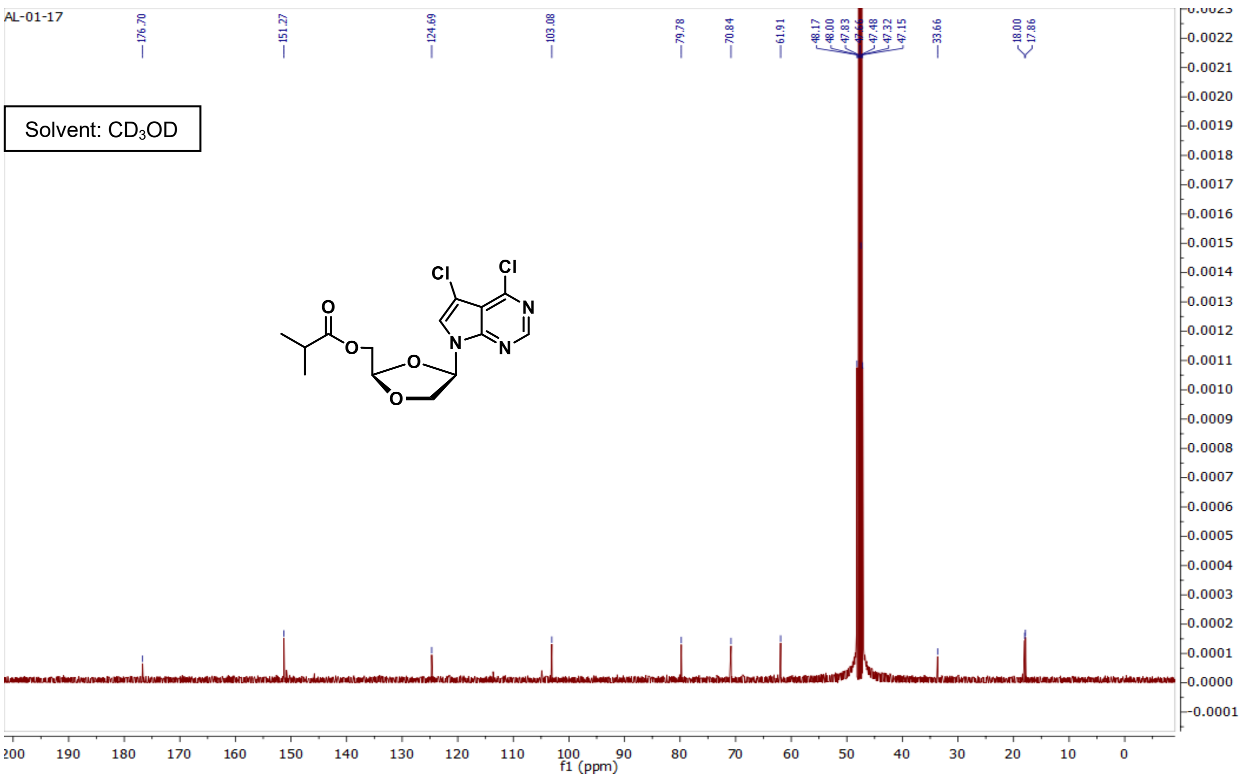

^{13}C NMR of compound **9**

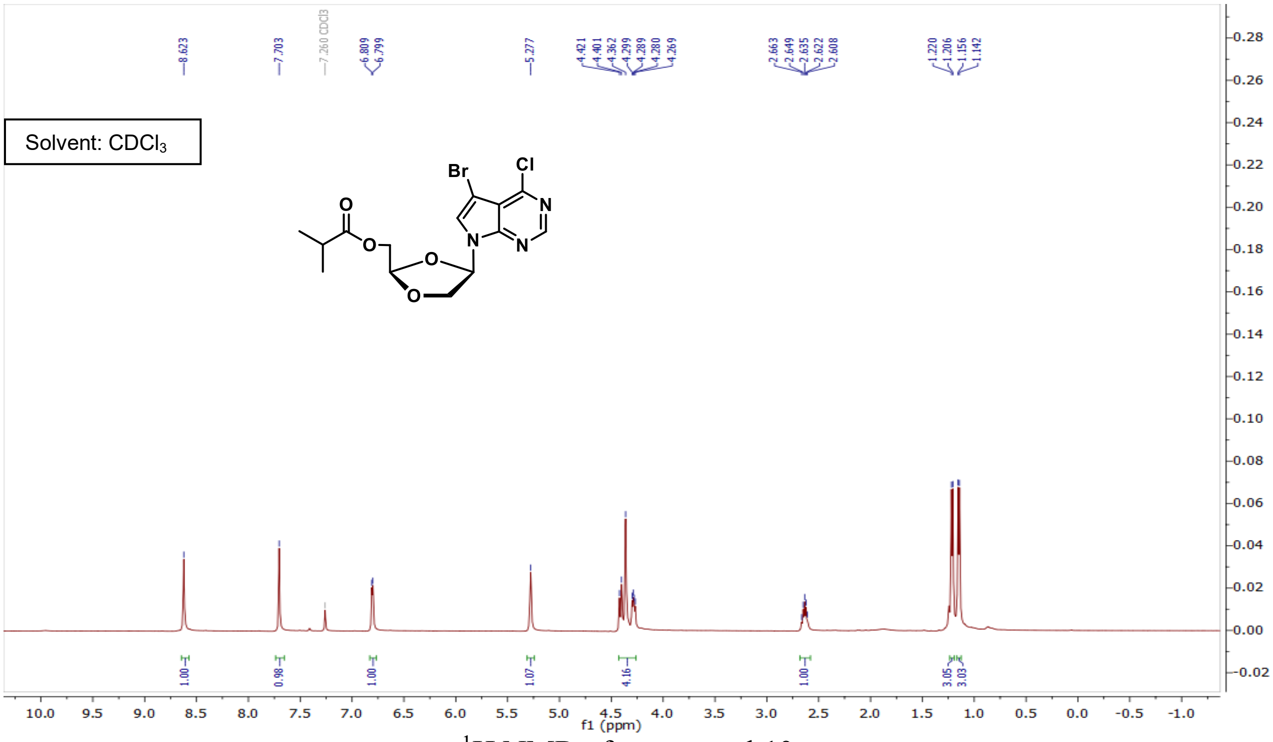

^{1}H NMR of compound **10**

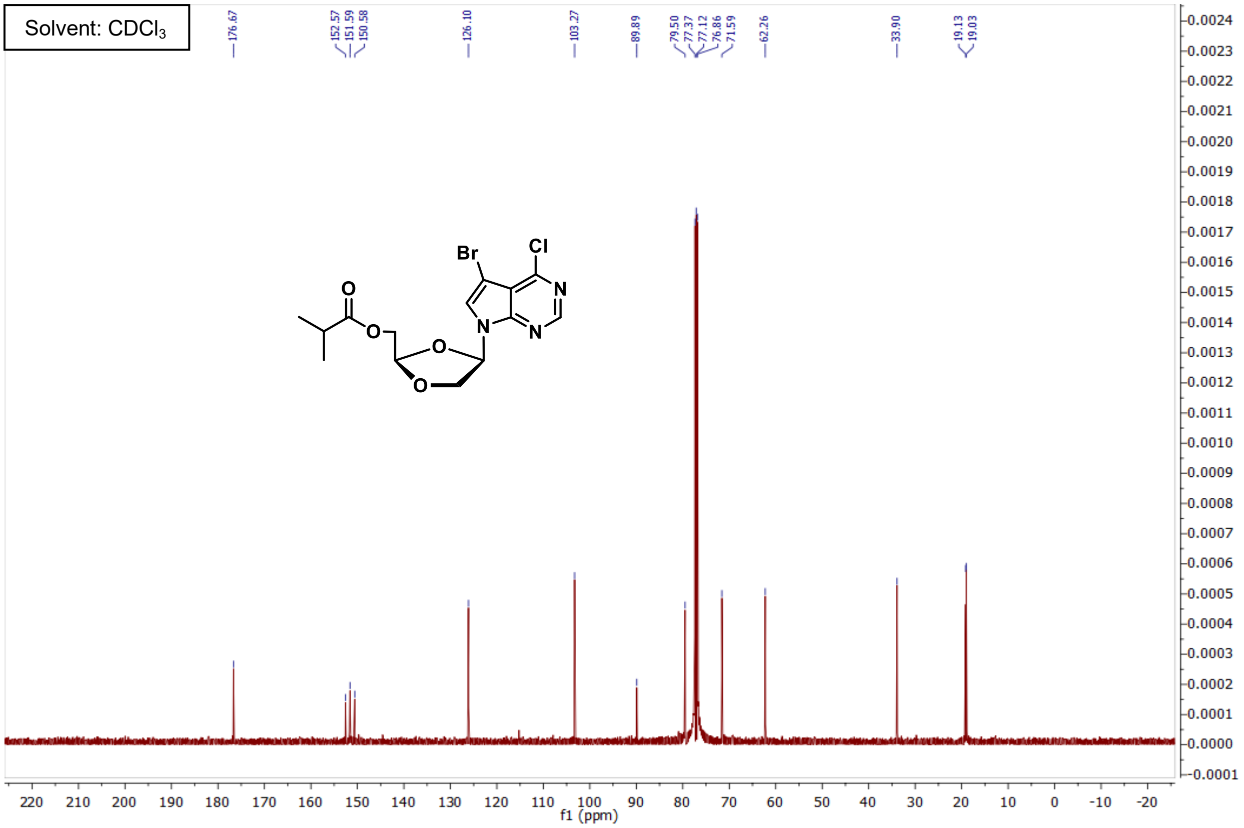

^{13}C NMR of compound **10**

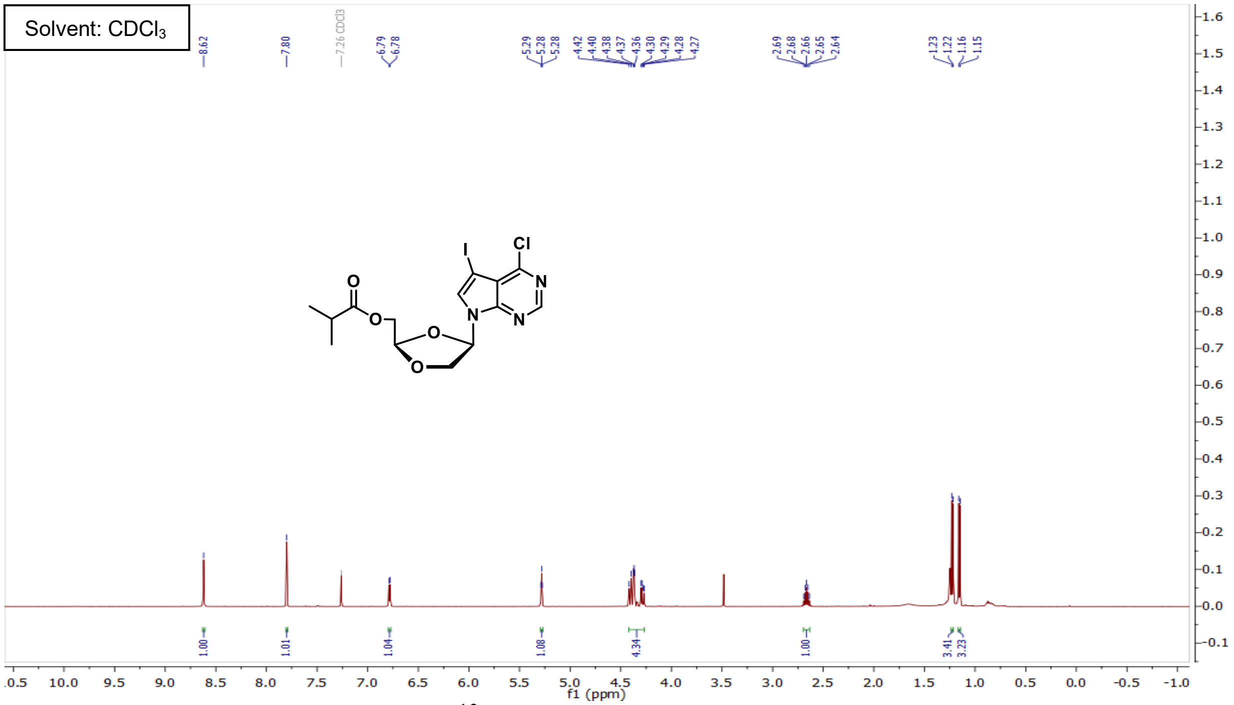

^{13}C NMR of compound **11**

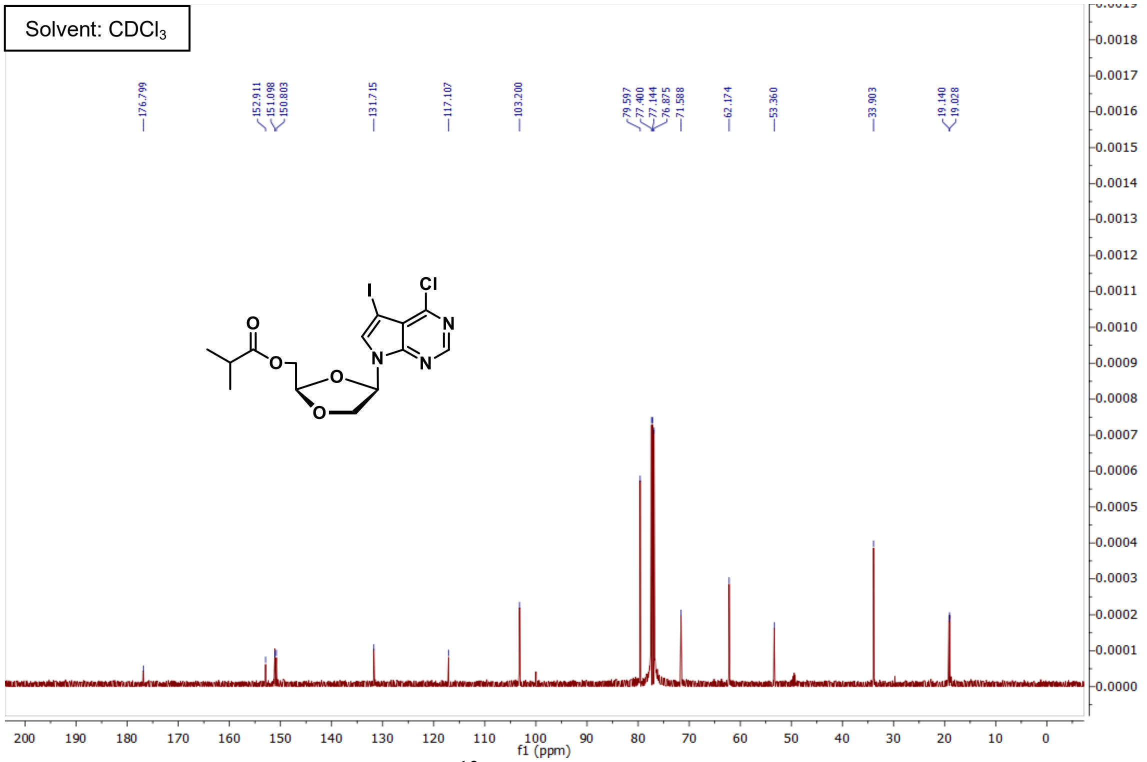

^{13}C NMR of compound **11**

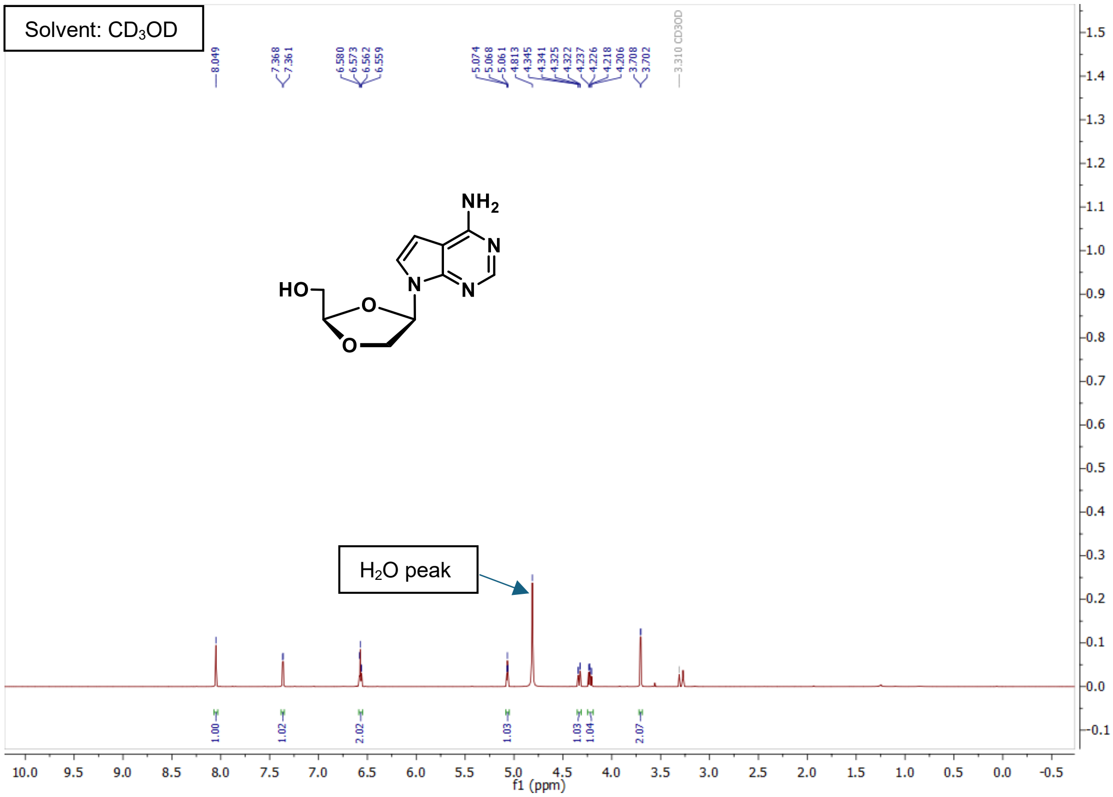

^{1}H NMR of compound **12**

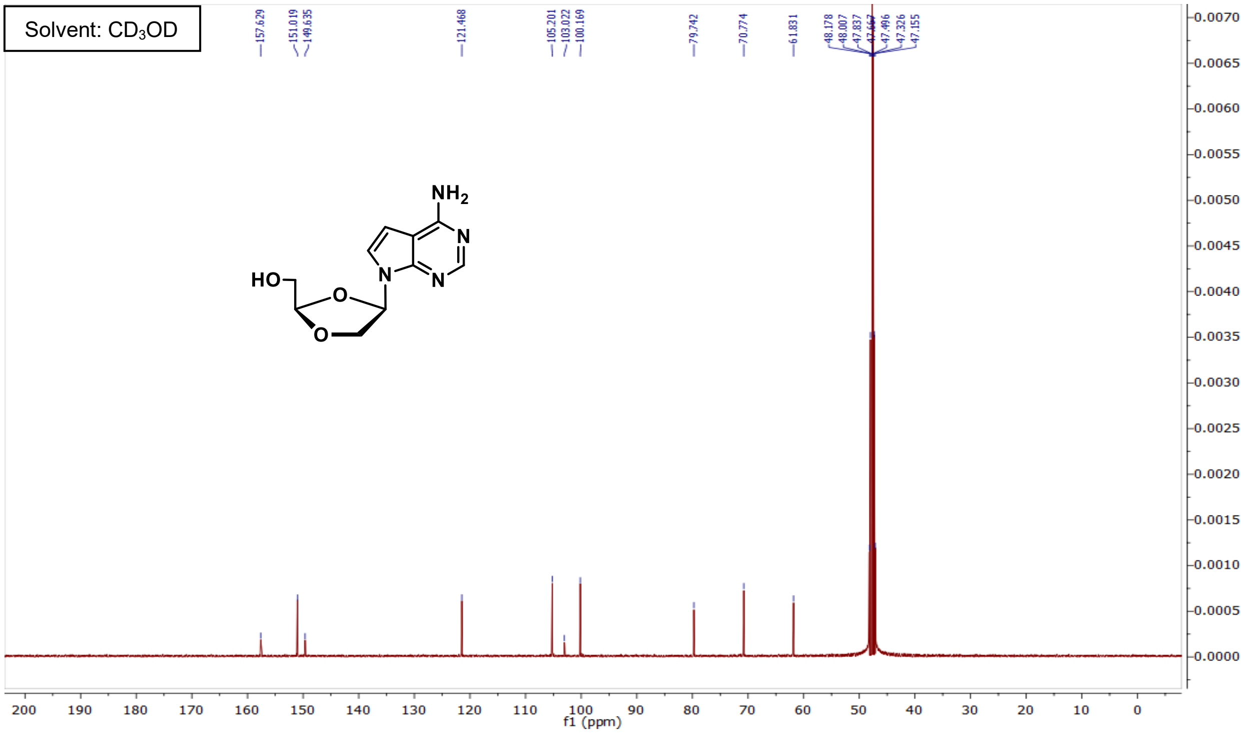

^{13}C NMR of compound **12**

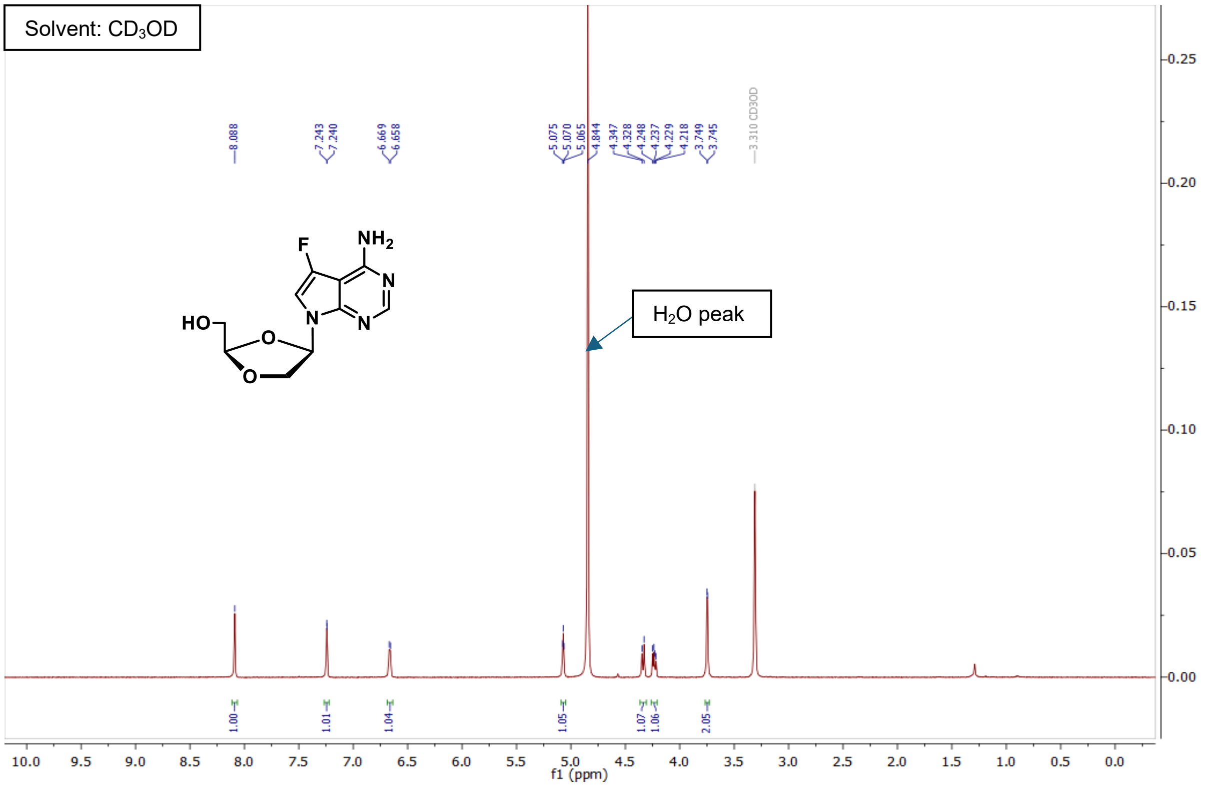

^{1}H NMR of compound **13**

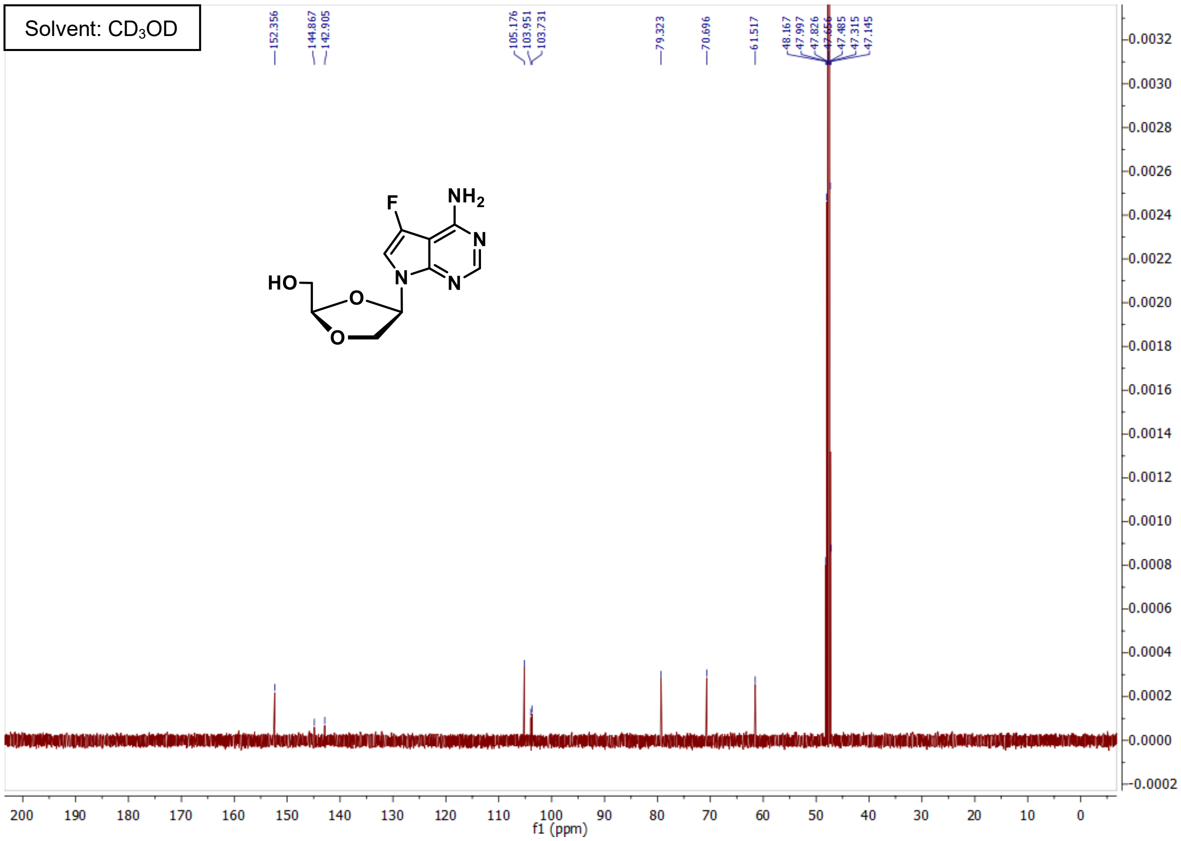

^{13}C NMR of compound **13**

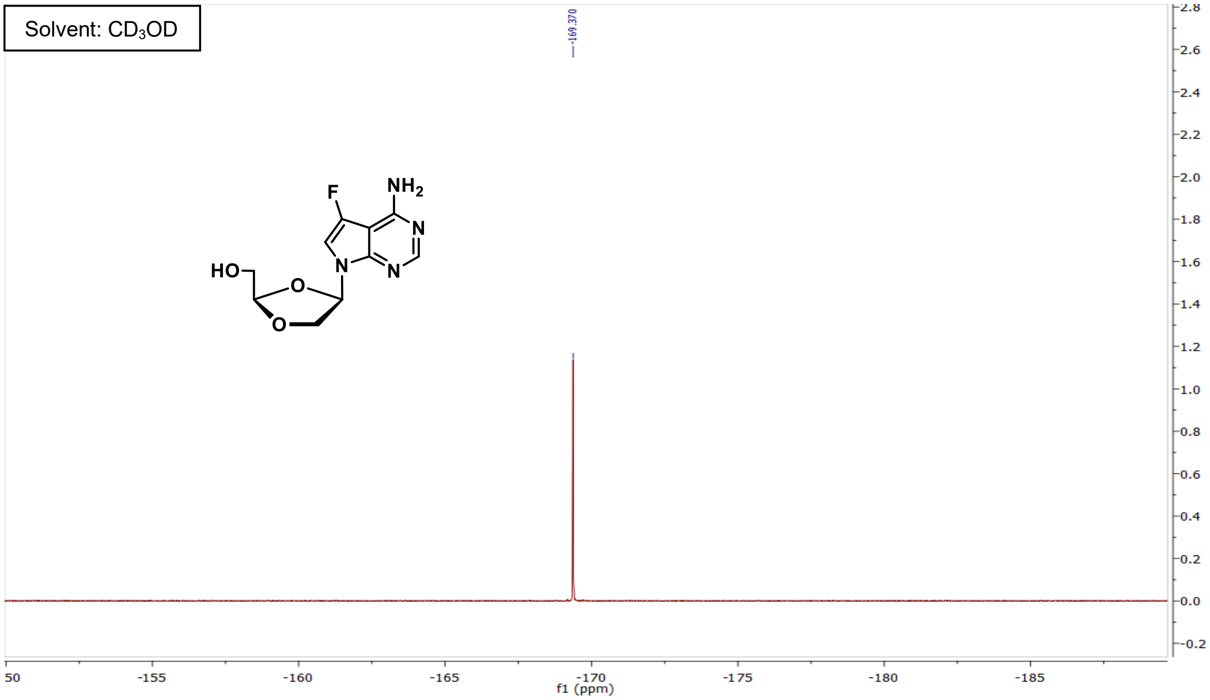

^{19}F NMR of compound **13**

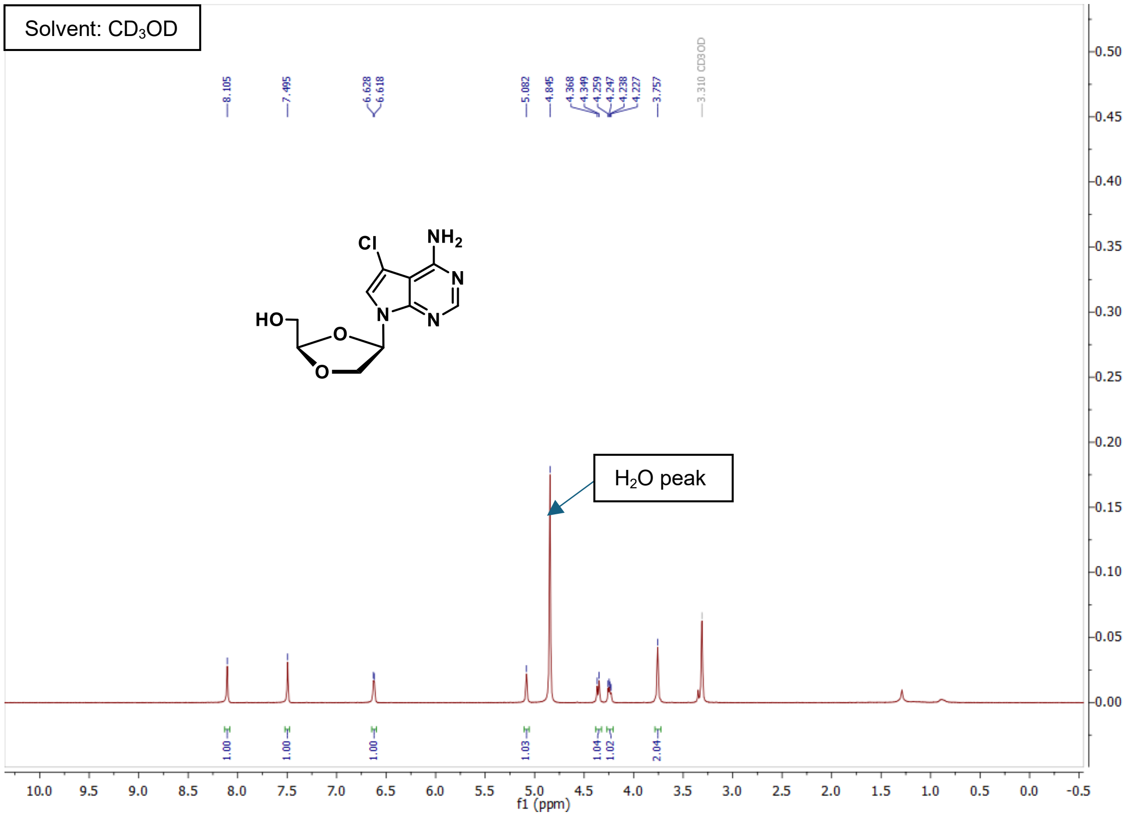

^{1}H NMR of compound **14**

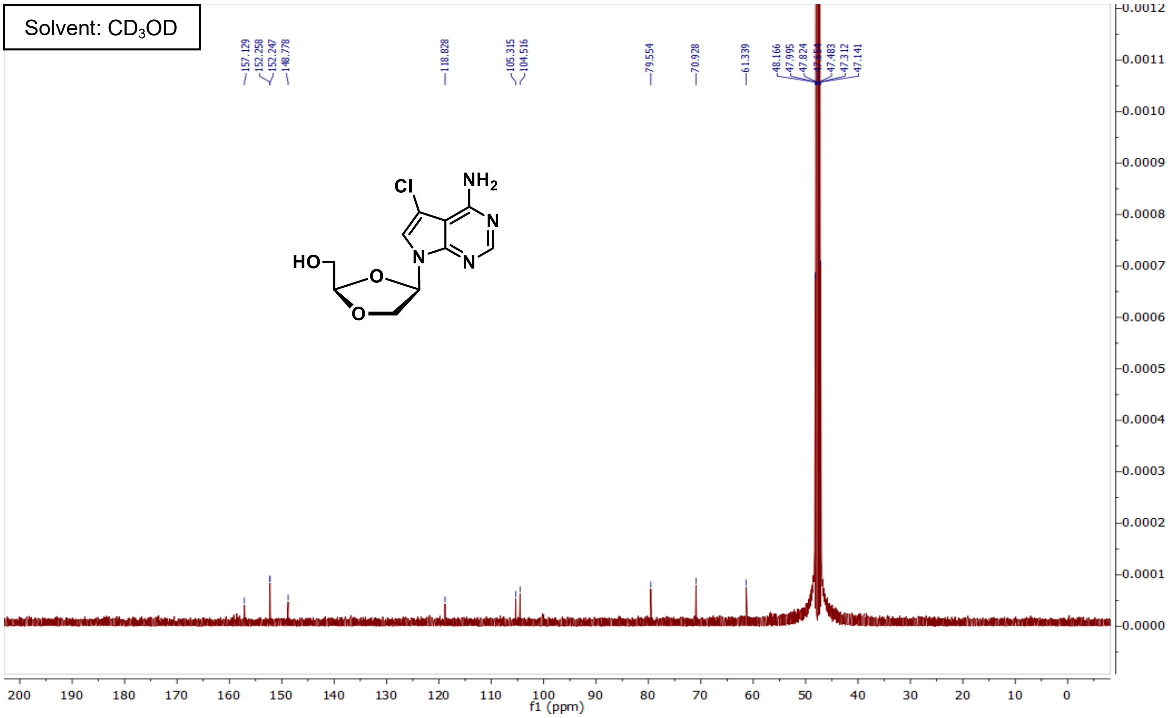

^{13}C NMR of compound **14**

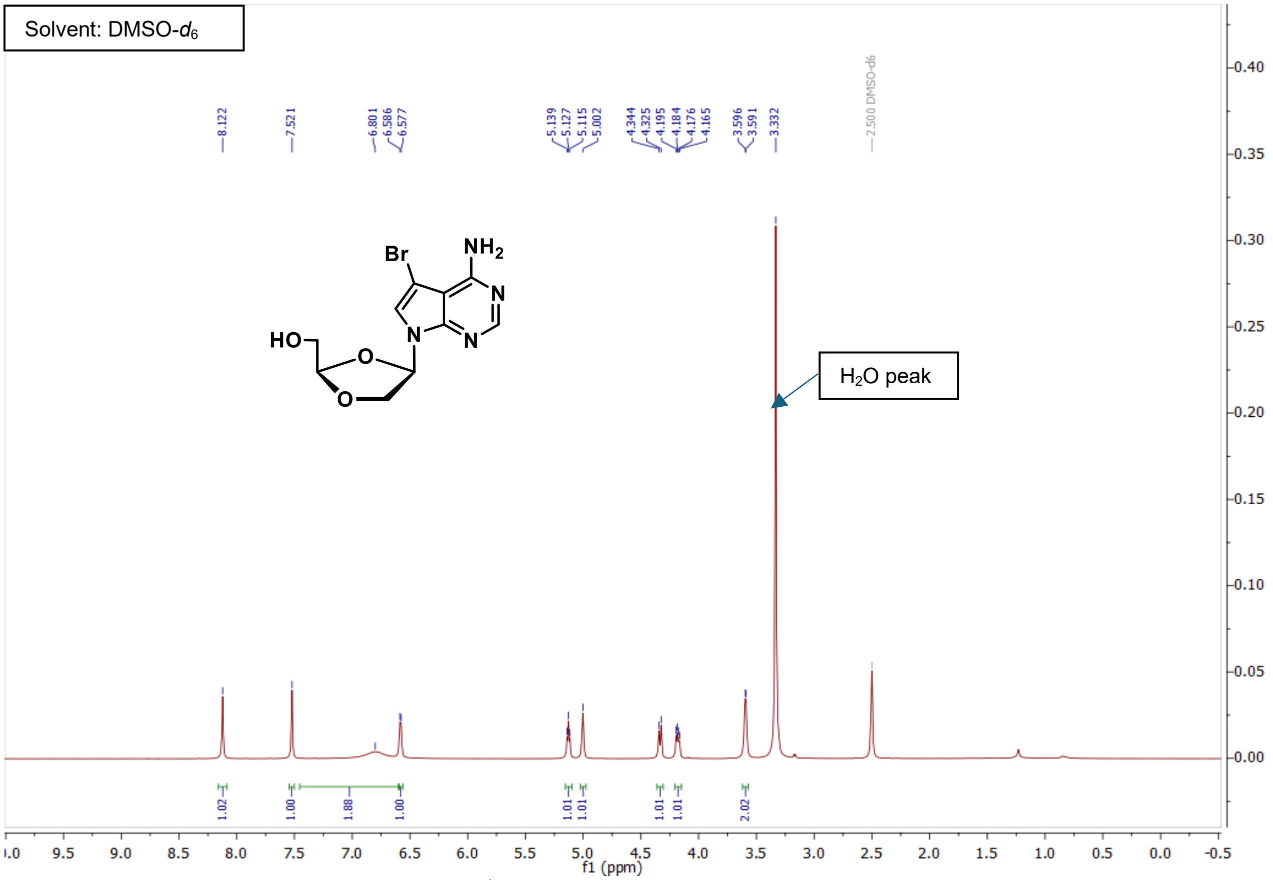

^{1}H NMR of compound **15**

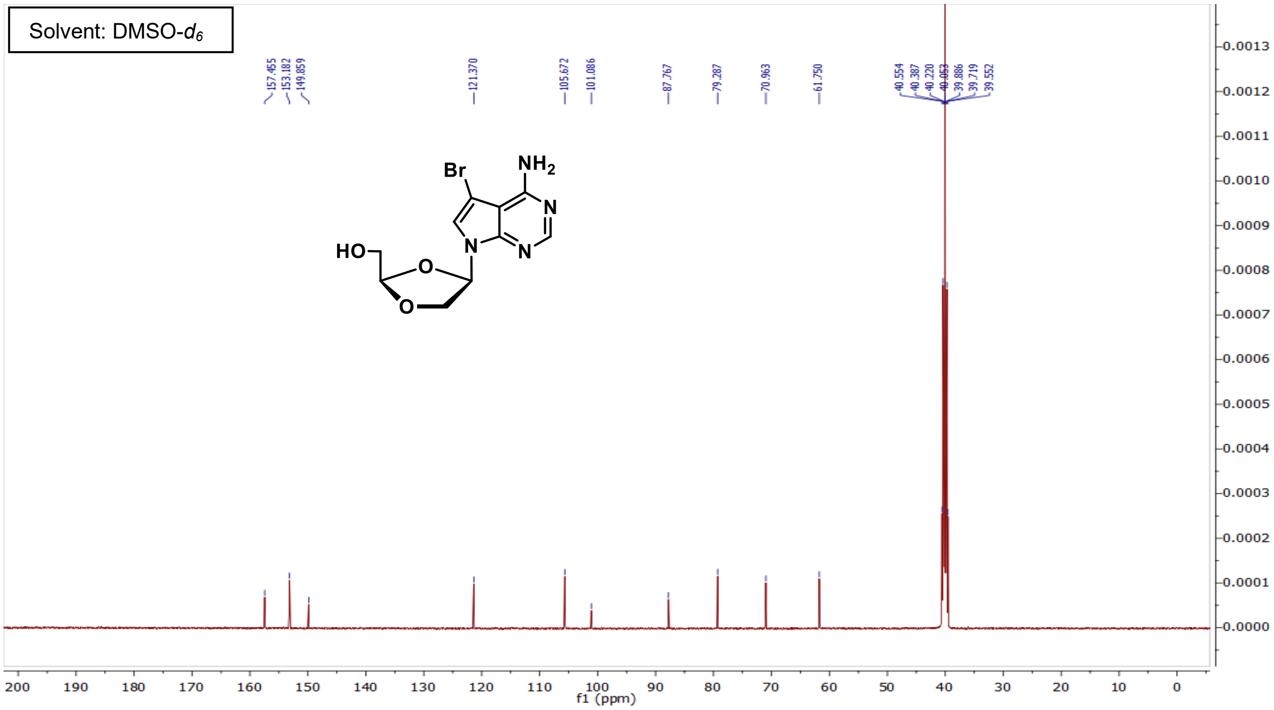

^{13}C NMR of compound **15**

HPLC Method Conditions			
Column	XBridge C18 (4.6 x 150) mm, 3.5µm	Sample	RJ-02-28
Mobile Phase-A	10mM Ammonium Bicarbonate in Water	Vail	70
Mobile Phase-B	100% Acetonitrile	Injection Volume	0.50µL
Gradient (T% B)	0/5, 2/5, 10/50, 15/95, 15.1/5, 20/5	Run time	20.0 minutes
Flow Rate	0.8mL/min	Proc. Chnl. Descr.:	2998 PDA MaxPlot (190.0 nm to 800.0 nm) (2998(210-400) nm) Subtracted from blank
Column Oven Temp.	30 °C	Acq. Method Set:	Method 1
Diluent	MeOH+Water		

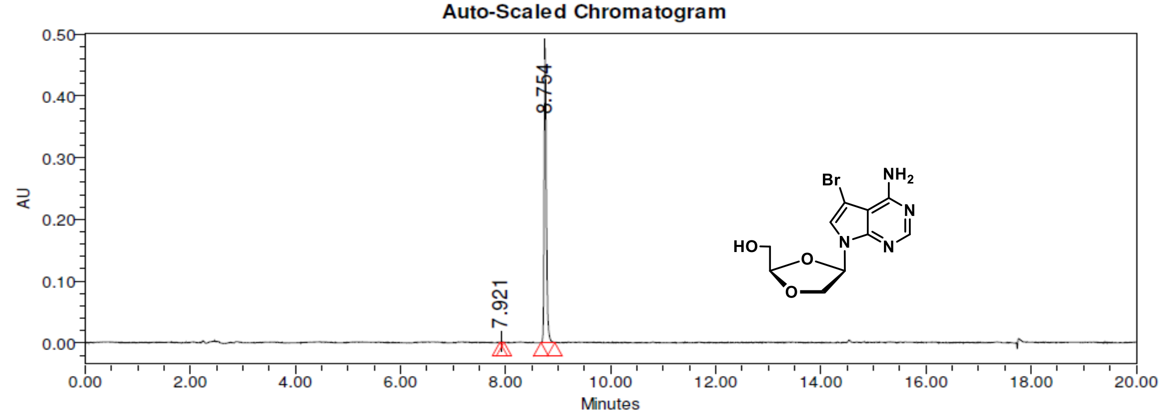

Peak Results

	RT	Area	Height	% Area
1	7.921	2606	1136	0.19
2	8.754	1364726	477213	99.81

HPLC analysis of compound **15**

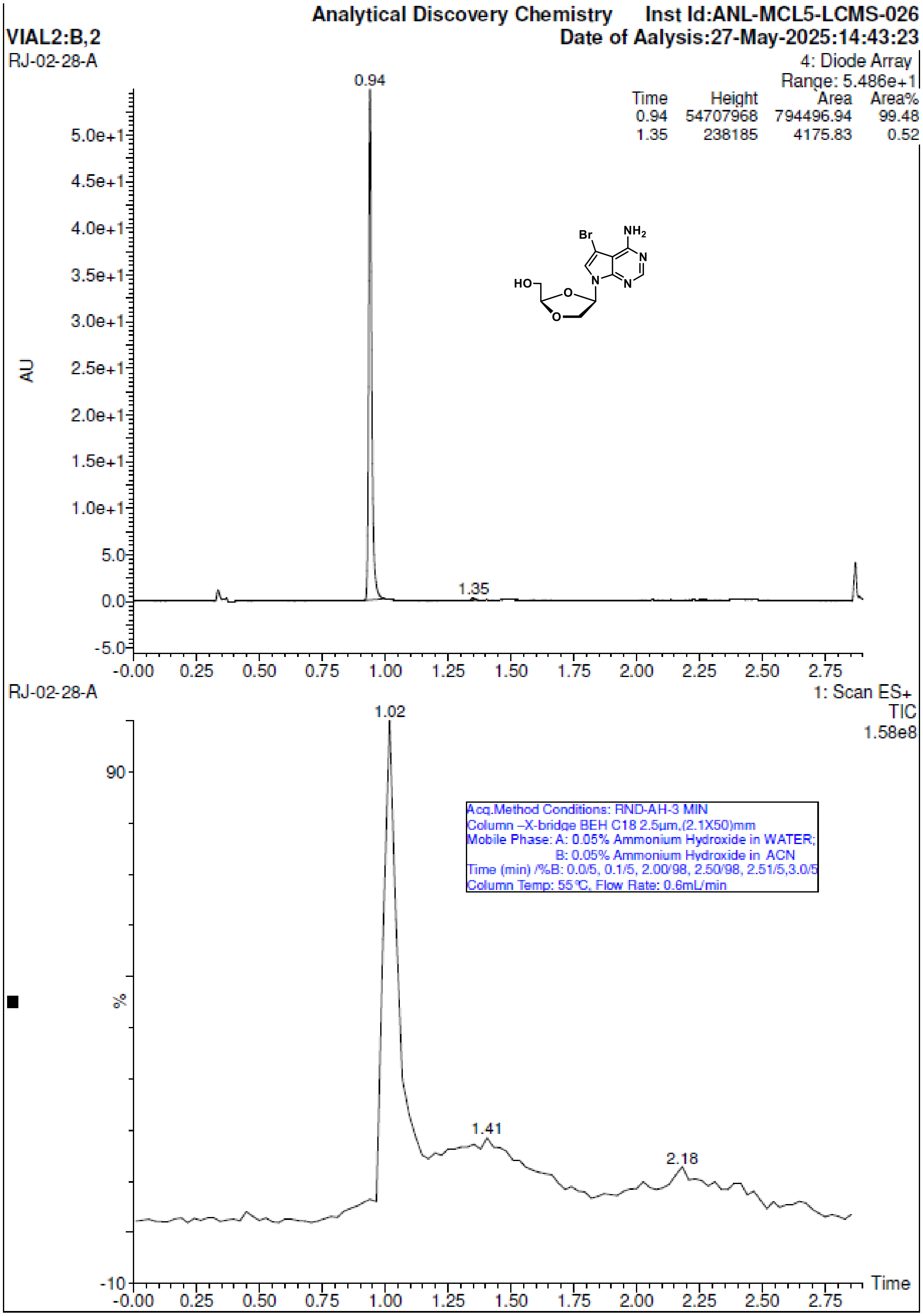

LC-MS analysis of compound **15**

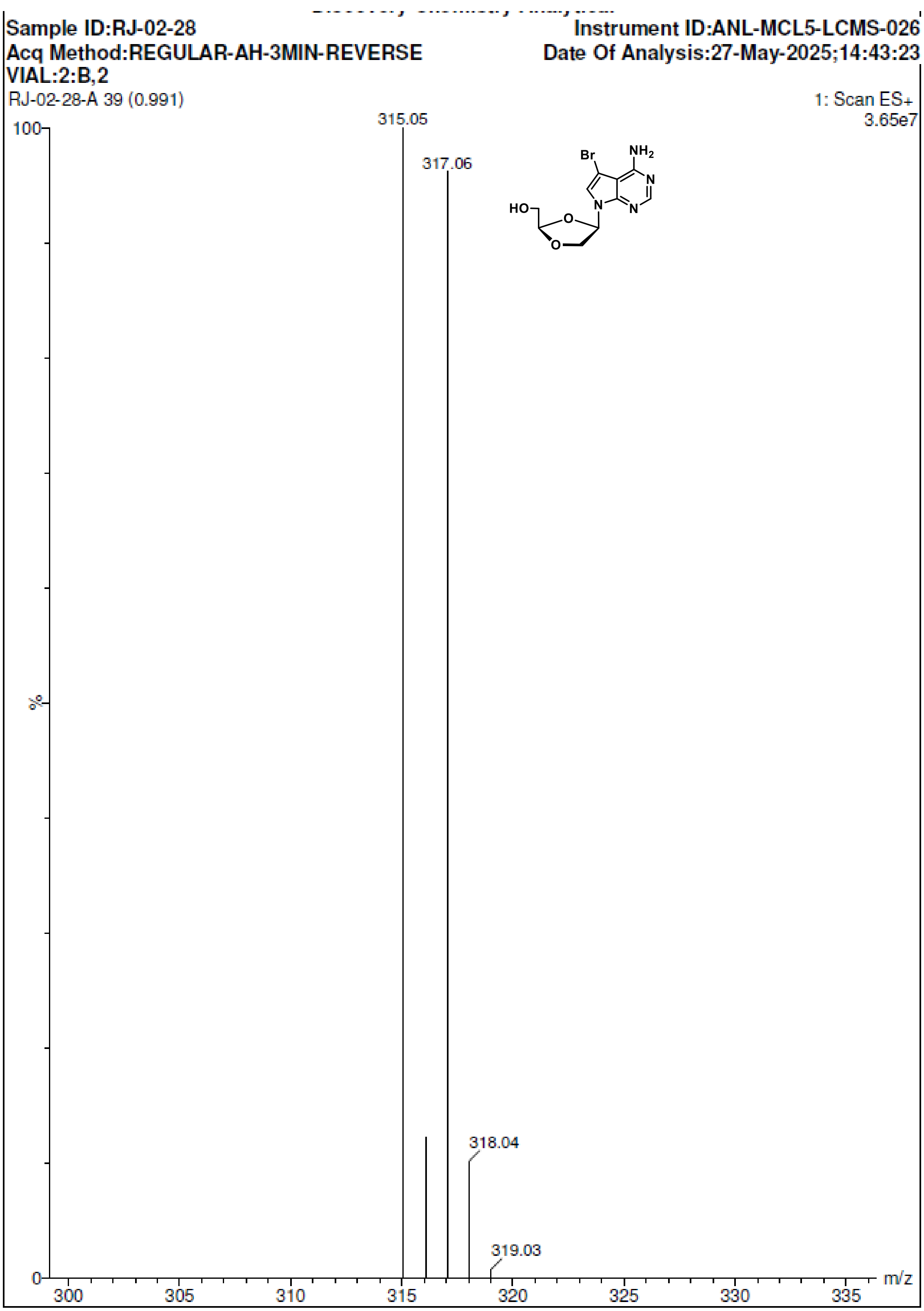

LC-MS analysis of compound **15**

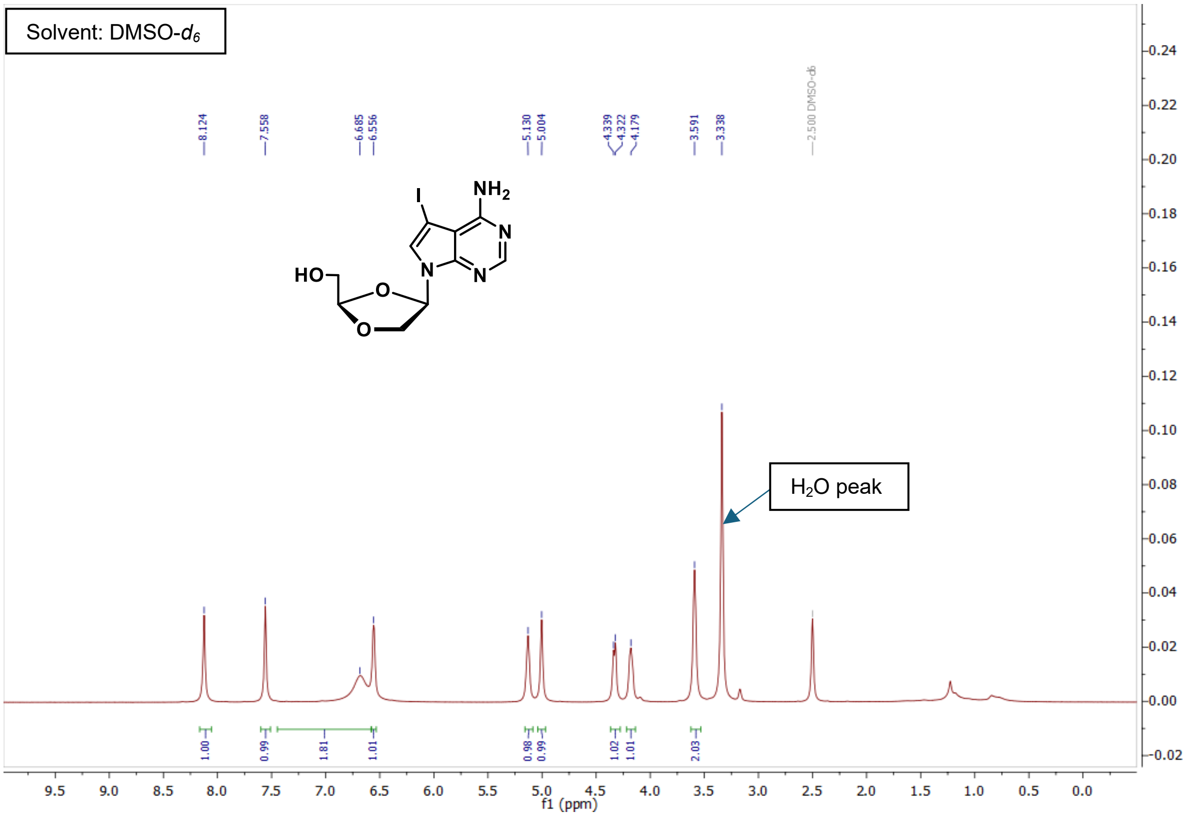

^{1}H NMR of compound **16**

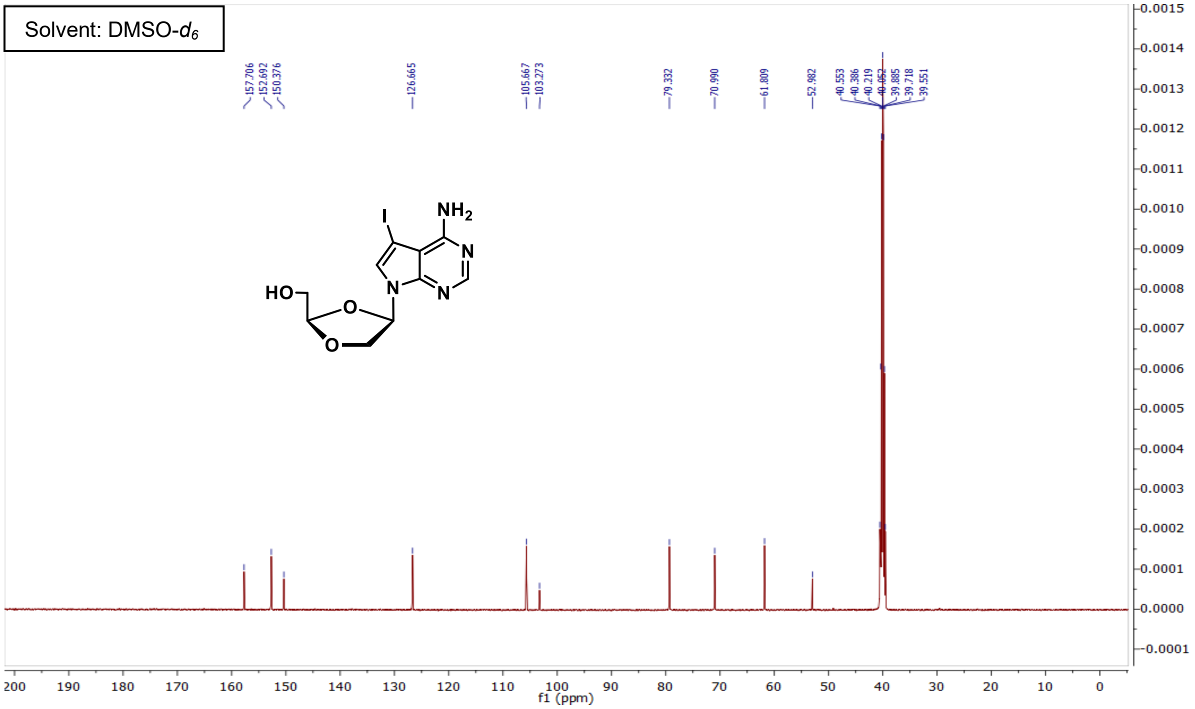

^{13}C NMR of compound **16**

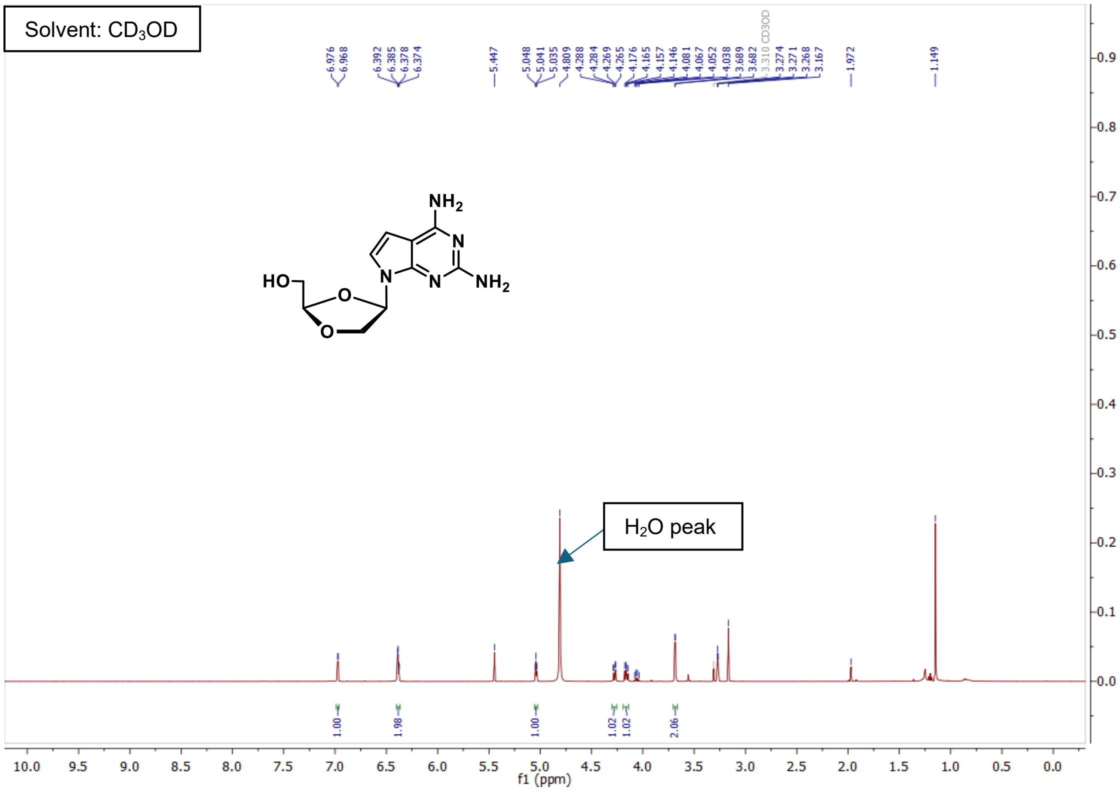

^{1}H NMR of compound **17**

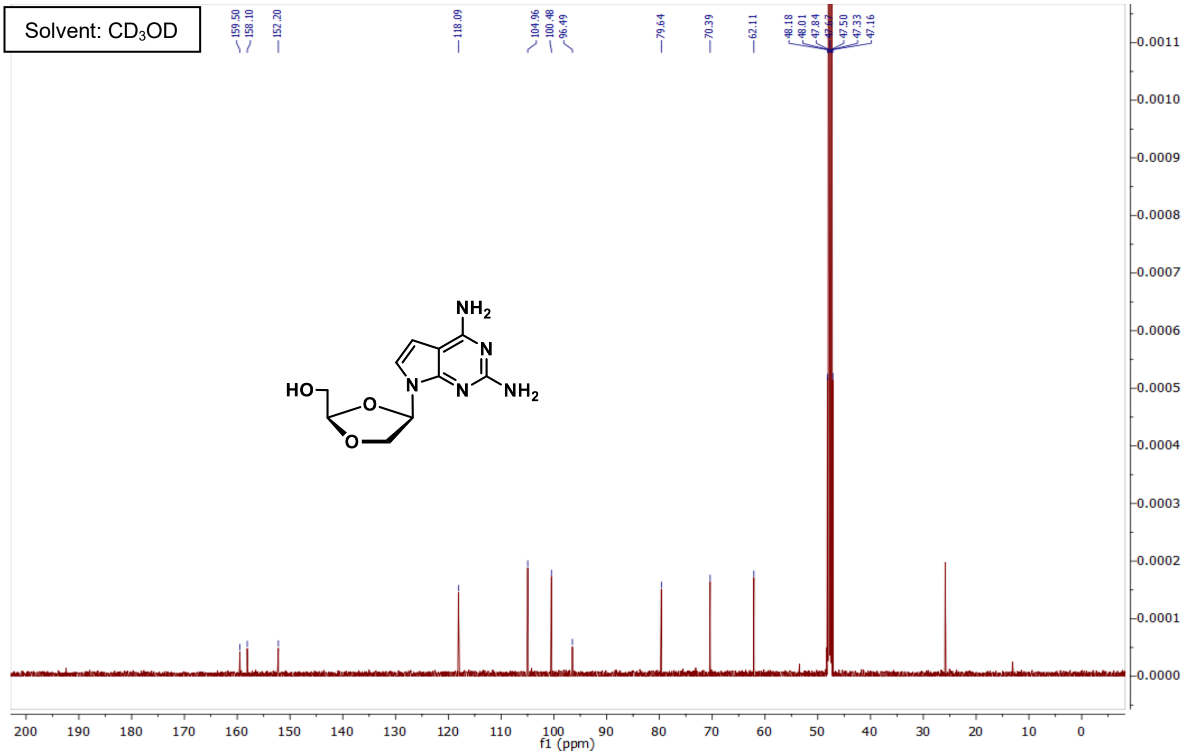

^{13}C NMR of compound **17**

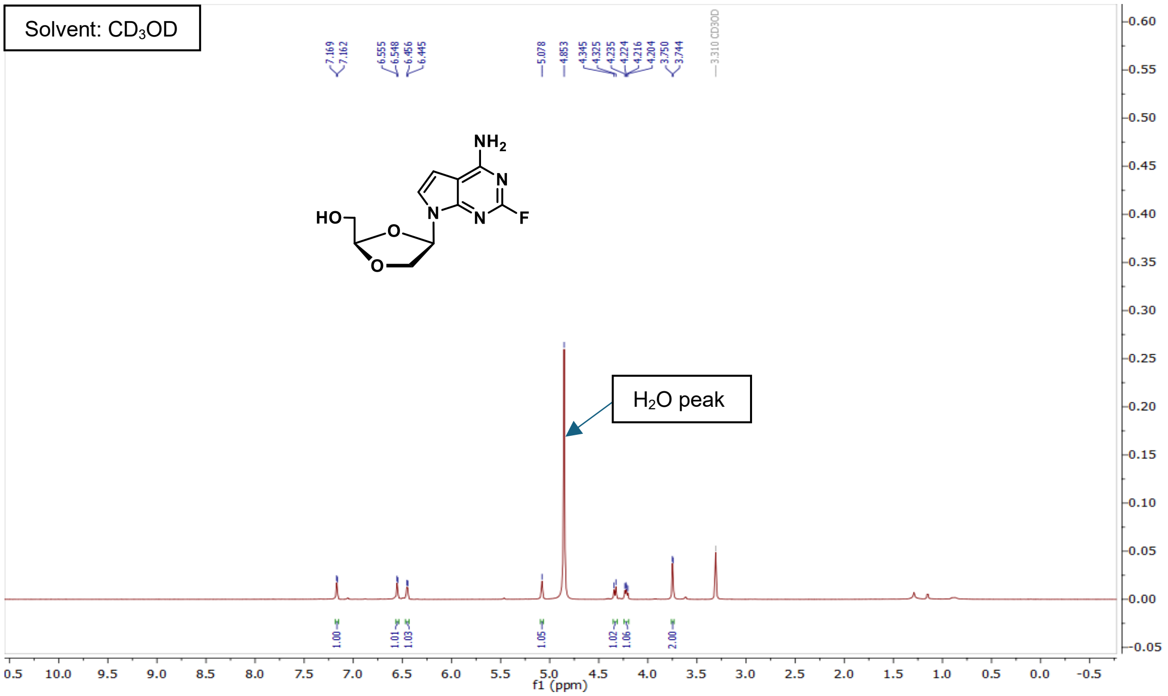

^{1}H NMR of compound **18**

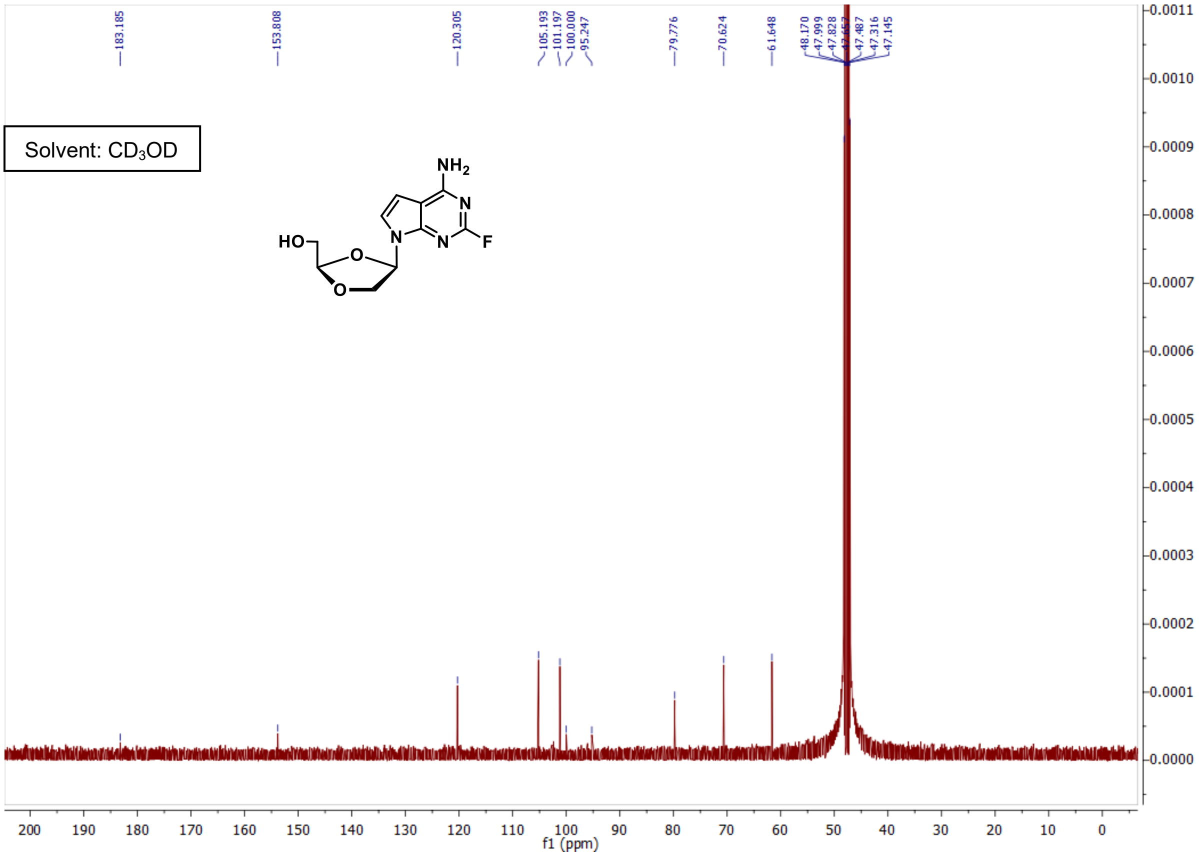

^{13}C NMR of compound **18**

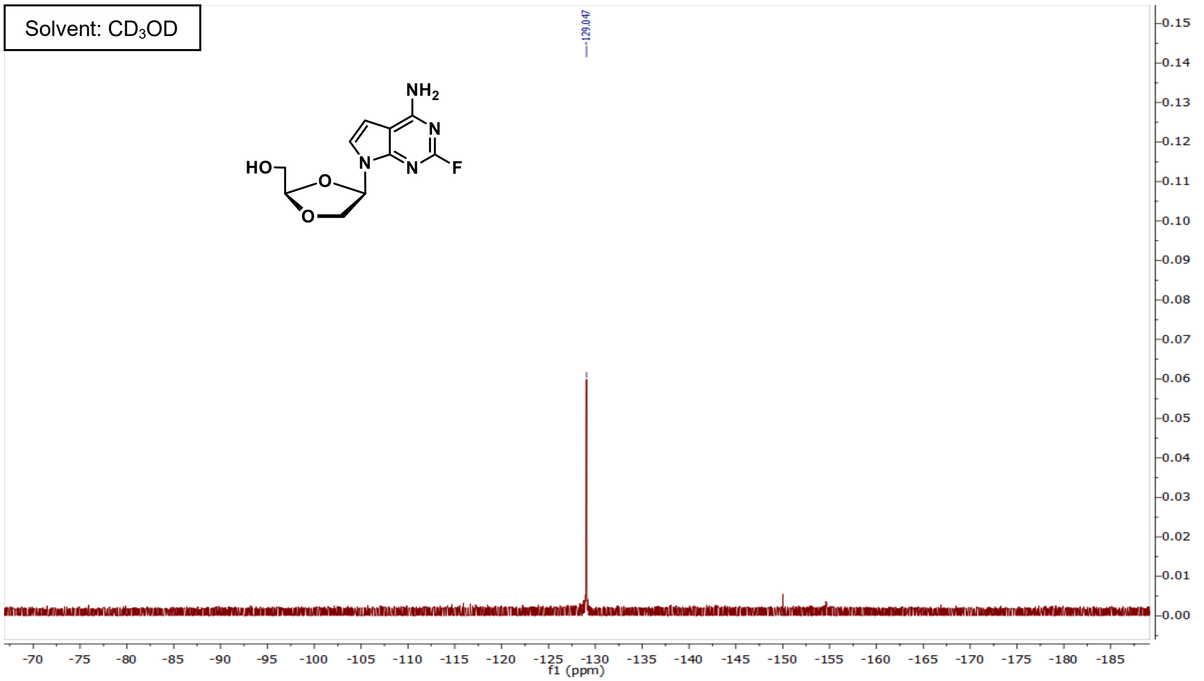

^{19}F NMR of compound **18**

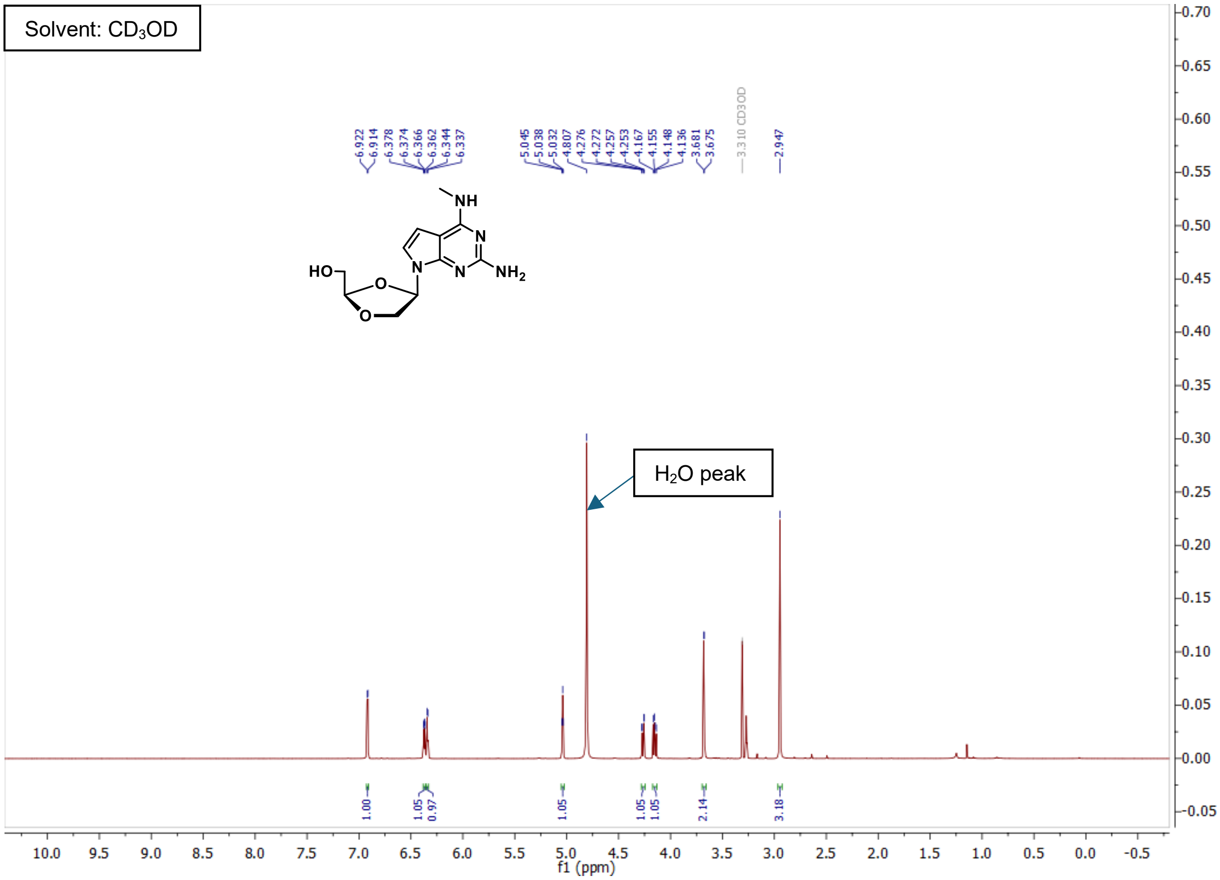

^{1}H NMR of compound **19**

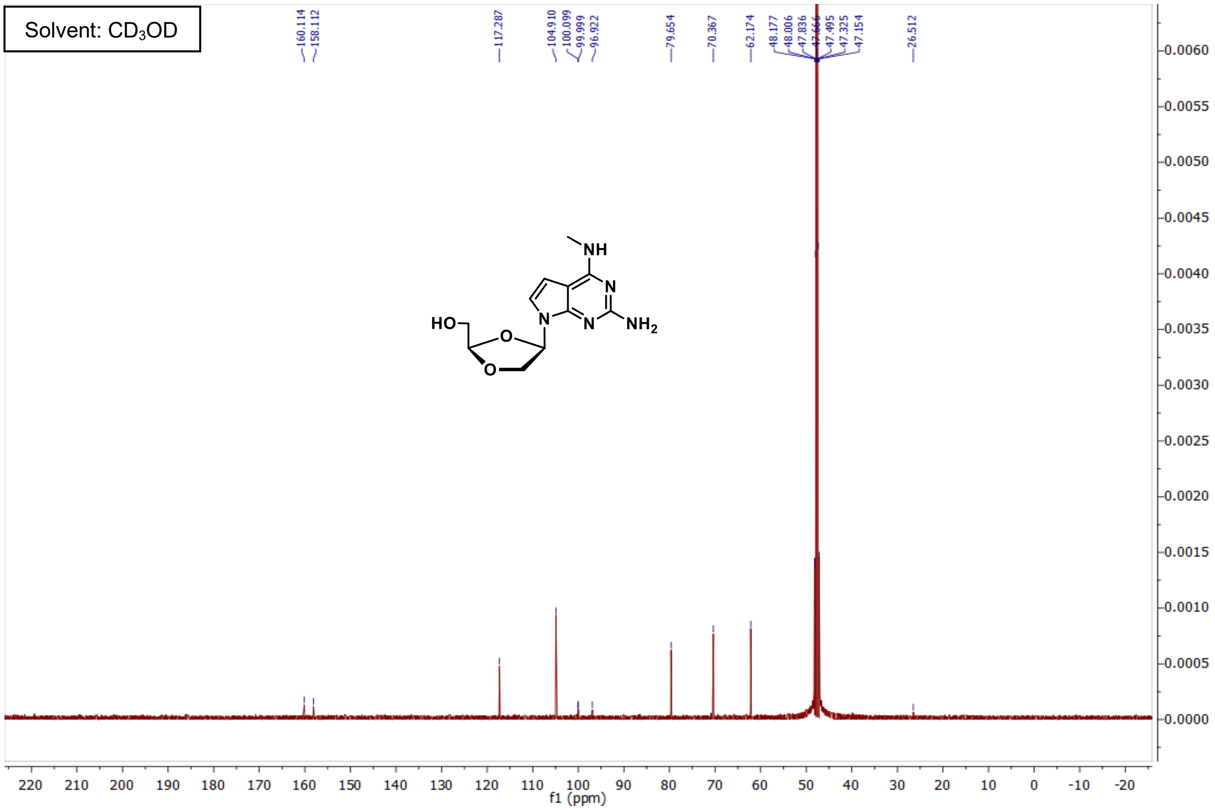

^{13}C NMR of compound **19**

HPLC Method Conditions			
Column	XBridge C18 (4.6x150) mm, 3.5µm	Sample	RJ-02-05
Mobile Phase-A	10mm Ammonium Bicarbonate in Water	Vail	71
Mobile Phase-B	100% Acetonitrile	Injection Volume	1.00 µL
Gradient (T% B)	0/5, 2/5, 10/50, 15/95, 15.1/5, 20/5	Run time	20.0 minutes
Flow Rate	0.8mL/min	Proc. Chnl. Descr.:	2998 PDA MaxPlot (190.0 nm to 800.0 nm) (2998(210-400) nm) Subtracted from blank
Column Oven Temp.	30 °C	Acq. Method Set:	Method 1
Diluent	MeOH+Water		

HPLC analysis of compound **19**

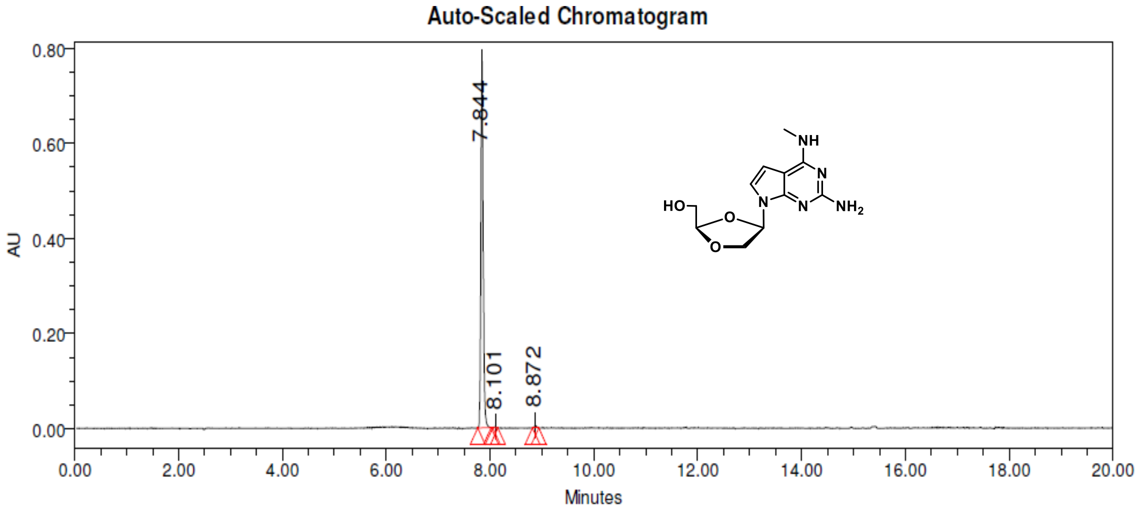

Peak Results

	RT	Area	Height	% Area
1	7.844	2363580	772425	99.26
2	8.101	4819	1874	0.20
3	8.872	12910	5020	0.54

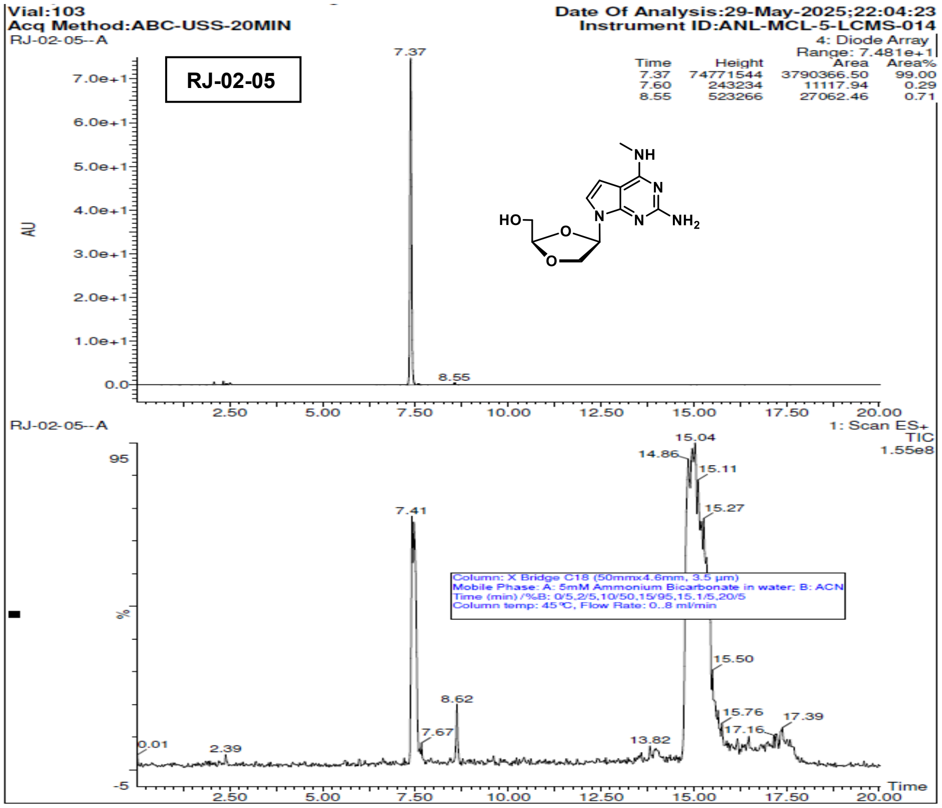

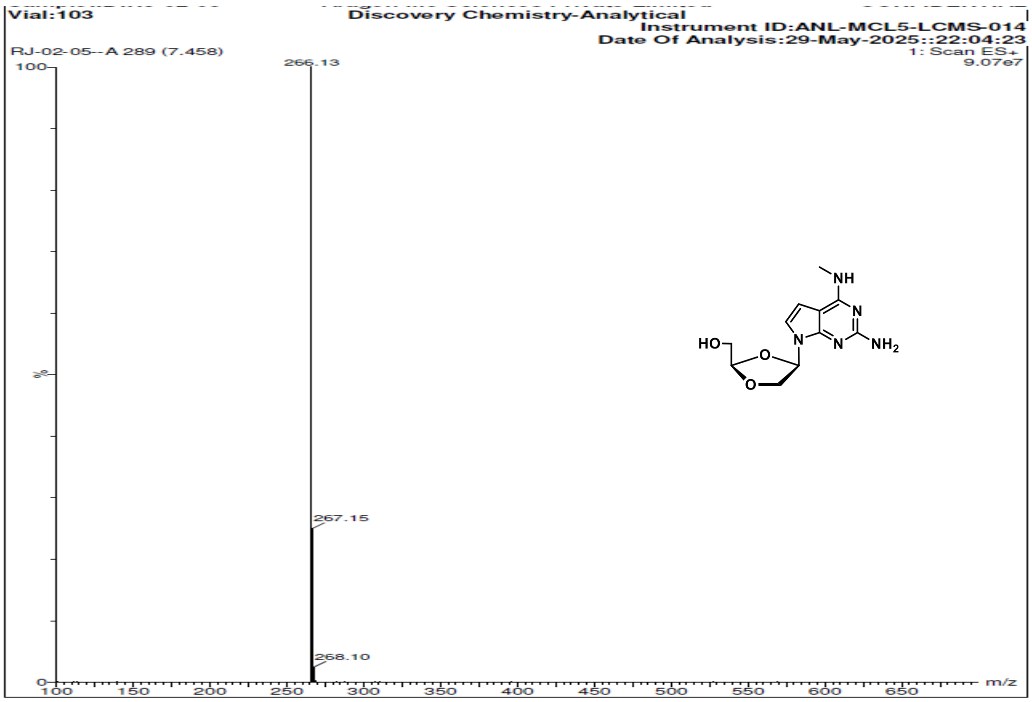

LC-MS analysis of compound **19**

5. X-Ray Crystallographic data of compound 12:

(CCDC#2452696)

Crystals grew from MeOH

Thermal ellipsoid probability levels = 30%

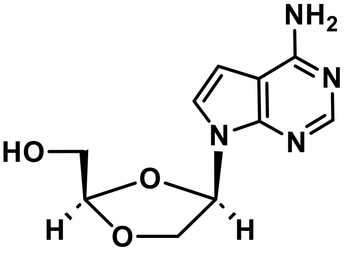

12

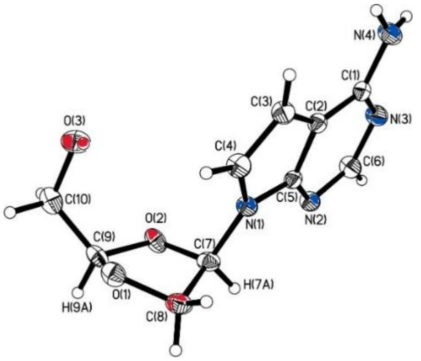

Figure S1: The ORTEP structure of compound **12 (CCDC#2452696)** demonstrates that the hydrogens at C(7) and C(9) are cis to each other, which confirms its relative *β*-stereo conformation.

Table S1. Crystal data and structure refinement for j1_a.

Identification code	j1_a
Empirical formula	C20 H30 N8 O9
Formula weight	526.52
Temperature	299(2) K
Wavelength	0.71073 A
Crystal system, space group	Monoclinic, P2(1)
Unit cell dimensions	a = 7.3020(5) A alpha = 90 deg.<br>b = 14.5417(10) A beta = 96.682(3)deg.<br>c = 11.3016(8) A gamma = 90 deg.
Volume	1191.89(14) A^3
Z, Calculated density	2, 1.467 Mg/m^3

Absorption coefficient	0.117 mm^-1
F(000)	556
Crystal size	0.400 x 0.330 x 0.050 mm
Theta range for data collection	2.292 to 27.877 deg.
Limiting indices	-9<=h<=9, -19<=k<=19, -14<=l<=14
Reflections collected / unique	37086 / 5700 [R(int) = 0.0975]
Completeness to theta = 25.242	99.9 %
Absorption correction	Semi-empirical from equivalents
Max. and min. transmission	0.7457 and 0.4996
Refinement method	Full-matrix least-squares on F^2
Data / restraints / parameters	5700 / 14 / 377
Goodness-of-fit on F^2	1.030
Final R indices [I>2sigma(I)]	R1 = 0.0531, wR2 = 0.1252
R indices (all data)	R1 = 0.0727, wR2 = 0.1365
Absolute structure parameter	-0.2(7)
Extinction coefficient	n/a
Largest diff. peak and hole	0.266 and -0.206 e.A^-3

Table S2. Atomic coordinates (x 10^4) and equivalent isotropic displacement parameters (A^2 x 10^3) for j1_a.
U(eq) is defined as one third of the trace of the orthogonalized Uij tensor.

	x	y	z	U(eq)
O(1)	4834(3)	9528(2)	2006(2)	38(1)
O(2)	6011(3)	8114(2)	1714(2)	38(1)
O(3)	8779(4)	9437(2)	2592(2)	46(1)
O(4)	6994(4)	3006(2)	822(3)	54(1)
O(5)	8093(4)	1636(2)	1526(2)	41(1)
O(6)	10902(4)	3007(2)	1496(3)	54(1)
N(1)	5365(4)	7824(2)	3673(2)	30(1)
N(2)	5503(4)	6170(2)	3571(2)	33(1)
N(3)	6961(4)	5460(2)	5362(3)	38(1)
N(4)	8110(5)	6279(2)	7027(3)	43(1)
N(5)	7475(4)	2248(2)	3356(3)	38(1)
N(6)	7952(4)	798(2)	4377(3)	34(1)
N(7)	9397(4)	945(2)	6388(3)	38(1)
N(8)	10283(5)	2340(3)	7259(3)	53(1)
C(1)	7240(4)	6276(2)	5901(3)	30(1)
C(2)	6646(4)	7085(2)	5306(3)	28(1)
C(3)	6720(5)	8055(2)	5539(3)	34(1)
C(4)	5948(5)	8477(2)	4532(3)	36(1)
C(5)	5805(4)	6976(2)	4141(3)	27(1)
C(6)	6108(5)	5466(2)	4248(3)	39(1)
C(7)	4592(5)	7994(2)	2451(3)	34(1)
C(8)	3494(5)	8880(3)	2292(4)	42(1)
C(9)	5977(5)	9043(2)	1298(3)	33(1)
C(10)	7848(5)	9475(3)	1425(3)	42(1)
C(11)	9508(4)	1861(3)	6294(3)	36(1)
C(12)	8797(4)	2298(2)	5244(3)	34(1)
C(13)	8683(6)	3211(3)	4794(4)	46(1)
C(14)	7905(6)	3160(3)	3653(4)	47(1)
C(15)	8047(4)	1720(2)	4324(3)	31(1)
C(16)	8645(5)	475(3)	5430(3)	37(1)
C(17)	6684(5)	1892(3)	2219(3)	43(1)
C(18)	5606(6)	2605(4)	1418(5)	60(1)
C(19)	8119(6)	2275(3)	569(3)	46(1)
C(20)	10020(6)	2561(3)	466(3)	52(1)
O(7)	4046(6)	5446(5)	1343(4)	95(2)
O(8)	7070(6)	5633(3)	85(4)	78(1)
O(9)	10170(6)	4886(3)	1503(3)	69(1)

Table S3. Bond lengths [A] and angles [deg] for j1_a.

O(1)-C(9)	1.411(4)
O(1)-C(8)	1.422(5)
O(2)-C(7)	1.414(4)
O(2)-C(9)	1.430(4)
O(3)-C(10)	1.412(5)
O(3)-H(3B)	0.82(3)
O(4)-C(19)	1.393(6)
O(4)-C(18)	1.407(6)
O(5)-C(17)	1.414(5)
O(5)-C(19)	1.427(4)
O(6)-C(20)	1.420(5)
O(6)-H(6B)	0.83(3)
N(1)-C(5)	1.366(4)
N(1)-C(4)	1.389(4)
N(1)-C(7)	1.451(4)
N(2)-C(6)	1.322(5)
N(2)-C(5)	1.343(4)
N(3)-C(1)	1.339(5)
N(3)-C(6)	1.339(5)
N(4)-C(1)	1.355(5)
N(4)-H(4B)	0.87(3)
N(4)-H(4C)	0.85(3)
N(5)-C(15)	1.362(5)
N(5)-C(14)	1.395(5)
N(5)-C(17)	1.442(5)
N(6)-C(16)	1.323(5)
N(6)-C(15)	1.344(4)
N(7)-C(16)	1.343(5)
N(7)-C(11)	1.339(5)
N(8)-C(11)	1.360(5)
N(8)-H(8C)	0.86(3)
N(8)-H(8D)	0.88(3)
C(1)-C(2)	1.398(5)
C(2)-C(5)	1.395(5)
C(2)-C(3)	1.436(5)
C(3)-C(4)	1.356(5)
C(3)-H(3A)	0.9300
C(4)-H(4A)	0.9300
C(6)-H(6A)	0.9300
C(7)-C(8)	1.518(5)
C(7)-H(7A)	0.9800
C(8)-H(8A)	0.9700
C(8)-H(8B)	0.9700
C(9)-C(10)	1.495(5)
C(9)-H(9A)	0.9800
C(10)-H(10A)	0.9700
C(10)-H(10B)	0.9700
C(11)-C(12)	1.393(5)

C(12)-C(15)	1.399(5)
C(12)-C(13)	1.421(6)
C(13)-C(14)	1.349(6)
C(13)-H(13A)	0.9300
C(14)-H(14A)	0.9300
C(16)-H(16A)	0.9300
C(17)-C(18)	1.533(6)
C(17)-H(17A)	0.9800
C(18)-H(18A)	0.9700
C(18)-H(18B)	0.9700
C(19)-C(20)	1.466(6)
C(19)-H(19A)	0.9800
C(20)-H(20A)	0.9700
C(20)-H(20B)	0.9700
O(7)-H(7B)	0.86(3)
O(7)-H(7C)	0.85(3)
O(8)-H(8E)	0.86(3)
O(8)-H(8F)	0.89(3)
O(9)-H(9C)	0.87(3)
O(9)-H(9B)	0.85(3)

C(9)-O(1)-C(8)	105.4(3)
C(7)-O(2)-C(9)	109.0(2)
C(10)-O(3)-H(3B)	111(4)
C(19)-O(4)-C(18)	104.9(3)
C(17)-O(5)-C(19)	108.9(3)
C(20)-O(6)-H(6B)	108(4)
C(5)-N(1)-C(4)	107.9(3)
C(5)-N(1)-C(7)	124.7(3)
C(4)-N(1)-C(7)	127.1(3)
C(6)-N(2)-C(5)	111.9(3)
C(1)-N(3)-C(6)	116.8(3)
C(1)-N(4)-H(4B)	112(4)
C(1)-N(4)-H(4C)	120(3)
H(4B)-N(4)-H(4C)	122(5)
C(15)-N(5)-C(14)	107.7(3)
C(15)-N(5)-C(17)	124.5(3)
C(14)-N(5)-C(17)	127.7(3)
C(16)-N(6)-C(15)	112.2(3)
C(16)-N(7)-C(11)	117.8(3)
C(11)-N(8)-H(8C)	115(4)
C(11)-N(8)-H(8D)	115(4)
H(8C)-N(8)-H(8D)	117(5)
N(3)-C(1)-N(4)	117.4(3)
N(3)-C(1)-C(2)	120.3(3)
N(4)-C(1)-C(2)	122.4(3)
C(5)-C(2)-C(1)	115.9(3)
C(5)-C(2)-C(3)	106.7(3)
C(1)-C(2)-C(3)	137.3(3)
C(4)-C(3)-C(2)	106.7(3)
C(4)-C(3)-H(3A)	126.6
C(2)-C(3)-H(3A)	126.6

C(3)-C(4)-N(1)	109.9(3)
C(3)-C(4)-H(4A)	125.0
N(1)-C(4)-H(4A)	125.0
N(2)-C(5)-N(1)	125.8(3)
N(2)-C(5)-C(2)	125.5(3)
N(1)-C(5)-C(2)	108.7(3)
N(2)-C(6)-N(3)	129.6(3)
N(2)-C(6)-H(6A)	115.2
N(3)-C(6)-H(6A)	115.2
O(2)-C(7)-N(1)	110.6(2)
O(2)-C(7)-C(8)	103.8(3)
N(1)-C(7)-C(8)	113.5(3)
O(2)-C(7)-H(7A)	109.6
N(1)-C(7)-H(7A)	109.6
C(8)-C(7)-H(7A)	109.6
O(1)-C(8)-C(7)	102.8(3)
O(1)-C(8)-H(8A)	111.2
C(7)-C(8)-H(8A)	111.2
O(1)-C(8)-H(8B)	111.2
C(7)-C(8)-H(8B)	111.2
H(8A)-C(8)-H(8B)	109.1
O(1)-C(9)-O(2)	106.0(3)
O(1)-C(9)-C(10)	109.2(3)
O(2)-C(9)-C(10)	112.6(3)
O(1)-C(9)-H(9A)	109.6
O(2)-C(9)-H(9A)	109.6
C(10)-C(9)-H(9A)	109.6
O(3)-C(10)-C(9)	114.1(3)
O(3)-C(10)-H(10A)	108.7
C(9)-C(10)-H(10A)	108.7
O(3)-C(10)-H(10B)	108.7
C(9)-C(10)-H(10B)	108.7
H(10A)-C(10)-H(10B)	107.6
N(7)-C(11)-N(8)	118.0(4)
N(7)-C(11)-C(12)	120.1(3)
N(8)-C(11)-C(12)	121.9(4)
C(11)-C(12)-C(15)	115.7(3)
C(11)-C(12)-C(13)	137.1(3)
C(15)-C(12)-C(13)	107.1(3)
C(14)-C(13)-C(12)	107.0(3)
C(14)-C(13)-H(13A)	126.5
C(12)-C(13)-H(13A)	126.5
C(13)-C(14)-N(5)	109.9(4)
C(13)-C(14)-H(14A)	125.1
N(5)-C(14)-H(14A)	125.1
N(6)-C(15)-N(5)	125.9(3)
N(6)-C(15)-C(12)	125.8(3)
N(5)-C(15)-C(12)	108.4(3)
N(6)-C(16)-N(7)	128.5(4)
N(6)-C(16)-H(16A)	115.8
N(7)-C(16)-H(16A)	115.8
O(5)-C(17)-N(5)	110.3(3)

O(5)-C(17)-C(18)	102.0(3)
N(5)-C(17)-C(18)	114.1(4)
O(5)-C(17)-H(17A)	110.1
N(5)-C(17)-H(17A)	110.1
C(18)-C(17)-H(17A)	110.1
O(4)-C(18)-C(17)	102.3(3)
O(4)-C(18)-H(18A)	111.3
C(17)-C(18)-H(18A)	111.3
O(4)-C(18)-H(18B)	111.3
C(17)-C(18)-H(18B)	111.3
H(18A)-C(18)-H(18B)	109.2
O(4)-C(19)-O(5)	106.3(3)
O(4)-C(19)-C(20)	112.7(4)
O(5)-C(19)-C(20)	110.0(3)
O(4)-C(19)-H(19A)	109.3
O(5)-C(19)-H(19A)	109.3
C(20)-C(19)-H(19A)	109.3
O(6)-C(20)-C(19)	114.0(3)
O(6)-C(20)-H(20A)	108.7
C(19)-C(20)-H(20A)	108.7
O(6)-C(20)-H(20B)	108.7
C(19)-C(20)-H(20B)	108.7
H(20A)-C(20)-H(20B)	107.6
H(7B)-O(7)-H(7C)	88(7)
H(8E)-O(8)-H(8F)	75(6)
H(9C)-O(9)-H(9B)	103(7)

Symmetry transformations used to generate equivalent atoms:

Table S4. Anisotropic displacement parameters (A^2 x 10^3) for j1_a.
The anisotropic displacement factor exponent takes the form:
-2 pi^2 [h^2 a*^2 U11 + ... + 2 h k a* b* U12]

	U11	U22	U33	U23	U13	U12
O(1)	45(1)	26(1)	42(1)	0(1)	2(1)	7(1)
O(2)	56(1)	23(1)	33(1)	2(1)	3(1)	10(1)
O(3)	46(1)	45(2)	44(2)	-10(1)	-5(1)	7(1)
O(4)	54(2)	46(2)	58(2)	21(1)	-12(1)	3(1)
O(5)	55(2)	32(1)	34(1)	6(1)	-1(1)	0(1)
O(6)	52(2)	46(2)	60(2)	3(1)	-7(1)	-1(1)
N(1)	36(1)	23(1)	30(1)	-2(1)	-1(1)	-1(1)
N(2)	41(2)	23(1)	33(1)	-4(1)	-3(1)	-6(1)
N(3)	44(2)	27(1)	41(2)	2(1)	-3(1)	-1(1)
N(4)	53(2)	37(2)	36(2)	4(1)	-7(1)	-5(2)
N(5)	41(2)	33(2)	41(2)	2(1)	7(1)	3(1)
N(6)	38(2)	31(2)	33(2)	-3(1)	0(1)	-9(1)
N(7)	42(2)	43(2)	29(2)	-3(1)	1(1)	-6(1)
N(8)	58(2)	57(2)	42(2)	-19(2)	4(2)	-16(2)
C(1)	28(2)	30(2)	33(2)	4(1)	4(1)	-5(1)
C(2)	28(2)	28(2)	27(1)	-2(1)	5(1)	-2(1)
C(3)	42(2)	29(2)	31(2)	-6(1)	1(1)	-5(1)
C(4)	46(2)	23(2)	38(2)	-6(1)	0(2)	-3(1)
C(5)	25(1)	26(2)	31(2)	0(1)	2(1)	-3(1)
C(6)	51(2)	25(2)	40(2)	-2(1)	-1(2)	-3(2)
C(7)	37(2)	29(2)	34(2)	-4(1)	-7(1)	-2(1)
C(8)	38(2)	40(2)	44(2)	2(2)	-6(2)	5(2)
C(9)	50(2)	23(2)	25(2)	0(1)	-3(1)	7(1)
C(10)	57(2)	33(2)	36(2)	-2(2)	9(2)	6(2)
C(11)	29(2)	46(2)	36(2)	-12(2)	10(1)	-10(1)
C(12)	32(2)	33(2)	40(2)	-7(1)	12(1)	-5(1)
C(13)	53(2)	27(2)	60(3)	-11(2)	23(2)	-3(2)
C(14)	54(2)	30(2)	58(2)	2(2)	17(2)	2(2)
C(15)	27(2)	32(2)	35(2)	-2(1)	8(1)	-3(1)
C(16)	45(2)	31(2)	36(2)	-2(1)	3(2)	-6(1)
C(17)	37(2)	43(2)	47(2)	8(2)	-3(2)	-6(2)
C(18)	44(2)	66(3)	67(3)	18(2)	-12(2)	6(2)
C(19)	68(2)	37(2)	28(2)	7(2)	-12(2)	-8(2)
C(20)	72(3)	52(2)	33(2)	7(2)	8(2)	-1(2)
O(7)	85(3)	147(5)	51(2)	-31(3)	-1(2)	-17(3)
O(8)	79(2)	81(3)	69(2)	-9(2)	-8(2)	-15(2)
O(9)	90(3)	56(2)	57(2)	-15(2)	-13(2)	10(2)

Table S5. Hydrogen coordinates (x 10^4) and isotropic displacement parameters (A^2 x 10^3) for j1_a.

	x	y	z	U(eq)
H(3B)	8230(70)	9730(30)	3060(40)	59(15)
H(6B)	10470(70)	3530(20)	1510(50)	60
H(4B)	8640(60)	5760(20)	7210(50)	61(14)
H(4C)	8500(50)	6780(20)	7350(40)	39(10)
H(8C)	10960(60)	2020(30)	7780(40)	65
H(8D)	10690(70)	2890(20)	7100(50)	78(18)
H(3A)	7204	8340	6244	41
H(4A)	5826	9110	4432	43
H(6A)	5911	4890	3898	47
H(7A)	3810	7474	2160	41
H(8A)	2494	8827	1652	50
H(8B)	2993	9050	3020	50
H(9A)	5443	9064	462	40
H(10A)	8595	9168	888	50
H(10B)	7724	10113	1182	50
H(13A)	9071	3743	5206	55
H(14A)	7687	3659	3140	56
H(16A)	8605	-160	5517	45
H(17A)	5897	1362	2336	52
H(18A)	4669	2315	862	72
H(18B)	5028	3058	1885	72
H(19A)	7601	1982	-176	55
H(20A)	10735	2023	303	63
H(20B)	10015	2974	-208	63
H(7B)	2910(50)	5300(60)	1390(70)	105
H(7C)	4340(100)	5080(50)	1910(60)	110(30)
H(8E)	6450(90)	5530(60)	-600(40)	105
H(8F)	5910(50)	5570(50)	230(80)	105
H(9C)	9140(60)	5060(50)	1090(60)	100(20)
H(9B)	10060(90)	5090(50)	2200(30)	90(20)

Table S6. Torsion angles [deg] for j1_a.

C(6)-N(3)-C(1)-N(4)	-179.7(3)
C(6)-N(3)-C(1)-C(2)	0.3(5)
N(3)-C(1)-C(2)-C(5)	-1.2(4)
N(4)-C(1)-C(2)-C(5)	178.8(3)
N(3)-C(1)-C(2)-C(3)	-177.7(4)
N(4)-C(1)-C(2)-C(3)	2.3(6)
C(5)-C(2)-C(3)-C(4)	-0.2(4)
C(1)-C(2)-C(3)-C(4)	176.5(4)
C(2)-C(3)-C(4)-N(1)	0.7(4)
C(5)-N(1)-C(4)-C(3)	-0.9(4)
C(7)-N(1)-C(4)-C(3)	-175.3(3)
C(6)-N(2)-C(5)-N(1)	178.9(3)
C(6)-N(2)-C(5)-C(2)	-0.1(4)
C(4)-N(1)-C(5)-N(2)	-178.3(3)
C(7)-N(1)-C(5)-N(2)	-3.8(5)
C(4)-N(1)-C(5)-C(2)	0.8(3)
C(7)-N(1)-C(5)-C(2)	175.3(3)
C(1)-C(2)-C(5)-N(2)	1.2(5)
C(3)-C(2)-C(5)-N(2)	178.7(3)
C(1)-C(2)-C(5)-N(1)	-177.9(3)
C(3)-C(2)-C(5)-N(1)	-0.4(3)
C(5)-N(2)-C(6)-N(3)	-1.1(5)
C(1)-N(3)-C(6)-N(2)	1.1(6)
C(9)-O(2)-C(7)-N(1)	-113.2(3)
C(9)-O(2)-C(7)-C(8)	8.8(3)
C(5)-N(1)-C(7)-O(2)	-88.8(3)
C(4)-N(1)-C(7)-O(2)	84.6(4)
C(5)-N(1)-C(7)-C(8)	155.0(3)
C(4)-N(1)-C(7)-C(8)	-31.5(5)
C(9)-O(1)-C(8)-C(7)	36.5(3)
O(2)-C(7)-C(8)-O(1)	-27.6(3)
N(1)-C(7)-C(8)-O(1)	92.5(3)
C(8)-O(1)-C(9)-O(2)	-31.8(3)
C(8)-O(1)-C(9)-C(10)	-153.4(3)
C(7)-O(2)-C(9)-O(1)	13.5(3)
C(7)-O(2)-C(9)-C(10)	132.9(3)
O(1)-C(9)-C(10)-O(3)	63.0(4)
O(2)-C(9)-C(10)-O(3)	-54.5(4)
C(16)-N(7)-C(11)-N(8)	-179.5(3)
C(16)-N(7)-C(11)-C(12)	2.3(5)
N(7)-C(11)-C(12)-C(15)	-2.0(4)
N(8)-C(11)-C(12)-C(15)	179.8(3)
N(7)-C(11)-C(12)-C(13)	-178.1(4)
N(8)-C(11)-C(12)-C(13)	3.7(6)
C(11)-C(12)-C(13)-C(14)	176.0(4)
C(15)-C(12)-C(13)-C(14)	-0.3(4)
C(12)-C(13)-C(14)-N(5)	1.2(4)
C(15)-N(5)-C(14)-C(13)	-1.7(4)

C(17)-N(5)-C(14)-C(13)	-177.9(3)
C(16)-N(6)-C(15)-N(5)	178.9(3)
C(16)-N(6)-C(15)-C(12)	0.7(5)
C(14)-N(5)-C(15)-N(6)	-176.9(3)
C(17)-N(5)-C(15)-N(6)	-0.6(5)
C(14)-N(5)-C(15)-C(12)	1.5(4)
C(17)-N(5)-C(15)-C(12)	177.8(3)
C(11)-C(12)-C(15)-N(6)	0.4(5)
C(13)-C(12)-C(15)-N(6)	177.7(3)
C(11)-C(12)-C(15)-N(5)	-178.0(3)
C(13)-C(12)-C(15)-N(5)	-0.7(3)
C(15)-N(6)-C(16)-N(7)	-0.5(5)
C(11)-N(7)-C(16)-N(6)	-1.0(5)
C(19)-O(5)-C(17)-N(5)	-109.0(3)
C(19)-O(5)-C(17)-C(18)	12.5(4)
C(15)-N(5)-C(17)-O(5)	-84.5(4)
C(14)-N(5)-C(17)-O(5)	91.1(4)
C(15)-N(5)-C(17)-C(18)	161.5(3)
C(14)-N(5)-C(17)-C(18)	-23.0(5)
C(19)-O(4)-C(18)-C(17)	39.9(4)
O(5)-C(17)-C(18)-O(4)	-31.8(4)
N(5)-C(17)-C(18)-O(4)	87.1(4)
C(18)-O(4)-C(19)-O(5)	-32.9(4)
C(18)-O(4)-C(19)-C(20)	-153.5(3)
C(17)-O(5)-C(19)-O(4)	11.5(4)
C(17)-O(5)-C(19)-C(20)	133.8(4)
O(4)-C(19)-C(20)-O(6)	57.6(5)
O(5)-C(19)-C(20)-O(6)	-60.8(5)

Symmetry transformations used to generate equivalent atoms:

Table S7. Hydrogen bonds for j1_a [A and deg.].

D-H...A	d(D-H)	d(H...A)	d(D...A)	<(DHA)
O(3)-H(3B)...N(6)#1	0.82(3)	2.17(3)	2.939(4)	155(5)
O(6)-H(6B)...O(9)	0.83(3)	1.98(3)	2.785(5)	164(5)
N(4)-H(4C)...O(6)#2	0.85(3)	2.22(3)	3.056(5)	167(4)
N(8)-H(8D)...O(3)#3	0.88(3)	2.30(4)	3.126(5)	156(5)
O(7)-H(7B)...O(9)#4	0.86(3)	2.11(3)	2.970(7)	177(8)
O(9)-H(9C)...O(8)	0.87(3)	1.97(3)	2.834(6)	170(7)
O(9)-H(9B)...N(7)#2	0.85(3)	2.03(4)	2.825(5)	155(7)

Symmetry transformations used to generate equivalent atoms:
#1 x,y+1,z   #2 -x+2,y+1/2,-z+1   #3 -x+2,y-1/2,-z+1
#4 x-1,y,z

References:

(1) Apweiler, R.; Bairoch, A.; Wu, C. H.; Barker, W. C.; Boeckmann, B.; Ferro, S.; Gasteiger, E.; Huang, H. Z.; Lopez, R.; Magrane, M.; Martin, M. J.; Natale, D. A.; O'Donovan, C.; Redaschi, N.; Yeh, L. S. L. UniProt: the Universal Protein knowledgebase. *Nucleic Acids Res.* **2004**, *32*, D115–D119.

(2) Gustavsson, E.; Grünewald, K.; Elias, P.; Hällberg, B. M. Dynamics of the Herpes simplex virus DNA polymerase holoenzyme during DNA synthesis and proof-reading revealed by Cryo-EM. *Nucleic Acids Res.* **2024**, *52* (12), 7292–7304.

(3) Yang, Y.; Yao, K.; Repasky, M. P.; Leswing, K.; Abel, R.; Shoichet, B. K.; Jerome, S. Efficient Exploration of Chemical Space with Docking and Deep Learning. *J. Chem. Theory Comput.* **2021**, *17* (11), 7106–7119.
